# Technology‐based and digital interventions for intimate partner violence: A systematic review and meta‐analysis

**DOI:** 10.1002/cl2.1271

**Published:** 2022-08-27

**Authors:** Chuka Emezue, Jo‐Ana D. Chase, Tipparat Udmuangpia, Tina L. Bloom

**Affiliations:** ^1^ Department of Women, Children and Family Nursing Rush University College of Nursing Chicago Illinois USA; ^2^ Sinclair School of Nursing University of Missouri Columbia Missouri USA; ^3^ Department of Maternal‐Child Health and Midwifery Boromarajonani College of Nursing Khon Kaen Thailand; ^4^ School of Nursing Notre Dame of Maryland University Baltimore Maryland USA

## Abstract

**Background:**

A growing body of research shows the promise and efficacy of technology‐based or digital interventions in improving the health and well‐being of survivors of intimate partner violence (IPV). In addition, mental health comorbidities such as anxiety, post‐traumatic stress disorder (PTSD), and depression occur three to five times more frequently in survivors of IPV than non‐survivors, making these comorbidities prominent targets of technology‐based interventions. Still, research on the long‐term effectiveness of these interventions in reducing IPV victimization and adverse mental health effects is emergent. The significant increase in the number of trials studying technology‐based therapies on IPV‐related outcomes has allowed us to quantify the effectiveness of such interventions for mental health and victimization outcomes in survivors. This meta‐analysis and systematic review provide critical insight from several randomized controlled trials (RCTs) on the overall short and long‐term impact of technology‐based interventions on the health and well‐being of female IPV survivors.

**Objectives:**

To synthesize current evidence on the effects of technology‐based or digital interventions on mental health outcomes (depression, anxiety, and PTSD) and victimization outcomes (physical, psychological, and sexual abuse) among IPV survivors.

**Search Methods:**

We examined multiple traditional and grey databases for studies published from 2007 to 2021. Traditional databases (such as PubMed Central, Web of Science, CINAHL Plus, and PsychINFO) and grey databases were searched between April 2019 and February 2021. In addition, we searched clinical trial registries, government repositories, and reference lists. Authors were contacted where additional data was needed. We identified 3210 studies in traditional databases and 1257 from grey literature. Over 2198 studies were determined to be duplicates and eliminated, leaving 64 studies after screening titles and abstracts. Finally, 17 RCTs were retained for meta‐analysis. A pre‐registered protocol was developed and published before conducting this meta‐analysis.

**Selection Criteria:**

We included RCTs targeting depression, anxiety, PTSD outcomes, and victimization outcomes (physical, sexual, and psychological violence) among IPV survivors using a technology‐based intervention. Eligible RCTs featured a well‐defined control group. There were no study restrictions based on participant gender, study setting, or follow‐up duration. Included studies additionally supplied outcome data for calculating effect sizes for our desired outcome. Studies were available in full text and published between 2007 and 2021 in English.

**Data Collection and Analysis:**

We extracted relevant data and coded eligible studies. Using Cochrane's RevMan software, summary effect sizes (*Outcome by Time*) were assessed using an independent fixed‐effects model. Standardized mean difference (SMD) effect sizes (or Cohen's *d*) were evaluated using a Type I error rate and an alpha of 0.05. The overall intervention effects were analyzed using the *Z*‐statistic with a *p*‐value of 0.05. Cochran's *Q* test and Higgins' *I* ^2^ statistics were utilized to evaluate and confirm the heterogeneity of each cumulative effect size. The Cochrane risk of bias assessment for randomized trials (RoB 2) was used to assess the quality of the studies. Campbell Systematic Reviews registered and published this study's protocol in January 2021. No exploratory moderator analysis was conducted; however, we report our findings with and without outlier studies in each meta‐analysis.

**Main Results:**

Pooled results from 17 RCTs yielded 18 individual effect size comparisons among 4590 survivors (all females). Survivors included college students, married couples, substance‐using women in community prisons, pregnant women, and non‐English speakers, and sample sizes ranged from 15 to 672. Survivors' ages ranged from 19 to 41.5 years. Twelve RCTs were conducted in the United States and one in Canada, New Zealand, China (People's Republic of), Kenya, and Australia. The results of this meta‐analysis found that technology‐based interventions significantly reduced *depression* among female IPV survivors at 0–3 months only (SMD = −0.08, 95% confidence interval [CI] = −0.17 to −0.00), *anxiety* among IPV survivors at 0–3 months (SMD = −0.27, 95% CI = −0.42 to −0.13, *p* = 0.00, *I* ^2^ = 25%), and *physical violence victimization* among IPV survivors at 0–6 months (SMD = −0.22, 95% CI = −0.38 to −0.05). We found significant reductions in psychological violence victimization at 0–6 months (SMD = −0.34, 95% CI = −0.47 to −0.20) and at >6 months (SMD = −0.29, 95% CI = −0.39 to −0.18); however, at both time points, there were outlier studies. At no time point did digital interventions significantly reduce *PTSD* (SMD = −0.04, 95% CI = −0.14 to 0.06, *p* = .46, *I* ^2^ = 0%), or *sexual violence victimization* (SMD = −0.02, 95% CI = −0.14 to 0.11, *I* ^2^ = 21%) among female IPV survivors for all. With outlier studies removed from our analysis, all summary effect sizes were small, and this small number of comparisons prevented moderator analyses.

**Authors' Conclusions:**

The results of this meta‐analysis are promising. Our findings highlight the effectiveness of IPV‐mitigating digital intervention as an add‐on (not a replacement) to traditional modalities using a coordinated response strategy. Our findings contribute to the current understanding of “what works” to promote survivors' mental health, safety, and well‐being. Future research could advance the science by identifying active intervention ingredients, mapping out intervention principles/mechanisms of action, best modes of delivery, adequate dosage levels using the treatment intensity matching process, and guidelines to increase feasibility and acceptability.

## 1 PLAIN LANGUAGE SUMMARY

### 1.1 Technology‐based interventions reduce depression, anxiety and physical violence victimization among intimate partner violence survivors in the short term

Compared to non‐technology‐based interventions for survivors of intimate partner violence (IPV), technology‐based interventions are effective in reducing adverse mental health and IPV outcomes. We found effect sizes that were small to moderate that should be interpreted with care.

### 1.2 What is this review about?

The spread of IPV digital interventions provides a compelling reason to collect evidence of their intervention and treatment effects. Technology‐based therapies come in many forms, including phone and web‐based decision aids, conversational agents (chatbots), text message interventions, online support groups, and telehealth services.

Although technology‐based therapies have become acceptable, practical and feasible for supporting the health and well‐being of IPV survivors, little is known about the size of their cumulative effects on IPV survivors' health and well‐being.

Furthermore, it is unknown how much, for whom and how long these effects last. The extent to which the type of digital intervention (smartphone vs. web‐based) contributes to this impact is also unknown.

This review and meta‐analysis aim to fill these gaps in our understanding. Clarity on the pros and cons of digital IPV interventions have implications for intervention design, user engagement and adoption among IPV survivors. Only a few digital IPV therapies have been tested in (sub‐optimal) “real‐world” situations. Even fewer attempts have been made to cumulate the intervention impact of digital interventions on survivors' mental health, despite the commonality of depression, anxiety disorders and post‐traumatic stress disorder (PTSD) among IPV survivors.

**What is the aim of this review?**

This Campbell systematic review examines the effect of technology‐based or digital interventions on the mental health outcomes—depression, anxiety and post‐traumatic stress disorder (PTSD)—and victimization (physical, psychological, and sexual abuse) of intimate partner violence (IPV) survivors.

### 1.3 What studies are included?

We analyzed 17 experimental studies (randomized controlled trials), each with well‐defined control groups. The studies were published between 2007 and 2021, most published in 2016. Twelve of the 17 studies were conducted in the USA. One study each was included from Canada, Australia, New Zealand, Kenya and China. Most studies had a moderate risk of bias.

### 1.4 What are the main findings of this review?

Results from randomized controlled trials indicate that digital and technology‐based interventions significantly reduce depression (up to 3 months), anxiety (up to 3 months), and physical violence victimization (at 6 months post‐intervention) among female IPV survivors. Results from studies on psychological violence victimization are inconclusive.

These effects, however, appear to fade over time for these outcomes. Also, the same digital interventions have no significant effect on PTSD or sexual violence victimization experiences at any time point.

Overall, digital treatments provide concrete benefits in terms of providing survivors with meaningful support, even if only temporarily, especially during increased emotional, mental and relationship distress.

### 1.5 What do the findings of the review mean?

This systematic review finds that digital interventions work. Intervention funders and violence prevention policymakers can use these results to set a baseline effect size for IPV digital interventions. These results can also inform health policy, to support providers' reimbursement for offering or recommending digital interventions backed by evidence.

Results from the meta‐analysis can be used to bolster calls for the inclusion of IPV digital therapies as add‐on therapeutic devices during routine IPV screening of girls and women (ages 14–46 years).

These findings also help service providers to decide if digital approaches are beneficial, dependable and safe for assisting survivors' emotional well‐being.

### 1.6 How up‐to‐date is this review?

The review authors searched for studies from 2007 to 2021.

## 2 BACKGROUND

Intimate partner violence (IPV) remains a persistent public health, social justice, and human rights concern. IPV has numerous well‐known effects on the physical, social, emotional, and economic condition of survivors, their families, and communities (Black et al., 2011; Breiding et al., 2014; Nathanson et al., 2012). However, the mental health consequences of experiencing partner abuse might be the most severe and longest‐lasting. Several studies show moderate to strong positive correlations between experiencing IPV and depression and anxiety (Beydoun et al., 2012; Mechanic et al., 2008; Tol et al., 2019). Furthermore, 30%‐80% of IPV survivors meet criteria for post‐traumatic stress disorder (PTSD) (Nathanson et al., 2012).

In some meta‐analyses, IPV survivors are three to five times more likely to report depression, anxiety, and PTSD symptoms than non‐survivors (Golding, 1999; Lagdon et al., 2014). In addition, untreated mental health issues limit survivors' access to medical care and treatment, create barriers to holistic therapy, diminish their overall quality of life, and can foster a culture of chronic poly‐victimization and co‐morbidities among survivors (Johnson et al., 2011; Johnson & Zlotnick, 2009; Nathanson et al., 2012). Therefore, mental health and victimization outcomes remain stable targets for current interventions (Ellsberg et al., 2015; Gevers & Dartnall, 2014; Lagdon et al., 2014; Sharhabani‐Arzy et al., 2005).

Technology‐based interventions have become acceptable, convenient, and feasible for supporting the health and well‐being of IPV survivors. These technology‐based therapies come in many forms, such as smartphone apps, phone and web‐based decision aids, chatbots or conversational agents, text message interventions, web‐based online support groups, social media, and telehealth services (Bloom et al., 2016; Debnam & Kumodzi, 2019; Klevens et al., 2012; Koziol‐McLain et al., 2016; May et al., 2003; Young et al., 2018). In addition, digital interventions are a cost‐saving, provider‐mediating, and scalable bargain, particularly where they augment in‐person modalities (like group counseling, face‐to‐face therapy, and psychosocial‐behavioral therapies) (Campbell, 2002) for assisting IPV survivors. These interventions overcome coverage gaps caused by health system problems and inequities, particularly in areas where health provider shortages exist and IPV victimization overlap with social determinants of violence (e.g., socioeconomic status, rurality, immigration status, strict gender norms, racial and ethnic disparities, and disabilities) (Akinsulure‐Smith et al., 2013; García‐Moreno et al., 2013). Moreover, these digital interventions serve a vital and timely function, as they offer social and emotional support for those who have recently experienced IPV by a partner or ex‐partner, typically at a time of high distress and crisis.

To their merit, digital interventions may be a viable source of support for historically marginalized survivors who report an elevated risk of IPV victimization, including Black and Latinx, immigrant, refugee, and asylum‐seeking women, as well as victims‐survivors with disabilities, rural women, older women, women and girls in low‐ or middle‐income regions, First Nation and Indigenous Women, and LGBTQIA+ individuals (Akinsulure‐Smith et al., 2013; Black et al., 2011; García‐Moreno et al., 2013; Koziol‐McLain et al., 2018; Malley‐Morrison & Hines, 2004; Peterson et al., 2018; Raj & Silverman, 2002).

Therefore, this systematic review and meta‐analyses aim to expand our current understanding of how effectively these digital interventions reduce mental health issues (depression, anxiety, and PTSD) and IPV victimization (physical, psychological, and sexual victimization). In addition, the significant increase in the number of clinical trials studying technology‐based therapies on IPV‐related outcomes has allowed us to quantify the effectiveness of such interventions for mental health and victimization outcomes in survivors.

### 2.1 Description of the condition

IPV remains a persistent public health, social justice, and human rights concern. Regardless of intimacy, relationship type, gender, or sexual orientation, the most common forms of partner violence include physical violence, sexual assault, psychological aggression, and stalking by a current or former partner or spouse (McFarlane et al., 2002; World Health Organization, 2019). About one in four US women (one in three globally) and almost one in nine US males report having experienced sexual assault, physical violence, or stalking by an intimate relationship at some point in their lives (García‐Moreno et al., 2013; Tjaden & Thoennes, 1998).

### 2.2 Description of the intervention

Technology‐based interventions comprise eHealth (or electronic health), mHealth (or mobile health), and telehealth platforms used to deliver health care services and collect and share clinical data (World Health Organization, 2019). Over half a billion people worldwide use a health‐related smartphone app (Dorsey et al., 2017). This proliferation of health‐facing apps now empowers people to generate, monitor, and control their health data (Anderson‐Lewis et al., 2018; Dorsey et al., 2017; Klasnja & Pratt, 2012). Likewise, rapid improvements in patient technology literacy, device ownership, and device features have led to a growing reliance on technology to deliver interventions to support IPV survivors in ecologically valid ways while providing 24/7, safe, and confidential services and resources (Anderson‐Lewis et al., 2018; Glass et al., 2010, 2017; Ranney et al., 2013). More recently, technology‐based interventions have gained acceptance due to widespread disruptions in support systems for partner violence survivors during the COVID‐19 pandemic (Emezue 2020).

Clinically, several meta‐analyses show the promise of digital interventions in addressing multiple issues, ranging from problematic substance use to physical inactivity to diabetes self‐management across diverse cohorts (Anderson‐Lewis et al., 2018; Firth et al., 2017; Free et al., 2010; Khadjesari et al., 2011; Li et al., 2014; Liang et al., 2011; Luk et al., 2019; Montgomery et al., 2017; Ybarra et al., 2016). Moreover, over the past three decades, digital therapies, at various levels of sophistication, have been created and adapted to support the health and well‐being of IPV survivors, making these interventions a critical layer of support for survivors.

Feasibility and acceptability studies show IPV survivors are receptive to trauma‐informed technology‐based interventions, and multiple individual studies—several of them reviewed in this study ‐ demonstrate the efficacy of these interventions (Glass et al., 2017; Koziol‐McLain et al., 2016; Littleton et al., 2016). In certain instances, technology outperforms traditional modalities. For example, a systematic review by Hussain et al. (2015) found that self‐administered computer screening outperformed face‐to‐face screening for IPV by 37% and paper and pencil screeners by 23%, leading to higher rates of IPV disclosure from technology‐optimized strategies. In addition, digital IPV interventions are purposefully designed to mitigate some shortcomings of traditional interventions, such as cost‐of‐care barriers, non‐confidentiality, poorly trained health/service providers, geographical inaccessibility, stigmas associated with seeking care (e.g., rape and sexual assault services), and socio‐geographical diversity (Constantino et al., 2015; Eden et al., 2015; Ford‐Gilboe et al., 2017; Glass et al., 2010, 2017; Koziol‐McLain et al., 2018; Littleton et al., 2016).

Few reviews have examined the effectiveness of digital therapies in reducing IPV‐related mental health disorders in survivors (Fu et al., 2020). However, early results are promising. From 22 studies, Fu et al. (2020) observed that digital psychological therapies were somewhat effective in addressing mental health outcomes compared to non‐digital controls for adults in low‐ and middle‐income countries (Hedges' *g* = 0.60 [95% confidence interval [CI]: 0.45–0.75]). El Morr and Layal (2020) also reviewed 25 studies evaluating the efficacy, acceptability, and applicability of these information and communication technologies (ICT) for addressing different IPV outcomes (e.g., awareness, screening, prevention, treatment, and mental health), finding substantial differences in study outcome measures, sample sizes, study design, intervention type, and participant characteristics.

Nonetheless, evidence of the effectiveness of digital interventions in enhancing the mental health and victimization outcomes of IPV survivors is just beginning to emerge. This is partly due to the novelty of digital interventions and our limited understanding of their short‐ and long‐term effects on different types of IPV survivors. Nevertheless, the proliferation of randomized trials pilot‐testing these interventions, the widening epidemic of partner violence, and the diversity of survivor needs provide compelling reasons to compile empirical evidence to provide a clear picture of the effectiveness of these interventions for survivors.

### 2.3 How the intervention might work

Digital IPV interventionists borrow liberally from social and behavioral sciences to develop and adapt IPV digital interventions. The most common theories that underlie these digital interventions include feminist theories (e.g., gender and power theory), social cognitive theory, family systems theories, theories of technology adoption (Emezue, 2020), trauma‐informed care, and harm reduction, all ingrained within a social‐ecological context (Lawson, 2012). We further discuss some of these theories in the “Summary of main results” section.

Digital IPV interventions may work in the following ways:

1. offering ongoing or one‐time social and emotional support,
2. increasing individualized safety planning and risk assessment of frequency, severity, and types of violence,
3. providing information and psychoeducation to improve informed victim‐centered decision‐making,
4. improving evidence gathering and documentation,
5. reducing exposure to future IPV (triage to local and trusted services),
6. improving mental and emotional health outcomes (depressive and anxiety disorders),
7. addressing co‐occurring morbidities linked to IPV (e.g., substance use, housing, and employment needs), and
8. triaging survivors to trusted care based on unique social and personal contexts.

In some instances, the technology itself *is* the intervention (i.e., “technology‐based interventions”) or is merely used to deliver evidence‐based interventions (“technology‐enhanced interventions”). Our meta‐analysis looks at all such descriptions of *technology*, so long as they were designed to support the health and well‐being of IPV survivors.

### 2.4 Why it is important to do this review

The proliferation of IPV digital intervention presents a strong rationale for gathering evidence on the intervention and treatment effects of these interventions to address critical gaps in our current understanding. Moreover, a clear understanding of the benefits (and drawbacks) of digital IPV interventions bears serious implications for intervention design, user engagement, uptake, and overall treatment effects for IPV survivors.

At the study level, we know that digital interventions are efficacious (Ford‐Gilboe et al., 2017; Glass et al., 2010, 2017). However, the extent to which they contribute to long‐term improvements in survivor outcomes remains unknown (Ford‐Gilboe et al., 2017). Few IPV digital interventions have reached effectiveness and pragmatic trials to test their effectiveness in sub‐optimal real‐world conditions. In addition, there have been few attempts to cumulate the intervention (or treatment) effects of digital interventions on survivors' mental health, even though mental health outcomes such as depression, anxiety disorders, and PTSD are primary concerns for survivors (Keynejad et al., 2020; Lagdon et al., 2014; Nathanson et al., 2012).

Furthermore, funders and policymakers can use findings from this and other meta‐analytic studies to establish an omnibus effect size baseline for digital interventions targeting key IPV and mental health outcomes. The findings of this analysis could lead to changes in health policy that allow providers to be reimbursed for offering or recommending evidence‐based digital interventions. In addition, results from this meta‐analysis can be used to bolster calls for the inclusion of IPV digital therapies as additional therapeutic tools in universal screening systems in keeping with the US Preventive Services Task Force (USPSTF) for routine screening of reproductive‐age women (14–46 years) (Moyer, 2013). Our findings will help evidence‐to‐decision frameworks for service providers who want to know if digital approaches are effective, reliable, and safe for supporting the emotional well‐being of survivors.

Finally, during the COVID‐19 pandemic, stay‐at‐home and social distancing mandates disrupted regular service provision for survivors. This led to digital therapies becoming even more pertinent, as epidemiology data reported surges in domestic violence during this time (Emezue, 2020). Overall, a meta‐analysis remains one of the highest levels of evidence to support practice, research, and policy guidelines.

## 3 OBJECTIVES

This review and meta‐analysis aim to synthesize current evidence on the effectiveness of digital interventions on mental health and victimization outcomes among survivors of partner violence.

The following research questions guided this study:

1. Are digital and technology‐based interventions effective in reducing mental health issues (depression, anxiety, and PTSD) following the intervention and at later follow‐up?
2. Are digital and technology‐based interventions effective in reducing IPV victimization (physical, psychological, and sexual victimization) following the intervention and at later follow‐up?

### 3.1 Title registration and review protocol

The Campbell Collaboration approved the title for this systematic review on 23 May 2019. The review protocol was published on January 14, 2021. The title registration and protocol are available at: https://onlinelibrary.wiley.com/doi/full/10.1002/cl2.1132

## 4 METHODS

### 4.1 Criteria for considering studies for this review

#### 4.1.1 Types of studies

##### Inclusion criteria

In Table 1, we show a breakdown of our eligibility criteria. Studies were eligible if they (1) used a randomized controlled trial (RCT) with a well‐defined treatment and control groups; (2) included outcome data to compute effect sizes for PTSD, anxiety, depression, and victimization (physical, psychological, and sexual violence); (3) were full‐text available; (4) published in English (due to the authors' language limitations); and (5) published between 2007 and 2021 (to account for the previous 15 years, during which time the popularity of digital therapies for IPV significantly rose (Anderson et al., 2019; Anderson‐Lewis et al., 2018). Studies in which IPV victimization outcomes were secondary outcomes were also included.

**Table 1:** Study eligibility criteria

Study characteristic	Inclusion criteria	Exclusion criteria
Population	Survivors of IPV or violence in relational contexts	Children and adolescents under 18 Perpetrators
Intervention	Digital interventions: Defined broadly to include any use of technology to deliver information, treatment, therapy, or psychosocial support to improve the mental health of survivors of partner violence. All types of IPV digital interventions (mHealth, eHealth, and telehealth) designed to reduce IPV and related mental health outcomes	Non‐digital methods (e.g. paper and pencil surveys, checklists), traditional modalities (e.g. counseling, advocacy group sessions, standard shelter services, home visitation). Studies were also excluded where the extent of technology use in the study was limited to participant randomization, recruitment, screening only, follow‐up only. Studies were also excluded without a clear characterization of IPV outcomes. Pilot studies that focused on feasibility and acceptability only were excluded.
Comparator	Control conditions can involve: Usual care (UC), No treatment, Intervention ‐as‐usual (IAU), Waitlist controls, Active placebo control group.	No comparator
Outcomes	Eligible digital and technology‐based interventions must address a mental health conditions	Non‐IPV Domestic violence encompassing child abuse, peer violence, acquaintance rape, elder abuse, bullying, parental violence, and other non‐dating victimization
	o Depression, or any clinical/base change in depressive symptoms. o Post‐traumatic stress disorder (PTSD), or any clinical/base change in PTSD symptoms. o Anxiety, or any clinical/base change in anxiety symptoms.	
	Forms of IPV victimization and any changes in IPV victimization outcomes:	
	o Physical abuse o Sexual violence o Psychological abuse	
Timing	No limitations were placed on studies by their duration of follow‐up.	None
Setting	Any community‐based, school, online, religious, or clinical setting Global, any country.	None
Study design	Randomized control trials (RCTs), with randomization to control and intervention conditions. Quasi‐randomized control trials, where a quasi‐random method of allocation was used, (e.g., the order of recruitment), Quasi‐experimental studies (or controlled clinical trial without true randomization).	Feasibility and acceptability only studies, without IPV outcomes Pre–posttest studies with a single group design Non‐clinical study (e.g., secondary data study, reviews, editorials) Uncontrolled clinical study Qualitative studies Prospective and retrospective observational studies
Publication type	English language studies Published, peer‐reviewed full‐text articles	Non‐English publications Non‐peer‐reviewed

##### Exclusion criteria

We did not exclude studies based on intervention setting, country of study, or regionality, such as rural versus urban localities. Comparator/control groups of eligible studies could have been in the form of usual care, wait list controls, placebo, or alternative control formats. No limitations were placed on studies based on their duration of follow‐up. In addition, studies with repeated measures or before‐after outcomes (without a control group) were not included. Non‐IPV studies were excluded, where they focused on child abuse, peer violence, acquaintance rape, elder abuse, bullying, parental violence, and other non‐dating victimization. Studies were also excluded where the extent of technology use was limited to participant randomization, recruitment, screening, and referral outcome only (e.g., Klevens et al., 2012), follow‐up only. Finally, studies were excluded if they did not report changes in IPV victimization outcomes.

#### 4.1.2 Types of participants

Participants of diverse sexual orientations, racial/ethnic backgrounds, and ages were included who were experiencing or had experienced IPV. Furthermore, because IPV victimization is gender agnostic, studies comprising survivors of both genders who have experienced partner violence were included. However, most IPV interventions targeted women of reproductive age (14‐46 years) and emphasized secondary and tertiary prevention (i.e., intervention after violence had already occurred). Almost all the included studies primarily enrolled adult (>18) female‐identifying survivors. However, because only a few studies included male victims (primarily in couple dyads), these studies were not included if female victim data could not be acquired independently.

#### 4.1.3 Types of interventions

##### Experimental intervention

We considered all types of IPV digital interventions designed to reduce IPV exposure adverse mental health, and victimization outcomes. Operationally, we defined digital interventions as those deployed via mHealth, eHealth, and telehealth modalities, including personalized digital platforms (videos, smartphone apps, text messaging, chatbots, social media, and email). Digital interventions may be used for safety planning, digital consultation, referral‐to‐care, psychoeducation, and decision support, but must ultimately target mental health and victimization outcomes in each study. Interventions with digital‐only or digital plus traditional hybrid models were also included.

##### Control or comparator intervention

Control conditions varied across studies and included usual care (UC), intervention‐as‐usual (IAU), waitlist controls, or an active placebo control group format. Examples of control conditions included enhanced control groups, such as the control participants receiving modular IPV psychoeducation but not in a digital format, and participants receiving a smartphone app or website containing non‐IPV information (see Koziol‐McLain et al., 2018; Littleton et al., 2016), or face‐to‐face counseling (i.e., enhanced usual care) by a health care provider (Constantino et al., 2015).

#### 4.1.4 Types of outcome measures

Two categories of outcomes are of interest in this meta‐analysis: (1) mental health outcomes, and (2) victimization outcomes (see Primary outcomes).

##### Primary outcomes

The primary outcomes of interest to be quantitatively synthesized were:

1. IPV‐related common mental health disorders:

a. *Depression*, or any self‐reported or clinical base change in depressive symptoms.
b. *PTSD*, or any self‐reported or clinical base change in PTSD symptoms.
c. *Anxiety*, or any self‐reported or clinical base change in anxiety symptoms.

2. Any changes in *IPV victimization* outcomes (i.e., physical aggression, sexual violence, psychological abuse).

##### Secondary outcomes

Secondary outcomes were not meta‐analyzed here. However, commonly reported secondary outcomes in retrieved RCTs were (1) self‐efficacy (or ability to create and use a safety plan based on intra‐person, demographics, and extra‐person variables), (2) risk awareness (or objective awareness of present or future IPV risk), (3) decisional conflict (ability to navigate an abusive context, stay or leave the abusive relationship), and (4) safety planning (e.g., executing a personalized safety plan).

### 4.2 Search methods for identification of studies

An initial set of articles were retrieved and used to create a library of Medical Subject Headings (MeSH) terms using an online search building tool called Yale MeSH Analyzer (see Grossetta & Wang, 2018). The MeSH term analysis grid was generated to help establish indexing consistency. We combined key search terms with MeSH in two broad classifications: *“digital interventions”* AND *“intimate partner violence”* (see search strategy in Table 2). All search terms were restricted or expanded using suitable Boolean and proximity operators. Filters and limiters included “English” and “year of publication.” We used controlled vocabulary to account for variant spellings, truncations, and wildcards. Database‐specific searches were done using aggregated or simplified keywords (free‐text and subject headings; see Supporting Information: Appendix 1).

**Table 2:** Search strategies in databases

In traditional and grey databases			
	Technology		Intimate partner violence
Search terms	(mHealth OR mobile apps OR eHealth OR mobile applications OR interactive mobile application OR apps OR “mobile app” OR electronic interventions OR safety app OR decision aid OR mobile intervention OR smartphone OR smartphone apps OR smartphone‐based app OR smartphone‐delivered OR mobile‐delivered OR technology‐mediated OR internet‐based OR web‐based OR computer‐based OR computerized OR electronic OR technology‐based OR “use of technology” OR m‐Health OR e‐Health OR social network OR social media OR mobile phone OR mobile device OR Virtual communit* OR virtual reality OR Twitter OR Facebook OR WhatsApp OR WeChat “mobile health” OR “mobile care” OR “m Health” OR “mobile phone” OR “mobile device” OR “mobile technology” OR “mobile communication” OR “mobile telecommunication” OR “mobile app” OR “mobile application” OR “mobile tool” OR “mobile messaging” OR “mobile electronic device” OR “mobile telephone” OR “mobile phones” OR “mobile devices” OR “mobile technologies” OR “mobile communications” OR “mobile telecommunications” OR “mobile apps” OR “mobile applications” OR “mobile tools” OR “mobile messages” OR “mobile electronic devices” OR “mobile telephones” OR “mobile intervention” OR “mobile interventions” OR “mobile delivered” OR “mobile delivery OR information, communication technology OR ICT OR email)	AND	(“Intimate Partner Violence”[Mesh] OR “partner violence” OR “partner abuse” OR “dating violence” OR “dating abuse” OR dating violence OR partner abuse OR adolescent dating violence OR OR stalking OR assault OR coercion OR “digital abuse” OR rape OR battered women OR “domestic abuse” OR “wife abuse” OR “Spouse Abuse”[Mesh] OR “Domestic Violence”[Mesh:noexp] OR intimate partner violence[tiab] OR domestic violence[tiab] OR dating violence[tiab] OR partner violence[tiab] OR domestic abuse[tiab] OR partner abuse[tiab] OR (Abuse[tiab] OR abusive[tiab] OR abused[tiab] OR battered[tiab] OR battering[tiab] OR violent[tiab] OR violence[tiab] OR assaultive[tiab])

#### 4.2.1 Electronic searches

We searched the following databases with the support of a health science research librarian:

1. Traditional broad‐spectrum databases:
  a. PubMed Central (PMC)
  b. Web of Science
  c. Cumulative Index of Nursing and Allied Health Literature (CINAHL) Plus,
  d. PsychINFO.
2. Clinical trial registry (i.e., ClinicalTrials.gov).
3. Grey literature search on Google Scholar for conference abstracts, as well as organizational and government repositories.
4. Reference searching of all included studies for further relevant studies.

All study searches were conducted from March to April 2019 (14 studies found) and February to March 2021 (two studies added). During peer review, a final RCT was recommended by the reviewers for addition, bringing the total number of included RCTs to 17.

#### 4.2.2 Searching other resources

Topic‐related organizational websites (e.g., www.VAWnet.org) were searched to reduce possible publication bias by avoiding unpublished studies, as well as purposive searches of key authors predominantly involved in mHealth‐linked IPV interventions and their concurrent publications on this topic.

1. In addition, searches were conducted by target‐searching recent reviews of IPV interventions for digital interventions.
2. Lastly, author known to research and publish on tech‐based IPV interventions were searched by name for possible inclusion.

### 4.3 Data collection and analysis

#### 4.3.1 Description of methods used in primary research

All included RCTs had to have a well‐defined control group. All studies included IPV as a principal outcome or secondary outcome. However, some studies addressed IPV in relation to other conditions, such as anger management, relationship distress (Braithwaite & Fincham, 2009, 2011, 2014), HIV drug adherence, substance use (Gilbert et al., 2016), trauma distress, as well as somatic co‐morbidities. Typically, studies testing a technology‐based intervention for IPV added a qualitative component to explore survivor's lived experiences and to understand the feasibility and acceptability of IPV digital interventions (e.g., Alhusen et al., 2015; Debnam & Kumodzi, 2019; Ford‐Gilboe et al., 2020; Lindsay et al., 2013; Tarzia et al., 2017). For example, Bacchus et al., (2016) used nested interpretive methods to study perinatal home visitors' and women's experiences with IPV screening and receiving intervention in the form of mHealth technology (i.e., a computer or tablet) or a home visitor‐led method. Qualitative findings indicate that IPV survivors consider digital interventions an acceptable response and support tool (Hegarty et al., 2019).

#### 4.3.2 Selection of studies

Two reviewers (including an experienced dating violence researcher) and a health science research librarian independently screened for titles and abstracts from a random selection of yielded articles. An inter‐rater agreement of 80% (range of 0–1) (i.e., *κ* statistics, Landis & Koch, 1977) was established for ongoing study selection and risk of bias assessments.

#### 4.3.3 Data extraction and management

Two team members extracted data from each article into a standardized worksheet that included information on study details, as well as primary and secondary outcomes data (e.g., means, SD, CIs, odds ratio [OR]). We also extracted study descriptors (e.g., assignment and blinding protocol, digital intervention used, study duration), control or comparison group descriptors, and fidelity outcomes (attrition and follow‐up data). This information has been itemized in the Summary of findings Table 3. Authors CE and JC reached consensus on what studies should be included based on preset criteria. Although we first ran our analyses using Comprehensive Meta‐Analysis (CMA; Borenstein et al., 2011), we used Cochrane's RevMan to run a final analysis, finding convergence between both software.

**Table 3:** Characteristics of studies included in meta‐analysis

Study author (year)	Country	RCT Design	Underlying theory and mechanism of action	Participants and study setting	Description of intervention and control conditions	Intervention delivery mode
Ford‐Gilboe et al. (2020)	Canada; 3 provinces (British Columbia, Ontario, New Brunswick)	Two‐arm double‐blind randomized controlled trial RCT. 1:1 allocation	Principles of trauma‐ and violence‐informed care (TVIC)	462 Canadian adult women	**Intervention**: Tailored, interactive online safety and health intervention (iCAN Plan 4 Safety). **Control**: A static, non‐tailored version of this tool.	Website/Online intervention, reminder messages
Decker et al. (2020)	Nairobi, Kenya (informal settlements)	Randomized, controlled, participant‐blinded superiority trial (1:1 ratio)	Social cognitive theory, empowerment and trauma‐informed care, emphasizing safety and empowerment through agency in decision‐making and healing.	Women at risk of and experiencing IPV in Nairobi, Kenya. 352 were consented and enrolled at baseline (86.48%; *n* = 175 control, *n* = 177 intervention	**Intervention:** The myPlan Kenya app sections included the following: the Healthy Relationships, my Relationship, Red Flags, and My Safety section using the validated Danger Assessment scale. Other sections were the My Priorities section, My Plan section provided the tailored safety plan based on data supplied by the user in the previous sections. **Control**: Standard “usual care” set of referrals to IPV‐related legal, health, safety, counseling and financial resources—delivered in an app‐like webpage with research staff assistance.	App
Glass et al. (2017)	United States: Arizona, Maryland, Missouri, and Oregon	Multistate, community‐based longitudinal RCT with one‐to‐one allocation ratio and blocked randomization. Computerized blocked randomization provided intrastate stratification and for participants with children (aged ˂18 years) at home, ensuring each state's groups remained relatively balanced. The randomization sequence (concealed from research assistants [RAs])	Dutton's empowerment model and resiliency models.	Currently abused Spanish‐ or English‐speaking women (*N* = 720).	**Intervention**: Tailored, Internet‐based safety decision aid (included priority‐setting activities, risk assessment, and tailored feedback and safety plans). **Control**: A control website offered typical safety information available online	Website
Hegarty et al. (2019)	Australia	Two‐group pragmatic randomized controlled trial, randomly assigned (1:1) by computer to receive either the intervention or control website. As the initial portion of the website containing the baseline questions was identical for both groups, there was no way for women to tell which group they had been allocated to. Women were masked to treatment allocation, although it is possible that some may have guessed which website they were receiving. All the research team was masked to participant allocation until after analysis of the 12‐month data.	Psychosocial Readiness Model, and Contemplation Ladder	Women aged 16–50 years currently residing in Australia. 422 eligible participants were randomly allocated to the intervention group (227 patients) or control group (195 patients)	**Intervention**: An online healthy relationship tool/website and safety decision aid for women experiencing intimate partner violence (I‐DECIDE): RCT. On the intervention website, participants were presented with three modules addressing healthy relationships, safety, and priorities. **Control**: Static intimate partner violence information website	Website
Glass et al. (2021)	United States: Oregon and Maryland	An automated algorithm randomly assigned enrolled participants to the intervention or control group. The randomization was stratified on the state of residence, having children (child/no child in the home), and type of college/university.	Dutton's empowerment model	Three hundred forty‐six women (175 intervention, 171 control) from 41 colleges/universities in Oregon and Maryland. English‐speaking women (including cisgender and transgender women),	**Intervention**: The myPlan is an interactive decision aid and safety planning intervention that is free and accessible via a mobile app and website (myPlanApp.org), appropriate technology for college students (Glass et al., 2015, 2017). myPlan was created drawing upon foundational work in empowerment (Dutton, 1992), a previous trial of an internet safety decision aid for women of all ages (Glass et al., 2017), and the literature on safety planning (Campbell & Glass, 2009; Campbell et al., 2001; Davies & Lyon, 1998; Hardesty & Campbell, 2004; McFarlane et al., 2002, 2004). **Control**: A usual safety planning website guided by basic emergency safety planning information provided to students on‐campus and found through IPV websites targeting young women in unsafe relationships.	App/Website
Koziol‐McLain et al. (2018)	New Zealand	Web‐based two‐arm parallel randomized controlled trial (RCT): password‐protected intervention website (safety prioritized, danger assessment, and action plan modules) or control website (standard, non‐individualized). Computer‐generated randomization was based on a minimization scheme with stratification by the severity of violence and children	Dutton's empowerment model	New Zealand women (*n* = 412; 27% Māori) who had experienced IPV in the past 6 months	**Intervention**: Interactive Web‐based safety decision aid (iSafe)—password‐protected intervention website (safety priority setting, danger assessment, and tailored action plan components) **Control**: website (standard, non‐individualized information). Women randomly assigned to the control group were able to access a standardized list of resources and emergency safety plans throughout the 1‐year postbaseline follow‐up via a password‐protected trial website.	Web‐based safety decision aid
Braithwaite and Fincham (2009)	Large US public university	RCT, ePREP (*n* = 38) versus computer‐based placebo intervention (*n* = 39). A computer‐generated randomization list was used to assign participants to either the ePREP (*n* = 38) or the placebo/control intervention (*n* = 39).	Prevention and Relationship Enhancement Program (PREP, Markman, Stanley, & Blumberg, 2001) centering on communication skills and relational theory.	77 college students in romantic relationships of 4 months or longer. Only one partner of a dyadic partnership in intervention. 71% female. White, 76%; African American, 10%; Hispanic, 7% and “Other,” 7%. Over 9% reported living together and 91% living apart. Ages range (from 18 to 25), average age = 19.4 at the beginning of the study.	**Intervention**: One hour‐long ePREP. Based on the Prevention and Relationship Enhancement program (PREP, Markman, Stanley, & Blumberg, 2001), The focus of the intervention is skills training in effective communication techniques and problem‐solving skills. It also teaches individuals how to enhance positive aspects of romantic relationships. **Sections of the ePREP Intervention**; Improving Your Relationship; Filters; Communication; Issues and Events; Problem solving; Fun, Friendship and The Foundation of a Good Relationship; Ground Rules. **Control**: Placebo presentation and material that provided inert descriptive information about anxiety, depression, and relationship distress such as definitions, prevalence rates, and available forms of treatment.	Computer/Email
Stevens et al. (2015)	United States	RCT randomly assigned to an experimental condition, Assignment to condition was based on a computer‐generated random number table. RA blinded to study condition. One hundred twenty‐nine participants were randomly assigned to the intervention condition (TSS), and 124 participants were randomly assigned to the control condition (EUC).	Motivational Interviewing (MI)	Three hundred women (ages 18 years and above), reporting past‐year IPV visiting a pediatric emergency department. The sample was roughly half African American and half Caucasian American. Considering that 80% of the children had Medicaid as their insurer	**Intervention**: Telephone support services (TSS)—assessment phase, implementation phase, monitoring phase, secondary implementation phase, and termination phase. **Control**: Enhanced usual care (EUC)— Includingn phone calls about the child's recent visit to the ED and following up on any non‐IPV injury concerns endorsed by the woman. However, no discussions about IPV or community resources.	Telephone
Tiwari et al. (2010)	Hong Kong, China	Assessor‐blinded randomized controlled trial (RCT). Participants were randomized (1:1) to the intervention or control group according to a list of random permutations prepared by computer‐generated blocked randomization performed by a research staff member who had not been involved in participant recruitment. The block size was kept secure by the randomizer, and the order of allocation was centrally controlled to avoid any bias in selection. The allocation sequence was concealed in opaque envelopes. At the time of randomization, the research assistant who had successfully recruited a participant called the site investigator, who then opened the envelope containing the group assignment. To ensure random assignment, no detail was provided to the site investigator about the identity of the participant. Assessors were not involved in the design of the study, did not know the study hypotheses, and were blinded to group assignment.	Dutton's empowerment model and Cohen's Social Support Theory; and telephone social support	Telephone intervention to improve the mental health of community‐dwelling women abused by their intimate partners: a randomized controlled trial 18 years or older with a history of IPV. were randomly assigned to the intervention (*n* = 100) or control (*n* = 100) group	**Intervention**: 12‐week advocacy intervention, Telephone intervention. The intervention used for this study is classified as a less intensive advocacy intervention (an intervention of not more than 12 total hours). The empowerment component + social support component. **Empowerment support**: 30 min to deliver, was provided once in a oneone‐to‐one‐to‐one interview conducted in a private room (in the center or one of the outreach sites) by a designated research assistant at the beginning of the 12‐week intervention. At the end of the interview, each of the women was given an empowerment pamphlet to reinforce the information provided. **Social support**: 12 scheduled weekly telephone calls (initiated by the designated research assistant) and 24‐h access to a hotline for the study participants for additional social support. **Control**: Community services referral.	Phone Calls
Braithwaite and Fincham (2011)	Large US public university	RCT, ePREP (*n* = 40) versus computer‐based placebo intervention (*n* = 37). Using computer‐generated randomization list	Prevention and Relationship Enhancement Program (PREP, Markman, Stanley, & Blumberg, 2001) centering on communication skills and relational theory.	77 couples (152 individuals). The average age was 19.92 years. Average relationship length (between 1 and 2 years). 20% reported current cohabitation. Overall, 77% White (non‐Hispanic), 10% Latino, 8% Black, 3% “Mixed Race” and 2% Asian. Only one of a dyadic partnership in intervention. Only those who had been in a committed romantic relationship for 6 months or longer were invited to participate. average relationship length was between 1 and 2 years and 20% of participants reported that they were currently cohabiting.	**Intervention**: Computer‐based preventive intervention (ePREP) versus active placebo control group. ePREP condition taught empirically based methods for improving romantic relationships. **Control**: inert information about anxiety, depression, and relationships such as definitions, prevalence rates, and available forms of treatment—detailed in Braithwaite and Fincham (2009)	Computer/Email APIM was used to test the impact of ePREP relative to the placebo intervention (see Figure 1). The omnibus test of distinguishability (I‐SAT) was used to test for empirical distinguishability (Olsen & Kenny, 2006) and revealed that all of the variables examined were distinguishable by gender with the exception of alternatives monitoring, constructive communication, and self‐reported physical assault.
Littleton et al. (2016)	US, students at one of four universities and community colleges	RCT, interactive program (*n* = 46) or a psycho‐educational self‐help website (*n* = 41)	Cognitive‐behavioral therapy	Eighty‐seven college women with rape‐related PTSD were randomized to complete the interactive program (*n* = 46) or a psycho‐educational self‐help website (*n* = 41).	**Intervention**: From Survivor to Thriver program, an interactive, online therapist‐facilitated cognitive‐behavioral program for rape‐related PTSD. The From Survivor to Thriver Program consisted of nine program modules. The program was designed to be completed sequentially, with participants completing one module at a time. **Control**: Psycho‐educational self‐help website (*n* = 41). with content of the first three modules of the interactive program including the symptoms of PTSD, information about relaxation and grounding, and information about healthy coping strategies. No multimedia content (videos from the program developers, audio recorded relaxation exercises) or any interactive exercises.	Website
Braithwaite and Fincham (2007)	Large public university in the US Northeast	Three‐arm RCT. Participants were randomly assigned to take part in one of the three interventions.	Prevention and Relationship Enhancement Program (PREP, Markman, Stanley, & Blumberg, 2001) centering on communication skills and relational theory.	Ninety–one young adult in dating relationships. Women made up 59% of the sample. Ethnic background was distributed as follows, Caucasian, 60.9%; Asian, 18.7%; African American, 5.5%; and “Other”, 14.3%.	**Intervention**: Computer‐based relationship‐focused preventive intervention (ePREP) relative to depression and anxiety‐focused computer–based preventive intervention. individually administered computer‐based presentations (comprising written text and pictures, no audio or video material was used), the pace of which was controlled by the participant. For the interventions, examples of how to employ certain skills were provided in vignettes that described couples utilizing the skill in question. In each case, the intervention included a quiz following the presentation of each section of material and this quiz assessed participants' mastery of the information presented. The interventions were intentionally balanced to contain approximately the same amount of content and therefore took approximately the same amount of time to administer. **CBASP** Participants in the depression and anxiety‐focused intervention condition received training in an empirically validated method for reducing symptoms of depression and anxiety. This intervention, based on the Cognitive Behavioral Analysis System of Psychotherapy (CBASP) developed by McCullough (2000), teaches techniques for analyzing and changing patterns of maladaptive thinking and behavior. **Control**: Presentation and worked through material with information about anxiety, depression, and relationship information such as definitions, prevalence rates, and available forms of treatment.	Computer/Email
Doss et al. (2016)	United States, national sample	Using a random number generator, 151 couples were randomized into the web‐based intervention condition and 149 couples were randomized into the waitlist control condition.	Integrative Behavioral Couple Therapy (IBCT; Christensen et al., 2010)	300 heterosexual couples (*N* = 600 participants) participated; couples were generally representative of the US in terms of race, ethnicity, and education. Individuals were primarily White, non‐Hispanicnon‐Hispanic (67.2%), African American (17.2%), or White, Hispanic (10.2%), with smaller numbers of Asian/Pacific Islander (3.3%), American Indian/Alaska Native (0.7%), and Biracial/Other (1.4%) participants.	**Intervention**: In the program, couples' complete online activities and have four, 15‐min calls with project staff. **Control**: TwoTwo‐month‐month wait‐list control group.	Website
Constantino et al. (2015)	United States, Pittsburg, several sites	RCT, three‐arm: Online (ONL), Face‐to‐Face (FTF), and Waitlist Control (WC). A sequential, transformative mixed‐methods design was used. Participants were randomly assigned to one of three study groups by permuted block randomization. The designation of numbers for intervention conditions (i.e., 1 = ONL, 2 = FTF, and 3 = WLC) were concealed from data collectors and the statistician	Advocacy lens,	32 adult female participants who were 45.2% Asian, 32.3% White, and 22.5% Black	**Intervention**: HELPP (Health, Education on Safety, and Legal Support and Resources in IPV Participant Preferred) intervention among IPV survivors. Online (ONL) HELPP. The ONL intervention consisted of six modules delivered by e‐mail once a week for 6 weeks. Face‐to‐Face (FTF) HELPP. The FTF intervention consisted of six modules and was given to each participant once a week for 6 weeks. Six HELPP modules, 1 week at a time (Constantino et al., 2014) to each participant (i.e., by e‐mail for ONL and in‐person for FTF).	Computer/Email
Zlotnick et al. (2019)	United States	Two‐group, randomized controlled trial.	Motivational Interviewing (MI), Acceptance & Commitment Therapy (ACT)	53 currently pregnant or within 6‐months postpartum women seeking mental health treatment. Of the 53 women enrolled in the randomized trial, 17 women (32%) identified themselves as Hispanic/Latina. Six (11%) identified themselves as Black or African American, 27 (51%) as White, 5 (9%) as Bi‐racial or Multi‐ethnic, and 15 (28%) as Other.	**Intervention**: A brief, motivational computer‐based intervention, SURE (Strength for U in Relationship Empowerment), for perinatal women with IPV seeking mental health treatment. SURE is a computerized intervention (30–40 min) delivered on a small tablet computer and was adapted and delivered using an intervention software platform, Computerized Intervention Authoring Software (CIAS, Interva, Inc.; Ondersma et al., 2005). **Control**: Participants interacted with the computer software and were guided by the same narrator. Control condition included watching brief segments of popular television shows and following up with questions for ratings of their preference. No follow‐up booster session for the control condition. Participants in both conditions received information on IPV and community resources for IPV.	Small tablet computer
Gilbert et al. (2016)	United States, New York City	RCT, A study investigator randomly assigned groups of 4 to 9 women to 1 of 3 study conditions; a computer‐generated randomization algorithm was designed to balance the number of women per study arm via an adaptive, biased‐coin procedure. Investigators were masked to treatment assignment until the final 12‐month follow‐up assessment was completed in April 2013.	The intervention was informed by social cognitive learning theory, which focuses on observation, modeling, and skill rehearsal through role‐play and feedback from group members. Empowerment theory also guided a strengths‐based approach of WORTH to build collective efficacy of women to negotiate safe relationships and counter the stigma that they face as women in community corrections	191 substance‐using women in probation and community court. A total of 103 participants were assigned to Computerized WORTH, 101 to Traditional WORTH, and 102 to Wellness Promotion. Over (68%) identified as Black or African American, and 47 (15.4%) identified as Latina. TwoTwo‐thirds‐thirds (66.0%) were single and never married. 25 women (8.2%) were employed, and 278 (90.8%) had ever been in prison or jail. Of the women, 194 (63.4%) reported using illicit drugs in the past 90 days. About one quarter (*n* = 81; 26.5%) tested positive for an STI, and 43 (14.1%) tested positive for HIV.	**Intervention**: Computerized, group‐based HIV and IPV intervention among substance‐using women mandated to community corrections in the Women on the Road to Health (WORTH) intervention. Three study arms: (1) 4 group sessions intervention with computerized self‐paced IPV prevention modules (Computerized Women on the Road to Health [WORTH]), (2) traditional HIV and IPV prevention group covering the same content as Computerized WORTH without computers (Traditional WORTH), and (3) a Wellness Promotion control group. **Control**: **The Wellness Promotion** control arm comprised of 4 weekly 90‐min group sessions on maintaining a healthy diet, increasing fitness in daily routines, addressing tobacco use, learning stress‐reduction methods including guided meditation, and creating and achieving personal health goals. Wellness Promotion did not address IPV prevention.	Computer
Braithwaite and Fincham (2014)	United States, Tallahassee, Florida	RCT, ePREP (*n* = 26 couples) versus computer‐based placebo intervention (*n* = 25 couples).	Prevention and Relationship Enhancement Program (PREP, Markman, Stanley, & Blumberg, 2001) centering on communication skills and relational theory.	A community sample of 52 married couples (21% Black, 3% Asian, 65% White, 7% Latino, 4% Mixed/biracial) who had been married, on average, 4.3 years	**Intervention**: Computer‐based (text and video) preventive intervention (ePREP) versus active placebo control group. ePREP adapted and computerized version of the Prevention and Relationship Enhancement Program (PREP, Markman et al., 2010). Intervention and control presentation lasted approximately 1 h, followed by weekly homework assignments for the next 6 weeks (1 h per session, completed as a couple). Posttreatment assessment at 96% retention rate. The computer‐presentation portions of both interventions were self‐paced and included both text and video. **Control**: Participants viewed a presentation that taught inert information that was designed to seem like part of an intervention	Computer

Study author (year)	Analysis and timing of posttreatment assessment	Outcome measures	Study duration and follow‐up	Overall retention and attrition	Total mean age (SD)	Main findings
Ford‐Gilboe et al. (2020)	Intent to treat protocols; Generalized Estimating Equations were used to test for differences in outcomes by study arm. Differential effects of the tailored intervention for 4 strata of women were examined using effect sizes. Exit survey process evaluation data were analyzed using descriptive statistics, *t*‐tests, and conventional content analysis.	Primary (depressive symptoms, PTSD symptoms) and secondary (helpfulness of safety actions, confidence in safety planning, mastery, social support, experiences of coercive control, and decisional conflict)	Baseline and 3, 6, and 12 months later via online surveys	Retention was 89.6, 87.0, and 87.0% at 3, 6, and 12 months, respectively for the tailored group. In the non‐tailored group, retention was 91.8, 91.3, and 90.5% at 3, 6, and 12 months, respectively. Attrition across all time points was small and largely due to losing contact with women.	Mean age = 34.61 (10.7)	Women in both tailored and non‐tailored groups improved over time on primary outcomes of depression (*p* < 0.001) and PTSD (*p* < 0.001) and on all secondary outcomes. Changes over time did not differ by study arm. Women in both groups reported high levels of benefit, safety, and accessibility of the online interventions, with low risk of harm, although those completing the tailored intervention were more positive about fit and helpfulness. Importantly, the tailored intervention had greater positive effects for 4 groups of women, those: with children under 18 living at home; reporting more severe violence; living in medium‐sized and large urban centers, and not living with a partner.
Decker et al. (2020)	Intent‐to‐treat, differences‐in‐differences approach used random effects logistic and linear regression models	Primary outcomes were safety preparedness, safety behavior and IPV; secondary outcomes include resilience, mental health, service utilization, and self‐blame.	Baseline, 3‐month follow‐up	88% retention	Mean age of 26.52 years old (4.70)	At 3 months, intervention participants reported increased helpfulness of safety strategies used relative to control participants (*p* = 0.004); IPV was reduced in both groups. Among women reporting the highest level of IPV severity, intervention participants had a significant increase in resilience (*p* < 0.01) compared with controls, and significantly decreased risk for lethal violence (*p* < 0.01).
Glass et al. (2017)	Intent‐to‐treat approach including generalized estimating equations to test for differences in change over time between groups. Analyses used a Gaussian distribution with an identity link function, with an exchangeable working correlation matrix. Time had three levels (baseline, 6 months, 12 months) for IPV severity, and mental health outcomes had four levels (baseline pre‐test, baseline post‐test, 6 months, and 12 months) for decisional conflict and two levels (baseline, and 6þ12 months) for safety behaviors. In addition, *χ* ^2^ analyses tested group differences in the number of women who left the abuser by 12 months. For all women, independent sample t‐tests tested differences in safety behaviors and IPV between women who left (*n* = 390) and stayed (*n* = 283).	Decisional conflict, safety behaviors, and repeat IPV; secondary: depression and PTSD.	Baseline, 6 months, and 12 months	Missing data for intervention and control groups (accounting for attrition and incomplete responses) was 9.0% and 5.3% (6 months) and 8.5% and 9.2% (12 months), respectively.	Mean age = 33.41 (10.64)	At 12 months, no significant group differences in IPV, depression, or PTSD. Intervention women reported significantly less decisional conflict after one use (*Î*² = −2.68, *p* = 0.042) and greater increase in safety behaviors they rated as helpful from baseline to 12 months (12% vs. 9%, *p* = 0.033) and were more likely to have left the abuser (63% vs. 53%, *p* = 0.008). Women who left had higher baseline risk (14.9 vs. 13.1, *p* = 0.003) found more of the safety behaviors they tried helpful (61.1% vs. 47.5%, *p* < 0.001), and had greater reductions in psychological IPV (11.69 vs. 7.5, *p* = 0.001) and sexual IPV (2.41 vs. 1.25, *p* = 0.001) than women who stayed.
Hegarty et al. (2019)	Both the main and imputed data analyses were done according to intention‐to‐treat principles.	Self‐efficacy, depression, fear of partner, and number of helpful behaviors for safety and wellbeing, and cost‐effectiveness	Baseline, posttest, 6 months, 12 months.	179 (79%) participants in the intervention group and 156 (80%) participants in the control group completed a 12‐month follow‐up.	Mean age = 33.7 years (8.48)	Mean self‐efficacy at 6 months and 12 months was lower for participants in the intervention group than for participants in the control group, although this did not meet the prespecified mean difference (6 months: 27.5 [SD: 5.1] vs. 28.1 [4.4], imputed mean difference 1.3 [95% CI: 0.3 to 2.3]; 12 months: 27·8 [SD: 5.4] vs. 29·0 [5.0], imputed mean difference 1.6 [95% CI: 0.5 to 2.7]). We found no difference between groups in depressive symptoms at 6 months or 12 months (6 months: 22.5 [SD: 17.1] vs. 24.2 [17.2], imputed mean difference –0.3 [95% CI: –3.5 to 3.0]; 12 months: 21.9 [SD: 19.3] vs. 21·5 [19.3], imputed mean difference –1.9 [95% CI: –5.6 to 1.7]). Qualitative findings indicated that participants found the intervention supportive and a motivation for action.
Glass et al. (2021)	Intention to treat analysis.	Four measure of intimate partner violence (IPV; Composite Abuse Scale [CAS], TBI‐related IPV, digital abuse, reproductive coercion [RC]) and depression. Safety behavior, decisional conflict, past‐week depression and suicide risk, substance misuse, and preparation for decision‐making	Baseline, 6 and 12 months	Six‐month retention was 98.3% for both the intervention (*N* = 174) and control (*N* = 173) groups. Twelve‐month retention was 98.9% for the intervention group (*N* = 175) and 96.6% (*N* = 171) for the control group.	Mean age in CTRL = 20.61 (2.12) Mean age in INT = 21.23 (2.56)	Reduction in RC and improvement in suicide risk were significantly greater in the myPlan group relative to controls (*p* = 0.019 and *p* = 0.46, respectively). Increases in the percent of safety behaviors tried that were helpful significantly reduced CAS scores, indicating a reduction in IPV over time in the myPlan group compared to controls (*p* = 0.006). Findings support the feasibility and importance of technology‐based IPV safety planning for college women. myPlan achieved a number of its objectives related to safety planning and decision‐making, the use of helpful safety behaviors, mental health, and reductions in some forms of IPV.
Koziol‐McLain et al. (2018)	Analyses were by intention to treat.	Primary endpoints were self‐reported mental health (Center for Epidemiologic Studies Depression Scale‐Revised, CESD‐R) and IPV exposure (Severity of Violence Against Women Scale, SVAWS) at 12‐month follow‐up.	baseline, then 3, 6, and 12 months	Retention at 12 months was 87%	Median age 29.0 (16–59)	The adjusted estimated intervention effect for SVAWS was −12.44 (95% CI: −23.35 to −1.54) for Māori and 0.76 (95% CI: −5.57 to 7.09) for non‐Māori. The adjusted intervention effect for CESD‐R was −7.75 (95% CI: −15.57 to 0.07) for Māori and 1.36 (−3.16 to 5.88) for non‐Māori. No study‐related adverse events were reported. Conclusions: The interactive, individualized Web‐based iSafe decision aid was effective in reducing IPV exposure limited to indigenous Māori women. The discovery of a treatment effect in a population group that experiences significant health disparities is a welcome, important finding.
Braithwaite and Fincham (2009)	Per protocol, completers‐only	Symptoms of depression and anxiety, IPV, communication patterns, and relationship satisfaction.	Baseline, 8 weeks, 10 months (44 weeks).		Mean age of 19.4 years.	At 10‐month follow‐up, participants in the ePREP condition experienced improved mental health and relationship‐relevant outcomes for almost all outcome measures. Participants seemed to get worse before they get better. Follow‐up: baseline, 8 weeks post‐baseline, and 10 months (44 weeks) post‐baseline. Positive effect of ePREP on IPV was not significantly weakened if partner terminated relationship and began new one
Stevens et al. (2015)	Per protocol, completers‐only	IPV victimization, feelings of chronic vulnerability to the perpetrator, depressive symptoms, and PTSD	Baseline, 3, 6 months.	There was a 76% retention rate for the intervention group from baseline to 3 months (*n* = 98), and a 77% retention rate for the control group from baseline to 3 months (*n* = 95). There was a 70% retention rate for the intervention group at 6 months (*n* = 90), and a 72% retention rate for the control group at 6 months (*n* = 93).	Mean age in INT; 28.8 (8.3), Mean age in CTRL: 29.5 (8.5).	TSS and EUC groups did not differ on any outcome variable,
Tiwari et al. (2010)	There were no missing values or dropouts, and the analysis was consistent with the intention‐to‐treat principle.	Change in depressive symptoms (between baseline and 9 months). Secondary outcomes: changes in IPV, health‐related quality of life, and perceived social support (between baseline and 9 months). The usefulness of the intervention was evaluated at 9 months.	baseline and the 3‐ and 9‐month (i.e., 6 months postintervention) follow‐up	No subjects were lost to follow up.	Mean age in INT 38.18 ± 7.61. Mean age in CTRL 37.99 ± 6.79	An advocacy intervention showed Intervention effects at 3 and 9 months were not significantly different (*p* = 0.86). The intervention significantly reduced depressive symptoms by 2.66 (95% CI, 0.26 to 5.06; *p* = 0.03) versus the control, less than the 5‐unit minimal clinically important difference and psychological aggression as well as improving perceived social support and safety promoting behavior for at least 6 months following the intervention. Although only usual community services were provided to the women in the control group, there was an improvement in their depression. It is probable that just abuse screening by itself may have a beneficial effect for abused women.2 There is inconsistent evidence as to whether advocacy intervention improves social support in the short term for abused women who have actively sought help.
Braithwaite and Fincham (2011)	intent to treat analysis all participants were included in the analyses regardless of whether they completed the 6 weeks of the intervention and full information maximum likelihood (FIML) estimation was used to impute missing value.	Commitment attitudes, patterns of communication, prior 6 weeks psychological aggression and physical assault, relationship satisfaction and latent variables, depression latent variables, anxiety latent variable.	Baseline and 6‐weeks FU	INT: ~88% retained, CTRL: ~92% retained	Mean age = 19.92 years (1.58)	Compared to placebo intervention, ePREP participants demonstrated improved mental health and relationship functioning at a 6‐week follow‐up. Using the actor‐partner interdependence model, we found that, compared to those who received a placebo intervention, ePREP participants demonstrated better mental health and relationship functioning at a 6‐week follow‐up. Those who engaged more fully in the intervention and mastered the communication techniques generally experienced superior outcomes. Implications of and recommendations for computer‐based dissemination are discussed.
Littleton et al. (2016)	Intent‐to‐Treat (ITT) model.	Rape‐related PTSD symptoms, working alliance with their therapist	Baseline, 3 months follow‐up	Among the 38 participants who initiated the interactive program, 11 (28.9%) completed at least part of phase one (modules 1–3) of the program, 13 (34.2%) completed at least part of phase 2 (modules 4–5), 11 (28.9%) completed at least part of phase 3 (modules 6–9), and two (5.3%) did not complete any program modules. Six participants (15.8%) completed the entire program.	Mean age = 22 years old (range 18–42 years)	Both programs led to large reductions in interview‐assessed PTSD at post‐treatment (interactive *d* = 2.22, psycho‐educational *d* = 1.10), which were maintained at 3‐month follow‐up. Both also led to medium‐ to large‐sized reductions in self‐reported depressive and general anxiety symptoms. Follow‐up analyses supported that the therapist‐facilitated interactive program led to superior outcomes among those with higher pre‐treatment PTSD whereas the psycho‐educational self‐help website led to superior outcomes for individuals with lower pre‐treatment PTSD. Future research should examine the efficacy and effectiveness of online interventions for rape‐related PTSD including whether treatment intensity matching could be utilized to maximize outcomes and therapist resource efficiency
Braithwaite and Fincham (2007)	NA	Depression, anxiety, IPV (CTS–Negotiation, CTS–Psychological, CTS–Physical), quality of their relationship, Constructive Communication, Trust, Global Relationship Quality	Baseline, and at an 8‐week follow up		NA	Participants in the ePREP and CBASP interventions experienced significantly reduced symptoms of depression and anxiety and significant improvements in relationship relevant variables relative to controls. The outcomes from the two treatment conditions did not significantly differ from one another. These findings suggest that computer–based preventive interventions may be a viable and efficacious means for preventing depression, anxiety, and relationship distress.
Doss et al. (2016)	Intention to Treat	Nine primary outcome measures (assessing four relationships and five individual functioning constructs). **Relationship outcomes**: relationship satisfaction, Positive Relationship, and Negative Relationship Quality, Relationship Confidence. **Individual outcomes**: Depression, Anxiety, Perceived Health, Work Functioning, Quality of Life	Baseline, 2, 5, 7, 8 weeks.	Of the 151 couples randomly assigned to complete the intervention, 129 couples (86%) completed the entire program. An additional eight couples (5%) completed the program through the “Understand” phase, the point where we hypothesized couples would receive a therapeutic dose of the intervention even if they did not complete the entire program.	Mean age = 36.11 (9.58)	Compared to the waitlist group, intervention couples reported significant improvements in relationship satisfaction (Cohen's *d* = 0.69), relationship confidence (*d* = 0.47), and negative relationship quality (*d* = 0.57). Additionally, couples reported significant improvements in multiple domains of individual functioning, especially when individuals began the program with difficulties in that domain: depressive (*d* = 0.71) and anxious symptoms (*d* = 0.94), perceived health (*d* = 0.51), work functioning (*d* = 0.57), and quality of life (*d* = 0.44).
Constantino et al. (2015)	No ITT, completers‐only. Patient demographics and outcome measures were reported using (1) means and standard deviations for continuous variables and (2) frequency distribution for categorical variables. OneOne‐way‐way ANOVA and *χ* ^2^‐tests were used to compare the differences of demographics among the three groups. The differences between baseline and post‐intervention within each group were compared using a paired Student's *t*‐test. The change from baseline to post‐intervention was also computed and compared between the three groups using oneone‐way‐way ANOVA with post hoc multiple comparisons. All statistical analyses were conducted using SAS version 9.3 (SAS Institute), and significance was set at *p* < 0.05.	Outcome measures were anxiety, depression, anger, personal, and social support	Baseline and 6 weeks post‐intervention	Almost 100% retention after randomization	Mean age: 40.0 years	The HELPP intervention (1) decreased anxiety, depression, anger, and (2) increased personal and social support in the ONL group. The HELPP information and intervention was shown to be feasible, acceptable, and effective among IPV survivors compared with participants in the WLC group. The WLC participants displayed (1) increased levels of anxiety, depression, and anger and (2) decreased levels of personal and social support, post‐intervention. Participants who received the HELPP intervention, whether online or face‐to‐face, reported a decrease in feelings of anxiety, depression, and anger, and an increase in personal support and social support, with significant improvements in the online group.
Zlotnick et al. (2019)	No ITT, completers‐only	IPV outcomes (Severe combined abuse, physical, emotional abuse, harassment), Feasibility and acceptability of the intervention	Baseline and 4‐month follow‐up assessment.	Of the 28 women randomized to the intervention condition, 26 (93%) completed a booster session. Forty‐nine women (92%) completed the follow‐up assessment. Of the 28 women randomized to the intervention condition, 26 (93%) completed a follow‐up session. Of the 25 women randomized to the control condition, 23 (92%) completed a follow‐up session.	Mean age of 28 years old (5.3; range 18–37 years)	All participants (100%) found the information and resources in SURE to be helpful. Our preliminary results found the degree of IPV decreased significantly from baseline to the 4‐month follow‐up for the SURE condition (paired *t*‐test, *p* < .001), while the control group was essentially unchanged. Moreover, there was a significant reduction in emotional abuse for SURE participants (*p* = 0.023) relative to participants in the control condition. There were also reductions in physical abuse although non‐significant (*p* = 0.060). All participants (100%) found the information and resources presented in SURE to be helpful. These high satisfaction scores are also reflected in our high completion rate of the intervention and booster session.
Gilbert et al. (2016)	Consistent with the intent‐to‐treat approach, we estimated intervention effects by analyzing participant responses based on their experimental assignment.	Physical, injurious, and sexual IPV victimization in the previous 6 months at 12‐month follow‐up	IPV outcomes were assessed only at baseline, 6‐ and 12‐month follow‐ups	The 87% or higher retention rate at each follow‐up did not differ significantly by condition	Mean age was 41.5 years (10.5)	Computerized WORTH participants reported a significantly lower risk of physical IPV victimization, severe injurious IPV victimization, and severe sexual IPV victimization at 12‐month follow‐up when compared with control participants. No significant differences were seen between Traditional WORTH and control participants for any IPV outcomes.
Braithwaite and Fincham (2014)	Actor Partner Interdependence Model with treatment effects to analyze the obtained dyadic data. Intention‐to‐treat analysis. Full information maximum likelihood (FIML) estimation was used to accommodate missing data.	Self and partner minor and severe forms of psychological aggression and physical aggression.	baseline (Time 1), 6 weeks post‐baseline (Time 2), and 1‐year post‐baseline (Time 3).	All participants completed the presentation phase of the intervention (the initial lab session) and retention rates were 96% at post‐treatment, and 92% at the 1‐year follow‐up	Mean age 32.36 years (10.01)	ePREP reduced physical and psychological aggression. An average of 90% reduction in physical aggression, and a 0.18 standard deviation reduction in psychological aggression) maintained up to 1‐year follow‐up (92% retention)

Abbreviations: APIM, Actor Partner Interdependence Model; CTRL, control group; C‐CBASP, computer‐based cognitive‐behavioral analysis system of psychotherapy; DV, domestic violence; ePREP, Prevention and Relationship Enhancement program; I‐ABBT, Internet‐delivered acceptance‐based behavior therapy; I‐ACT, Internet‐delivered acceptance and commitment therapy; I‐BA, Internet‐delivered behavioral activation; I‐CFT, Internet‐delivered compassion‐focused therapy; I‐CMT, Internet‐delivered compassionate mind training; I‐E and MBI, Internet‐delivered exposure and mindfulness‐based intervention; I‐MBCT, Internet‐delivered mindfulness‐based cognitive therapy; INT, intervention groups; IPV, intimate partner violence; NS, not stated; PTSD, post‐traumatic stress disorder; RCT, randomized control trial; S‐ACT, smartphone‐based acceptance and commitment therapy; S‐MBCT, smartphone‐based mindfulness‐based cognitive therapy; SBIRT, screening, brief intervention, and referral to treatment; S/I, selective/indicated; T, treatment; U, universal; WORTH, Women on the Road to Health.

#### 4.3.4 Assessment of risk of bias in included studies

**Figure 1:**
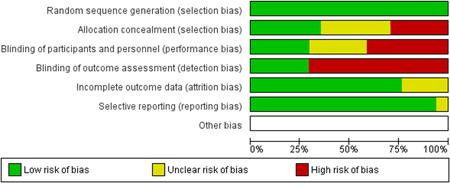
Risk of bias breakdown

We assessed the methodological quality of each study using the Cochrane risk‐of‐bias tool (RoB 2) for randomized trials on RevMan (see Figure 1). This tool employs a predefined range of six bias‐detecting domains, concentrating on aspects of trial design, study procedures, and study reporting as follows:

1. Random sequence generation (selection bias)
2. Allocation concealment (selection bias)
3. Blinding of participants and personnel (performance bias): All outcomes
4. Blinding of outcome assessment (detection bias): All outcomes
5. Incomplete outcome data addressed (attrition bias): All outcomes
6. Selective reporting (when null or negative results are not fully or accurately reported to suppress undesirable findings).

Overall risk‐of‐bias judgment was rated as follows:

1. A “low risk” of bias if the study is judged to be at *low risk* of bias for all domains.
2. An “unclear risk” study is judged to *raise some concerns* in at least one domain but not at *high risk* of bias for any domain.
3. A “high risk” study is judged to be at *high risk* of bias in at least one domain in a way that substantially lowers confidence in the result.

This risk of bias analysis served as a rubric to guide our interpretation of results (Higgins et al., 2008, 2016; Sterne et al., 2019) and was not used to decide on what studies to include in this meta‐analysis as recommended by Higgins et al. (2011).

#### 4.3.5 Measures of treatment effect

##### Continuous data

Effect estimate for continuous outcomes was quantified as the standardized mean difference (SMD) or Cohen's *d*. The SMD was expressed in standard deviation units to account for adjusted and unadjusted means and divergent outcomes measures. Further, SMD was a more suitable statistic than the mean difference (MD) since outcome measures varied across studies (Higgins et al., 2016). Relevant data to calculate average effect sizes were extracted from study trials (i.e., means, sample sizes, SD, CI, OR, SEs, and *p* values). Given the small number of studies for each considered outcome, a fixed‐effects model (FEM) for continuous data was used as a computational model as programmed into RevMan 5.4.1—this way, we present a more parsimonious pooled effect size.

Gilbert et al. (2016) was the only study reporting OR using random‐effects logistic regression models of IPV victimization outcomes as a calculation of effect size (along with 95% CIs) to compare the risk of physical, sexual, and IPV frequency/occurrence in the treatment groups versus the control group. As a result, appropriate transformations were used to convert effect sizes to a standard metric on RevMan to re‐express ORs as SMDs.

##### Dependent effect sizes

Dependent effect sizes occur for several reasons (Van den Noortgate et al., 2013). To avoid biasing the overall effect size, we did not include pre‐and post‐test studies in our analysis. Four studies were excluded (Draucker et al., 2019; Fiorillo et al., 2017; Gray et al., 2015; Hassija & Gray, 2011). For example, we used the most conceptually practical intervention group means and standard deviation in studies with three arms to avoid effect size dependency. For example, the study by Constantino et al. (2015) used a three‐arm RCT with two treatment conditions (face‐2‐face vs. online multi‐component intervention) compared to a waitlist control. In this case, we picked the online arm to compare with a split control to create a single pair‐wise comparison (Higgins et al., 2011). Four studies had *three* treatment arms. To avoid unit of analysis errors, we selected one treatment arm and compared this with a split control group to create a pair‐wise meta‐analysis. We chose the arm that most closely reflected a technology‐based intervention. For example, Constantino et al. (2015) compared an online intervention with their face‐to‐face version of the HELPP intervention (Health, Education on Safety, and Legal Support and Resources in IPV Participant Preferred). Braithwaite and Fincham (2007) compared a computer‐based relationship‐focused preventive intervention (called ePREP) to depression‐ and anxiety‐focused computer–based preventive intervention (CBASP). Gilbert et al. (2016) compared a Wellness Promotion control group to a computerized, group‐based HIV and IPV intervention compared to a control group of substance‐using women mandated to community corrections in the Women on the Road to Health (WORTH) intervention.

Other instances of effect size dependence, such as multiple outcomes measured by the same set of participants, outcomes measured at various follow‐up times, and multiple correlations from a common sample, were addressed using analytical methods as recommended by the Cochrane Handbook (Higgins et al., 2011). An effect size of 0.2 was considered a small effect, 0.5 a moderate effect, and 0.8 a large effect, as recommended by Cohen (1988).

*How we handled dependent findings*:

1. Multiple published studies on the same group of participants were treated as one to minimize duplicated data bias and overestimating the intervention effect.
2. Only the most comprehensive data was used if multiple publications for the same data were sourced.
3. We considered multiple time points of outcomes measures (3, 6, and 12 months).
4. We combined or excluded some intervention groups where needed to create a single pair‐wise comparison (e.g., in studies with three, two, or more treatment groups).
5. We did not base our determination of study independence on authoring activities. (i.e., for example, the same author(s) across studies) as long as each study was distinct.

##### Timing of data collection

Not all RCTs conducted follow‐ups at the same time points. Therefore, we elected to run our meta‐analysis only where we had at least four RCTs for each *Outcome by Time* comparison as follows:

1. Three time points for *depression* (0–3 months, 3–9 months, and 10+ months),
2. One timepoint for *anxiety* (0–3 months),
3. One for *PTSD* (3–6 months),
4. Two for *physical victimization* (0–6 months and > 6 months),
5. Two for *psychological victimization* (0–6 months and >6 months), and
6. One for *sexual victimization* (6–9 months only).

#### 4.3.6 Unit of analysis issues

Participants were randomly allotted to intervention or control groups, and participants were analyzed as individuals. However, some studies used couple dyads as units of analysis (e.g., Braithwaite & Fincham, 2009, 2011, 2014). These studies offered gender‐separated data, allowing us to extract only the information pertinent to female survivors. In addition, no statistical corrections were made for unit‐of‐analysis concerns to prevent overestimation of the intervention effects (e.g., CIs or standard errors that were too small) (see Pigott, 2012; Thomas et al., 2003).

#### 4.3.7 Dealing with missing data

Study authors were contacted to obtain relevant data where data was unclear or missing to calculate the effect sizes. Two authors were reached for this reason, and both responded with the information needed (Braithwaite & Fincham, 2011; Gilbert et al., 2016). Therefore, we did not need to employ any data imputation approaches to account for missing data, such as setting Cohen's *d* to zero or the *p*‐value to 0.05 or using a population SD generated from the literature to account for imputation with uncertainty (Higgins et al., 2011).

#### 4.3.8 Assessment of heterogeneity

##### Statistical heterogeneity

Statistical heterogeneity is inevitable in meta‐analyses (Higgins et al., 2011). Therefore, a test for statistical heterogeneity was conducted alongside a narrative review of heterogeneity. A fixed‐effects meta‐analysis (FEM) was utilized given the small number of studies analyzed and to achieve statistical parsimony even though we found varying true effects across studies reflective of the “real‐world” circumstances of IPV survivors (DerSimonian, 1986; Lipsey & Wilson, 2001).

##### The extent of heterogeneity (Higgins *I*²)

The degree of heterogeneity was also calculated using the Higgins' *I²* statistic (ranges from 0% to 100%). A higher percentage indicates higher variation across studies (Deeks et al., 2008). *I*² did not depend on the number of studies, and where we found high heterogeneity, we removed outliers studies with extreme results (i.e., extremely small or large effects). Studies were identified as outliers when their 95% CI fell outside the 95% CI of the pooled effect. Using the “leave‐one‐out” method, we assessed the influence of extreme case RCTs (e.g., studies with over positive or negative effect sizes were removed). We observed the impact of including or removing these extreme case studies on the extent of heterogeneity, characterized as low (0%–40%), moderate (30%–60%), substantial (50%–90%), or considerable (75%–100%) (Higgins & Thompson, 2002).

##### Confirmation of statistical heterogeneity (Cochran's *Q*)

We used the standard *χ* ^2^ test (Cochran's *Q*‐statistic) to test the null hypothesis that effects across studies were the same (Hedges & Olkin, 1985). Cochran's *Q*‐statistic reflects divergences between each study's effect against the pooled mean effect. We report the *p*‐value for this *χ* ^2^ test using a recommended *p* < 0.01 significance level due to this test's low power (Sutton et al., 2000). However, since the number of studies included was less than 20, Cochran's *Q*‐statistic is to be interpreted with a caveat.

##### Visual evaluation of heterogeneity

Forest plots were used to visualize the extent of heterogeneity and inconsistencies across studies and the significance of the pooled effect (Song, 1999). The sources and degree of heterogeneity were handled as follows: (1) using a fixed‐effects model, (2) by reporting a summary estimate of a location parameter (e.g., summary SMD estimate), and a variability parameter (i.e., summary 95% CI).

#### 4.3.9 Assessment of reporting biases

Given the overall small number of studies for each outcome included in this meta‐analysis, we could not empirically evaluate the influence of publication bias for this meta‐analysis.

#### 4.3.10 Data synthesis

Cochrane's RevMan (version 5.4.1) was used for all meta‐analyses. Study effect sizes were synthesized using a fixed‐effect model, as suggested by Borenstein et al. (2009); given the small number of studies in our analyses. SMD effect sizes (Cohen's *d*, ESs), calculated as the mean of the treatment group minus the mean of the control group divided by the pooled standard deviation, were calculated for eligible studies and weighted by the inverse of the variance to adjust for sample size and bias (Borenstein et al.,  2009). A Cohen's *d* of 1 indicates the two groups differ by one standard deviation, and so on. A Cohen's *d of* 0.2 to 0.5 is considered a “small” effect size, 0.5 to 0.8 represents a “medium” effect size and >0.8 represents a “large” effect size. For this meta‐analysis, a negative effect size favored the intervention group (i.e., meaning digital interventions led to a reduction in depression, anxiety, PTSD, and victimization outcomes).

#### 4.3.11 Subgroup analysis and investigation of heterogeneity

We could not conduct sample characteristic moderator analyses due to the small number of studies. Heterogeneity was assessed using *I* ^2^ and *χ* ^2^ measures of heterogeneity (Cochran's *Q* test; 95% CIs), and forest plots helped us visualize the effect size distribution across each outcome. A minimum of four studies was retained per sub‐group after adjusting for heterogeneity and removing outliers.

#### 4.3.12 Sensitivity analysis

We explored the impact of procedural decisions (e.g., using fixed‐ vs. random‐effects models) to compare the effects of outlier studies on the overall effect sizes. In addition, we removed outliers in a post hoc analysis by excluding RCTs assessed to be outliers based on their CI relative to the pooled CI and based on a high *I* ^2.^ Finally, we reported our results with and without these outliers to give a clear picture of effect sizes.

##### Treatment of qualitative research

Qualitative studies, cross‐sectional studies, and case reports were excluded as they do not lend themselves to robust synthesized meta‐analysis as was the intent of this review.

#### 4.3.13 Summary of findings and assessment of the certainty of the evidence

We assessed five risks of bias domains independently (see Risk of bias in included studies). Disagreements in assigning risk of bias were resolved by discussion. Each domain was assigned a low, medium, or high risk of bias (see Figure 1). For this meta‐analysis, several measures were applied to limit the risk of bias. For example, we only included studies with a well‐defined control group, studies using an objective measure of IPV mental health and victimization outcomes, and studies using elements of the RoB 2 risk assessment domains (e.g., allocation concealment, subject blinding, etc.) in our estimation of study quality. The risk of bias assessments, on the other hand, had no bearing on which RCT we included or excluded.

## 5 RESULTS

### 5.1 Description of studies

Seventeen RCTs were meta‐analyzed, each with well‐defined control groups. Overall, 4590 survivors were sampled, with the mean survivor age spanning from 19 years in Braithwaite and Fincham (2009) to 41.5 years in Gilbert et al. (2016). Retention rates across studies were high but varied depending on intervention delivery modules (lower with didactic content) and study duration (lowest attrition at 3 and 6 months post‐intervention). Baseline, 3‐, and 6‐month follow‐ups were typical data collecting timepoints; however, timepoints varied between investigations. For example, Braithwaite and Fincham (2009) collected data at baseline, 8 weeks, 10 months (44 weeks). Braithwaite and Fincham (2011) collated their data at baseline and 6 weeks follow‐up, and Doss et al. (2016) at baseline, 2, 5, 7, and 8 weeks—to mention a few. Baseline data collection was primarily done in person. Some studies conducted data collection activities using digital or remote methods (e.g., email, anonymous chat, phone calls). In other studies, participants completed surveys and intervention activities on a study‐related website, handheld tablet (e.g., Zlotnick et al., 2019), or a smartphone. Studies often considered the participant's device preference, device ownership, and safety from their abusers in using this type of remote data collection (e.g., Hegarty et al., 2019). Overall, eligibility for most studies required participants to have access to a safe phone or computer, reliable Internet, and a secure email address and phone number.

Participants' safety was of the utmost importance, as numerous techniques were implemented to ensure they felt protected and empowered. Safety strategies included using a safe phone number, safe contact information, alternate contact person(s), safe, designated times for study check‐in, proxy services to track down participants, and non‐descript language or codewords when advertising the study and during calls related to the study to ensure no one was listening in.

Most research teams used Data Safety Monitoring Boards (DSMB) to track adverse effects, data management, and study safety issues. Some studies reported harm or adverse effects during their trial. For example, Zlotnick et al. (2019) stated “a total of 22 serious adverse events were classified as unrelated to the study.” As standard practice and where safe, participants were directed to local trusted providers in situations where IPV risk was high, urgent, and potentially lethal (e.g., Tiwari et al., 2010), including local and national crisis hotline numbers, or asked to notify the study team. Hegarty et al. (2019); described abusive spouses' negative and positive responses upon learning that their victims were participating in the study. However, this did not result in any differences between groups at 6 or 12 months, and no adverse events or harms were explicitly attributed to digital interventions.

### 5.2 Results of the search

**Figure 2:**
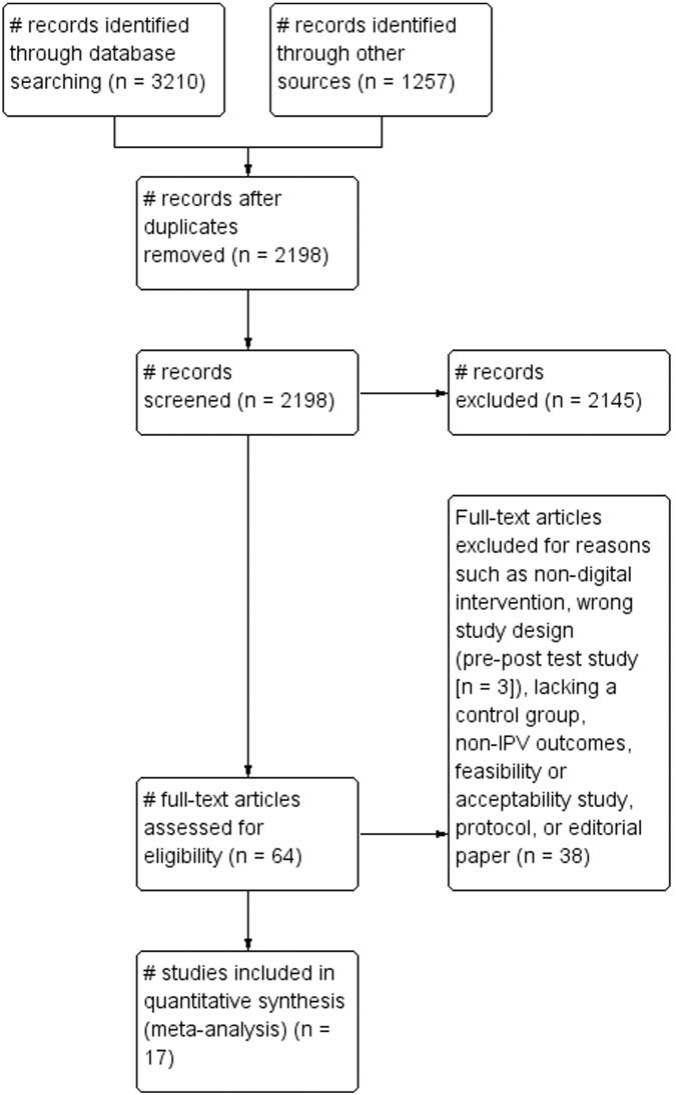
Search and screen diagram

We identified 3210 studies in traditional databases and 1257 studies from grey literature and hand searches, from which 2198 studies were deemed duplicates and removed. Following title and abstract screening of the remaining 64 studies, 17 RCTs met the inclusion criteria and were retained (see Figure 2). The remaining 47 full‐text articles were excluded for reasons spaning non‐use of digital interventions and a lack of a control group, or for being a feasibility or acceptability study only (e.g., Klevens et al., 2012; Ranney et al., 2013). Others were at the protocol phase when writing this review (e.g., Ford‐Gilboe et al., 2020; Glass et al., 2017; Koziol‐McLain et al., 2016; Sabri et al., 2019). Other studies fell outside the review period (e.g., McFarlane et al., 2002). Four single group pre‐post studies were excluded (Draucker et al., 2019; Fiorillo et al., 2017; Gray et al., 2015; Hassija & Gray, 2011). Non‐IPV studies were excluded where they focused on child abuse, peer violence, acquaintance rape, elder abuse, bullying, parental violence, and other non‐dating victimization. Finally, studies were excluded where the extent of technology use was limited to participant randomization (e.g., using a computer algorithm), recruitment, screening, consent signing, and referral only or follow‐up only (e.g., Klevens et al., 2012). We have presented the study flow diagram in Figure 2.

### 5.3 Included studies

#### 5.3.1 Sample characteristics

Most of the included RCTs were published in 2016 (*n* = 5 or 32%). Study sample sizes ranged from 15 to 672 participants (*N* = 4590, all females) from diverse settings. Due to the low number of men in RCTs, we omitted male victims from our analysis. Survivors' age ranged from 19 to 41.5 years. In most studies, samples were predominantly White (>60%), except in Constantino et al. (2015) (45.2% Asian survivors) and Gilbert et al. (2016) (67% Black survivors).

Given this lack of diversity, RCTs like Koziol‐McLain et al. (2018) focused on uniquely marginalized survivors (i.e., Māori women), while Tiwari et al. (2010) focused on a community sample of Chinese women, and Glass et al. (2017) targeted Spanish‐speaking women. Gilbert et al. (2016) also focused on substance‐using women in community corrections. As an eligibility criterion, several RCTs required survivors to no longer reside with their abusers (e.g., Constantino et al., 2015)—living with an abuser may affect therapeutic efficacy compared to other survivors. While some RCTs required participants to be separated from the perpetrator with a protection order from the court before participating in the study.

Since not enough RCTs evaluated this precondition, we did not examine subgroup differences (i.e., intervention effect based on whether the survivor was living vs. not living with an abusive partner). Other RCTs eliminated participants with competing psychotherapies, active suicidality, acute distress, and substance addictions. Almost all studies focused on heterosexual couples, except (Glass et al., 2021) with cisgender and transgender women. Most RCTs focused on married or cohabiting couples and dyads, not in couple's therapy (e.g., Braithwaite & Fincham, 2014).

#### 5.3.2 Intervention characteristics

##### Study location

Twelve of the 17 RCTs included were conducted in the United States. In addition, Ford‐Gilboe et al. (2020); and Hegarty et al. (2019); conducted studies in Canada and Australia, respectively. Other studies were conducted in New Zealand (Koziol‐McLain et al., 2018), Nairobi, Kenya (Decker et al., 2020), and Hong Kong, China (Tiwari et al., 2010). Some studies were conducted across multiple states in the US, spanning Arizona, Maryland, Missouri, and Oregon (Glass et al., 2017). Ford‐Gilboe et al. (2020) conducted their study in three Canadian provinces (British Columbia, Ontario, and New Brunswick). Overall, retained RCTs were conducted across diverse settings (community samples, clinics, college campuses).

##### Study setting

Participants were often recruited from several settings. For example, some trials offered digital intervention or usual care services at a community health center, childcare center, clinical and wellness center, or recreational facility (see Tiwari et al., 2010). In addition, some studies leveraged high‐traffic spaces, such as large public universities (Braithwaite & Fincham, 2009; Braithwaite & Fincham, 2011; Braithwaite & Fincham, 2014), pediatric emergency departments (Stevens et al., 2015), and online (Koziol‐McLain et al., 2018), or using social media platforms (Hegarty et al., 2019). Glass et al. (2017) used multi‐strategy approaches, combining online, community‐based settings, health clinics, and other community locations (e.g., college campuses, women's bathrooms in coffee shops). In comparison, Littleton et al. (2016) used ads on four campuses (e.g., fliers, bus ads, campus bulletin), departmental websites, and social media (e.g., Facebook).

In most cases, recruitment materials did not explicitly indicate that the research was an IPV‐focused study or that IPV experiences were a prerequisite for participation in trial studies. For example, some trials described their study as a “woman's health study.” This was done to prevent associating the study with domestic violence prevention, as this may cue abusers to the program's intent. Instead, this information was provided as attenuated messages on websites or during the initial enrollment and consent process. In addition, some studies described their trials as a “self‐help” program or as a “marriage counseling alternative” (e.g., see Constantino et al., 2015; Doss et al., 2016). Glass et al. (2017) used Spanish‐language recruitment materials in targeted ads on Spanish‐language radio, newspapers, and community organizations.

##### Who facilitated the interventions?

Nurse practitioners and physicians facilitated most trials (see Glass et al., 2017, 2021; Hegarty et al., 2019). In addition, trained research assistants, registered nurses, clinicians (Glass et al., 2017, 2021; Hegarty et al., 2019), social workers (Tiwari et al., 2010), and psychology graduate students (Braithwaite & Fincham, 2009, 2011, 2014) helped deliver the intervention. Study facilitators were also coached on accurate data collection, study adherence, ethical research, cultural competency, therapeutic alliance, empowerment, and advocacy to provide survivor‐centered social support in a nonjudgmental and trauma‐informed manner.

##### What did interventions contain?

Several of the RCTs mentioned here provided survivors with services outside of the research study, as well as preliminary psychoeducation on toxic relationships and signs of abuse. The myPlan app inspired or was adapted for most of this information (Glass et al., 2010). Some interventions used pragmatic tools to help survivors make decisions, such as the Danger Assessment tool (Campbell et al., 2009) and some form of a ‘priority setting’ feature that focused on safety/risk issues, the health and well‐being of their child(ren), the survivor's health and well‐being, access to services, and feelings for a spouse. Finally, some studies used pairwise comparisons to provide feedback on survivors' priorities for safety in abusive relationships (see Glass et al., 2017, 2021; Hegarty et al., 2019; Koziol‐McLain et al., 2018). Fiorillo et al. (2017) delivered content on “psychoeducation on IPV trauma, willingness and acceptance, mindfulness, defusion, and self‐as‐context, clarifying values, and committed action consistent with values” via web‐based multimedia sessions (p. 106). A thorough action plan of strategies and resources for resolving their safety and health concerns was generally provided at the end of each intervention.

Intervention content could be modular or module‐based content (Littleton et al., 2016) or delivered as weekly or one‐time quizzes, video vignettes, videoconference, and homework assignments (Braithwaite & Fincham, 2011), or an “avatar with a female voice that addressed the participant by name, and narrated for the program aloud, allowing low‐literacy participants to participate” (see Zlotnick et al., 2019, p. 316). For example, Fiorillo et al., 2017 employed a mix of fictitious case studies, video vignettes, psychoeducation, exercises, and worksheets in their web‐based intervention.

##### How were interventions administered?

Most interventions were delivered by computer or “online.” Some RCTs used apps (Decker et al., 2020; Glass et al., 2021) or web‐based safety decision aid (Hegarty et al., 2019; Koziol‐McLain et al., 2018), or a website/online intervention with SMS reminder messages (Ford‐Gilboe et al., 2020), or website/online intervention with emails (Braithwaite & Fincham, 2007, 2009, 2011; Constantino et al., 2015). Zlotnick et al. (2019) delivered their intervention on small tablet computers, followed by telephone for a booster session. Interventions delivered via telephone modalities described using telephone social support (or TSS; see Stevens et al., 2015; Tiwari et al., 2010). Others had a videoconference or asynchronous chat feature (Doss et al., 2016). Some RCTs reported using hybrid modalities, which combined face‐to‐face or in‐person interventions with digital modalities in the same intervention (see Braithwaite & Fincham, 2011; Littleton et al., 2016; Tiwari et al., 2010). Due to financial constraints, Stevens et al. (2015) supplied some participants with free mobile phones to interact with research personnel. As an added incentive to charge and carry the mobile phone, these individuals received 200 free minutes each month to contact family and friends for the 6 months the research lasted. However, they had to discontinue this phone distribution since participants regularly exceeded their allowed minutes or handed phones to relatives.

##### Theoretical foundations of IPV digital interventions

While the underlying theories across anti‐IPV interventions are explicit, the mechanism of action (i.e., theory of change) showing causal linkages for improving survivors” mental health appears less so. It is worth noting that the theoretical frameworks used in IPV digital intervention design varied across studies. However, since some of these proprietary apps were based on the myPlan app, most were theoretically grounded in Dutton's empowerment model (Dutton et al., 1999). Studies like Glass et al. (2017, 2021); Koziol‐McLain et al. (2018); and Hegarty et al. (2019) also used a strength‐based empowerment‐focused approach to improve survivors' autonomy, protection, and empowerment. These interventions considered power and control dynamics, gender disparities, device access, and other disparities in their design (Warshaw et al., 2013). Others aimed to improve survivors' and their loved ones' protection and safety by reducing decisional conflicts; and then reducing exposure to IPV victimization (Glass et al., 2017).

Other interventions (see Stevens et al., 2015; Zlotnick et al., 2019) used Motivational Interviewing (MI) to determine survivor priorities regarding immediate and long‐term needs and *reflective listening* to center the survivor's needs, voice, and agency. Tiwari et al. (2010) used Dutton's empowerment model and Cohen's Social Support Theory to create an advocacy‐based intervention. Fiorillo et al. (2017) based their intervention on Acceptance and Commitment Therapy (ACT; Hayes et al., 2006, 2019), a third‐wave cognitive‐behavioral therapy (CBT). Doss et al. (2016) based their intervention on the Integrative Behavioral Couple Therapy (IBCT; Christensen et al., 2010); to encourage emotional acceptance and behavior change (p. 6). Finally, Gilbert et al. (2016) used the social cognitive learning theory and the strengths‐based empowerment model to help substance‐using women in community corrections improve harm‐reduction, problem‐solving, and negotiation skills, as well as an understanding of IPV red flags, causes of unsafe sex, and substance use, safety planning, and social support service awareness.

#### 5.3.3 Description of proprietary IPV digital interventions

Several trials used proprietary interventions designed over several years like the iCAN Plan 4 Safety (Ford‐Gilboe et al., 2020), the myPlan app (Glass et al., 2017, 2021), I‐DECIDE (Hegarty et al., 2019), and iSafe (Koziol‐McLain et al., 2018). Two RCTs tested the same proprietary myPlan app intervention across two populations (Glass et al., 2017; Glass et al., 2021). Additionally, three other studies examined variants of the myPlan intervention (Decker et al., 2020; Hegarty et al., 2019; Koziol‐McLain et al., 2018). These proprietary interventions are described in the next section.

##### iCAN Plan 4 Safety (iCAN)

The Canadian‐based *iCAN Plan 4 Safety* (iCAN) was a personalized, immersive online safety and health intervention that adapted the US‐based myPlan app (Ford‐Gilboe et al., 2020). Priority‐setting focused on personalized input, risk evaluation, and action planning with intervention ingredients based on the same schema as the myPlan app, but with an explicit focus on principles of trauma‐ and violence‐informed care (TVIC; Ford‐Gilboe et al., 2020). Ford‐Gilboe et al., 2020 compared the customized, interactive iCAN intervention to a non‐tailored control version, finding that Canadian women who face barriers to support and still live with an abusive partner in both the treatment and control groups showed improvements in depression outcomes (*p* < 0.001) and PTSD (*p* < 0.001), as well as all secondary outcomes (helpfulness of safety actions, self‐efficacy, and mastery in safety planning, social support, experiences of coercive control, and decisional conflict). Changes over time were not different between research arms. Notably, the targeted intervention had a more significant positive impact on four unique categories of women: (1) those with children under the age of 18 living at home, (2) those who reported more extreme abuse, (3) those who lived in medium‐sized and large urban areas, and (4) those who did not live with an abusive partner.

##### myPlan intervention

From the earlier Internet Resource for Intervention and Safety (IRIS) project, Glass et al. (2010) developed the myPlan app as a digitalized safety decision aid intervention in two stages. The first phase of the study included a systematic review of current evidence on safety planning as well as a person‐ and community‐centered risk factors for IPV that influence women's safety planning self‐efficacy and intentions. Glass et al. (2010) then developed safety decision aid materials that focused on decision‐making conflicts and risk factors for lethal violence, using validated safety mechanisms. Finally, Glass et al. (2010) validated the intervention with input from experts, advocates, and survivors; (*phase one*) and tested it in *phase two* with ninety English‐ and Spanish‐speaking women in the US Pacific Northwest. Glass et al. (2010) discovered that after one use of a computerized safety and decision aid in a racially and ethnically diverse sample of women in the US‐Pacific Northwest, the automated safety and decision aid improved decision making and reduced decisional conflict (p. 1959).

The myPlan app (www.myplan.org) ‐ serves primarily as a decision aid to help survivors make informed decisions about their safety and well‐being. Its theoretical underpinning stems from the decisional conflict model using an empowerment approach to raise awareness of violence severity and create a safety plan (Dutton et al., 1999; Eden et al., 2015; Glass et al., 2017). In sum, the myPlan app (1) educates the survivor on relationship red flags; (2) estimates their level of danger (i.e., abuse severity) and risk for fatality using a weighted scoring protocol called the Danger Assessment (DA) tool with 20 dichotomous (yes or no) questions on the lethality of their relationships (Campbell, 2002, 2009); (3) estimates priorities for safety (e.g., child's welfare) using pairwise comparisons; (4) creates a checklist of tailored safety planning, (5) designs a tailored safety plan based on the survivor's level of danger and priorities; and ultimately (6) triages the victim‐survivor to trusted resources based on some consideration of their social ecology (Glass et al., 2010).

The myPlan app has been pilot tested with survivors (all females) in domestic violence (DV) shelters and support groups as a computerized version, hypothesizing improvements in the survivor's decisional conflict (Glass et al., 2010). Following a 12‐month follow‐up, the authors suggest the safety decisional aid reduced decisional conflict (*p* = 0.014, 95% CI) and increased the survivor's likelihood of creating a safety plan for themselves and their children with the option of leaving an abusive relationship if desired. Following this, several adaptions of the app have been tested with at‐risk groups, including Māori women (Koziol‐McLain et al., 2018), immigrant women (Sabri, 2018), LGBTQIA+ people, college women (Bloom et al., 2016; Littleton et al., 2016) and friends of survivors (Alhusen et al., 2015). Multi‐language versions are also available in Spanish and English (Eden et al., 2015; Glass et al., 2017) with several ongoing country‐specific adaptations and clinical trials.

##### iSafe intervention

The web‐based safety decision aid (*iSafe*), developed by Koziol‐McLain et al. (2018) is a password‐protected intervention website (with a safety priority setting, the Danger Assessment (DA, Campbell et al., 2009) or Danger Assessment‐Revised (DA‐R; for female same‐sex relationships), and tailored action plan components). The website was modified to allow both Māori and non‐Māori survivors access a safety priority setting activity and a five‐criteria priorities list adapted to the New Zealand context. The intervention help users create a tailored action plan linked to a matrix of resources. The iSafe study found that among a subgroup of Māori women only, there were reductions in IPV victimization at 6 and 12 months and depressive symptoms at 3 months only.

##### I‐DECIDE intervention

The I‐DECIDE intervention, like myPlan, was an interactive platform with interactive modules and a safety decision aid section. I‐DECIDE guided user's through a process of self‐reflection and self‐management based on the Psychosocial Readiness Model (Hegarty et al., 2019). This intervention derived elements from the Internet Resource for Intervention and Safety (IRIS) project (precedent to the myPlan app) and the iSafe project in New Zealand (Koziol‐McLain et al., 2018). On the intervention website, three modules of healthy relationships, safety, and goals (safety, well‐being, fitness, access to and availability of appropriate resources, and feelings for the partner and child well‐being—if applicable) were available to participants. To assess the participant's IPV motivation and ability to formulate and implement an action plan, a version of the “Contemplation Ladder”—a Stages of Change framework–and a motivational interviewing were used. Like iSafe and the myPlan app, an algorithm assisted the survivor in deciding on a top priority or immediate concern, followed up with an individualized action plan.

##### ePREP intervention

The ePREP intervention focused on relationship education and primary prevention of relationship conflict by targeting dynamic risk factors contributing to relationship distress—often with an emphasis on couple dyads as its users. By adapting the original PREP program developed by Stanley and Markman (1993), ePREP was developed by Braithwaite and Fincham (2007) for computerized administration in three RCTs reviewed here (Braithwaite & Fincham, 2007, 2009, 2011). In samples of college‐aged youth in dating relationships, the ePREP intervention focused on skill training in good communication strategies and problem‐solving skills. ePREP was compared to the Cognitive Behavioral Analysis System in Psychotherapy (CBASP; McCullough, 2000) in Braithwaite and Fincham (2007) with an 8‐week follow‐up. ePREP taught participants strategies for understanding and modifying maladaptive thought and behavior patterns. In addition, ePREP teaches people how to evaluate difficult circumstances in their lives and figure out why they did not get the desired results. The ePREP and CBASP were also compared to a control condition in which participants watched a presentation with descriptive details on anxiety, depression, and relationship dysfunction.

##### Other interventions

The effect of the ePREP intervention in an expanded 10‐month follow‐up study (Braithwaite & Fincham, 2009) demonstrating positive effects of ePREP on IPV and relationship skills gained, which were maintained if partners moved to start a new relationship. Braithwaite and Fincham (2011) administered ePREP to dating couples in a subsequent RCT. These ePREP studies were carried out for premarital dating/cohabiting relationships. Finally, Braithwaite and Fincham (2011, 2014) used the Actor Partner Interdependence Model (APIM) to compare ePREP to an active placebo control in a community sample of married couples, with treatment effects analyzed to obtain individualized and dyadic results.

### 5.4 Primary outcomes measures used

#### 5.4.1 Mental health

##### Depression

Validated instruments used to measure depression frequently included the Center for Epidemiological Studies Depression Scale (CES‐D; Radloff, 1977) or CESD‐R with 20 depressive symptoms reflecting DSM‐IV criteria for depression, with questions such as “*how often have you felt this way in the previous week or so?”* The entire CESD‐R score varied from 0 to 60 (clinical cutoff score of 16. Any score above 16 indicated clinically serious depression symptoms) (Shean & Baldwin, 2008). The CES‐D has a good correlation (*r* = .74). The Beck Depression Inventory (BDI) was another extensively used measure for depression. Due to the overlap in symptoms of depression and anxiety, the Inventory of Depression and Anxiety Symptoms (IDAS; see Braithwaite & Fincham, 2011) and the Depression, Anxiety, and Stress Scale (DASS; Lovibond & Lovibond, 1995) both examined depression and anxiety. Similarly, the 10‐item Panic, Social Anxiety and Trauma Scale (PANAS; Watson & Tellegen, 1988) measured positive and negative affect.

##### Anxiety

Anxiety was commonly measured using Beck Anxiety Inventory (BAI), the Generalized Anxiety Disorder (or GAD‐7; Spitzer et al., 2006), or the 9‐item self‐report Patient Health Questionnaire (PHQ‐9; Kroenke et al., 2001). The GAD‐7 showed good convergent validity relative to the Beck Anxiety Inventory (BAI; *r* = 0.72), the Depression Anxiety Stress Scale (DASS‐anxiety (*r* = 0.77), and the DASS‐stress scale (*r* = 0.79). Littleton et al. (2016) also used the 35‐item Four‐Dimensional Anxiety Scale (FDAS; Bystritsky et al., 1990) to measure past‐week physiological, cognitive, emotional, and behavioral anxiety symptoms.

##### PTSD

PTSD was measured with the 20‐item posttraumatic stress disorder (PTSD) Checklist for DSM‐VI (PCL‐5; Weathers et al., 2013) or the 17‐item PCL (Blanchard et al., 1996). Other instruments used were the 24‐item PTSD Symptom Scale‐Interview (PSS‐I; Foa et al., 1993), evaluating the frequency and intensity of PTSD symptoms in the past month. Psychometric assessments show these instruments demonstrate strong reliability and validity (Blevins et al., 2015).

##### IPV victimization outcomes

Physical, psychological, and sexual victimization outcomes were measured using self‐reports and validated measures, with whole or subscales used. These measures focused on the prevalence, frequency, and severity of violence. More commonly used were the Revised Conflict Tactics Scale (CTS–2) (Straus et al., 1996); the 30‐item Composite Abuse Scale (CAS; Hegarty, Bush, & Sheehan, 2005), or the 8‐item version of CAS (Hegarty & Valpied, 2007); the 8‐item Woman Abuse Screening Tool (WAST) (Brown et al., 2003); the 10‐item Women's Experiences with Battering scale (WEB; Smith et al., 1995); and the 46‐item Severity of Violence Against Women Scale (SVAWS; Marshall, 1992) (in Koziol‐McLain et al., 2018). The Danger Assessment (DA) or Danger Assessment—Revised (DA‐R; for same‐sex couples) was common for measuring lethality across studies. Several studies used the *Decisional Conflict Scale* to measure decisional conflict (O'Connor) and the *Safety Checklist* (Parker et al., 1999; Sullivan & Bybee, 1999) to gauge psychosocial domains of IPV. Culturally relevant measures such as the Chinese versions of the Revised Conflict Tactics Scales (C‐CTS2; Tiwari et al., 2007), Chinese Abuse Assessment Scale (C‐AAS; Tiwari et al., 2007), and Chinese version of the Beck's Depression Inventory‐II (C‐BDI‐II; Wang et al., 2011), were used in the study by Tiwari et al. (2010).

### 5.5 Secondary outcomes measures

Ancillary standardized or researcher‐generated survey measures were used to capture secondary outcomes such as client acceptability of the intervention and usability of the technology‐based interventions provided, often during process evaluation during studies.

#### 5.5.1 Relationship satisfaction and quality

Various instruments were used to measure relationship satisfaction on account of using an intervention, spanning the Communication Patterns Questionnaire (CPQ) to the 32‐item Dyadic Adjustment Scale (DAS; Spanier, 1976), the Couples Satisfaction Index (CSI; Funk & Rogge, 2007), and the Positive and Negative Relationship Quality (PNRQ; Fincham, & Rogge, 2010).

#### 5.5.2 Survivors' perception of digital interventions across studies

Several RCTs ended with process evaluations (e.g., exit interviews or surveys) to ascertain the survivor's opinion of the intervention used, specifically focusing on intervention usability, helpfulness, and acceptability (e.g., Doss et al., 2016; Ford‐Gilboe et al., 2020). Survivors rated these interventions with high acceptance. Many described them as beneficial for building a safety plan, making decisions, receiving support, being prepared to face abuse, taking action, and leaving an abusive relationship (Hegarty et al., 2019). In Ford‐Gilboe et al. (2020); the majority of women (96.0% in the tailored group and 93.8% in the non‐tailored group) agreed or strongly agreed that they “gained something from the intervention.” Over ninety percent of women indicated they “felt comfortable and safe” and said they “would recommend it to other women” (p. 11). However, about one‐quarter of participants reported feeling nervous or agitated while participating in the intervention (p. 11).

Some studies measured the acceptability of the digital intervention itself, including using the System Usability Scale (SUS; Brooke, 1996)—a 10‐item scale used to gauge the intervention hardware usability (e.g., ease of website navigation, satisfaction with the website). One study measured the therapeutic alliance between the participant and program facilitators using the Satisfaction with Therapy and Therapist Scale‐Revised (STTS‐R; Oei & Green, 2008) (also see Littleton et al., 2016). Other outcomes examined were perceived health, quality of life, work and relationship functioning, and positive and negative relationship quality.

#### 5.5.3 Control conditions

A majority of the studies had either a true control group given an attenuated version of the intervention. Others used wait‐list control groups (Constantino et al., 2015; Doss et al., 2016), treatment‐as‐usual, active placebo intervention (digitized vs. non‐digitized version of the same intervention), or an active control condition (e.g., Braithwaite & Fincham, 2014).

#### 5.5.4 Analytic approach

Most studies used baseline analyses to compare the differences between control and intervention groups, finding no difference between intervention groups at baseline. Some studies used generalized estimating equations (GEE) to test group differences across time (Ford‐Gilboe et al., 2020; Glass et al., 2021; Koziol‐McLain et al., 2018). In addition, other RCTs used one‐way ANOVA and chi‐square analyses to test group differences; and independent sample *t*‐tests to test differences in outcomes across groups (i.e., from baseline to post‐intervention). In addition, almost all studies utilized an intention‐to‐treat approach, including participants in the final analysis, whether or not they had accessed and completed the intervention at all follow‐up time points. Because of the small sample size in certain trials, RCTs such as Braithwaite and Fincham (2011) chose a less stringent alpha threshold of 0.10—all other studies used the conventional 0.05 alpha level. Finally, studies like Koziol‐McLain et al. (2018) combined an intention‐to‐treat approach with a per‐protocol analysis set to compare results output.

### 5.6 Excluded studies

In the final stage of the search, approximately 47 studies were excluded for a variety of reasons, including for being pre‐post studies with the same group, qualitative studies, studies with incorrect outcomes, feasibility‐only studies, systematic reviews and meta‐analyses, studies publishing on recruitment and engagement, and studies not in English. Of the 64 studies that were considered for final inclusion, 17 RCTs were chosen for meta‐analysis. The ‘Characteristics of excluded studies’ and the ‘Results of the search’ section itemizes some other reasons for excluding studies.

### 5.7 Risk of bias in included studies

Using Cochrane risk‐of‐bias tool (RoB 2) for randomized trials, each study was assigned a risk of bias score in six domains.

1. Four studies were rated ***high risk of bias*** (Braithwaite & Fincham, 2007; Braithwaite & Fincham, 2009; Braithwaite & Fincham, 2011; Zlotnick et al., 2019) due to violations in study allocation of concealment, reporting bias, and blinding. A study was deemed “high risk” if, in at least one domain, there was a significant risk of bias sufficient to substantially reduce confidence in the study's outcome.
2. Eleven studies were rated ***unclear risk of bias*** (Braithwaite & Fincham, 2014; Constantino et al., 2015; Decker et al., 2020; Doss et al., 2016; Gilbert et al., 2016; Glass et al., 2017, 2021; Hegarty et al., 2019; Littleton et al., 2016; Stevens et al., 2015; Tiwari et al., 2010 ***)*** when there were concerns in at least one domain, but not enough to indicate a high risk of bias in any domain.
3. Two studies were rated ***low risk of bias*** (Ford‐Gilboe et al., 2020; Koziol‐McLain et al., 2018) if the study had a low risk of bias across all its six domains.

We suggest caution when interpreting RCT risk of bias ratings when reporting the certainty of the evidence for digital interventions for IPV survivors' mental health and victimization.

#### 5.7.1 Allocation (selection bias)

Most of the RCTs reported their randomization procedures, utilizing computer‐based randomized algorithms to assure equitable allocation. For example, Koziol‐McLain et al. (2018) reported that “the participant's allocation was kept secret from herself and the study team in New Zealand” (p. 3). Nonetheless, a number of studies lacked data on participant allocation concealment ‐ that is, both participants and researchers were able to identify the group to which they had been assigned. Therefore, participants may have predicted their group based on the intervention components and style of material (e.g., telephone intervention). In Hegarty et al. (2019); “Women were masked to treatment allocation, although some may have guessed which website they were receiving.

#### 5.7.2 Blinding (performance bias and detection bias)

Blinding was an area of low risk in several studies. However, blinding was impossible in some trials, given the nature of the intervention delivered (i.e., technology‐based). In some cases, researchers had to conduct mini‐orientation sessions for survivors using proprietary apps or new platforms, making it hard to conceal intervention arms. It was, however, possible to blind some study staff in some RCTs. For example, Stevens et al. (2015) reported their research assistant was blinded to study conditions. Tiwari et al. (2010) reported their outcomes assessors were “not involved in the design of the study, did not know the study hypotheses, and were blinded to group assignment.” Braithwaite and Fincham (2014) mentioned their participants were blinded to their allocated condition, but the researcher was not. All the research team [was] masked to participant allocation until after analyzing the 12‐month data.”

#### 5.7.3 Incomplete outcome data (attrition bias)

Attrition across all time points was small and largely due to losing contact with survivors. Retention rates were on average about 75% at 3–6 months post‐intervention and <70% at greater than 6 months follow‐up. Higher attrition rates were more common for modular‐based intervention, where module completion rates diminished with time. For instance, Littleton et al. (2016) stated of the 38 survivors in the intervention group, only six participants (15.8%) completed the entire program. For this reason, most studies used an Intent‐to‐Treat analytical method to account for attrition and loss to follow up among their participants.

#### 5.7.4 Selective reporting (reporting bias)

Five RCTs published their protocol (e.g., Decker et al., 2020; Ford‐Gilboe et al., 2020; Glass et al., 2017; Koziol‐McLain et al., 2016; Sabri et al., 2019). Some RCTs found unfavorable or surprising results for particular outcomes, and reported these. Using phrases such as “findings were not statistically significant” and “changes over time did not differ by research arm,” negative findings were based on an alpha of *p* > 0.05. The majority of studies, however, gave substantial information on negative findings and likely explanations for null results. These null or negative outcomes were reflected in our meta‐analysis impacting the overall effect sizes.

#### 5.7.5 Other potential sources of bias

Pearson's correlation coefficients show anxiety significantly correlates with depression. This correlation potentially introduces bias in distinguishing the effects of digital interventions on both outcomes separately (see Fiorillo et al., 2017). In some cases, mental health was not a direct target of technology‐based therapies, making it difficult to ascribe improved outcomes to a particular intervention. It is likely that survivors sought out additional methods to improve their mental health and decrease victimization.

Furthermore, some technology‐based interventions were not in themselves designed to “reduce IPV.” For example, the ePREP was designed to “teach conflict management, communication skills training and the generally seek to improve relationship skills” (Braithwaite & Fincham, 2014). Others used technology as a way to deliver an already existing face‐to‐face intervention. For instance, Constantino et al., 2015 delivered the HELPP intervention in online and face‐to‐face formats and compared this to a control condition, making it challenging to ascribe intervention effects to technology alone.

Another potential source of bias was data dependence. Braithwaite and Fincham (2011, 2014) measured IPV in couples as a dyad, potentially introducing dependence of couples' data. They addressed this by striving for empirical distinguishability so that male and female partner data could be extracted separately.

### 5.8 Effects of interventions

In the results section, we first summarize each outcome in plain language. Then, we show the pooled effect results for each outcome, both with and without outliers.

**Table:** 

IPV outcomes	Time points analyzed^‡^	Results favor digital intervention	No difference	Results favor control
Depression	3 months	Yes**	‐	‐
	3–9 months	Yes^NS^	‐	‐
	10+ months	Yes^NS^	‐	‐
Anxiety	0–3 months	Yes**	‐	‐
PTSD	3–6 months	Yes^NS^	‐	‐
Physical violence victimization	0–6 months	Yes**	‐	‐
	>6 months	‐	‐	Yes^NS^
Psychological violence victimization	0–6 months	‐	‐	Yes^NS^
Sexual violence victimization	>6 months	‐	Yes^NS^	‐
	6–9 months	‐	‐	Yes**

^‡^Outliers removed; Yes**, Significant at *p* < 0.05; Yes^NS^, non‐significant at *p* < 0.05.

### 5.9 Meta‐analysis of depression

Key finding: Between 0 and 3 months post‐intervention, IPV survivors who received technology‐based interventions exhibited a small but significant reduction in depression. However, this effect faded after 3 months and was highest immediately post‐intervention.

#### 5.9.1 Depression at ≤3 months post‐intervention follow‐up

**Figure 3:**
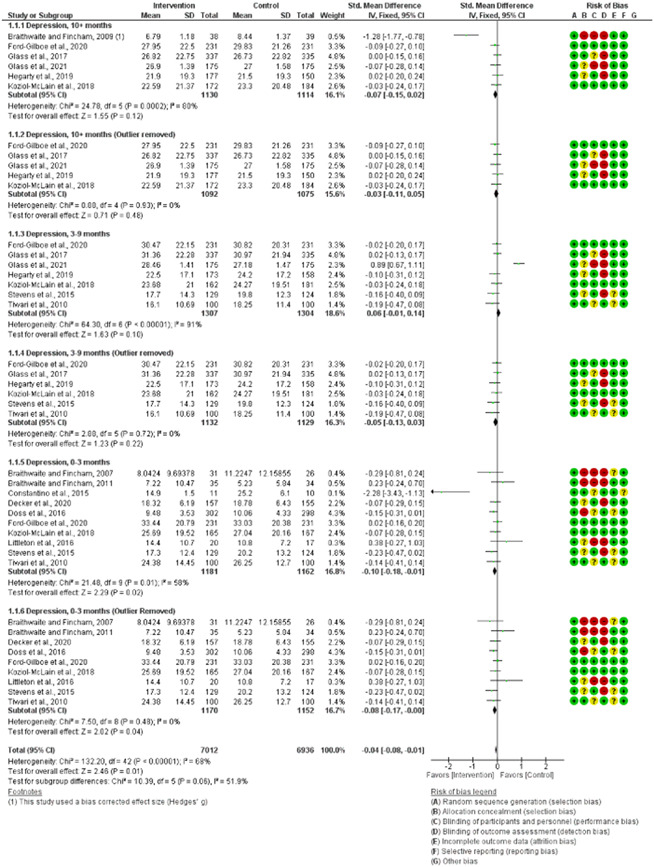
(Analysis 1.1) Forest plot of comparison: Depression Outcome at 0–3, 3–9, and 10+ months post‐intervention (with and without outliers)

At less than 3 months, nine RCTs provided data for a pooled effect size for depression (Braithwaite & Fincham, 2007, 2011; Constantino et al., 2015; Decker et al., 2020; Doss et al., 2016; Ford‐Gilboe et al., 2020; Koziol‐McLain et al., 2018; Littleton et al., 2016; Stevens et al., 2015; Tiwari et al., 2010). Across these nine RCTs, study effect sizes ranged from −2.37 (Constantino et al., 2015) to 0.39 (Littleton et al., 2016). A fixed‐effect model revealed a small, but statistically significant negative effect (as in reduction in) depression (SMD = −0.10, 95% CI = −0.18 to −0.01; *I* ^2^ = 58%). Heterogeneity was significant. Figure 3 shows a forest plot for depression at ≤3 months.

##### Without outlier(s)

After removing a significant outlier RCT with a large effect size favoring digital interventions (Constantino et al., 2015), the remaining eight RCTs still produced a small, statistically significant negative effect (as in reduction) in depression (SMD = −0.08, 95% CI = −0.17 to −0.00; survivors = 2322; studies = 9; *I* ^2^ = 0%). No significant heterogeneity was noted after removing this outlier. Figure 3 shows the forest plot of depression at <3 months. Of note, studies were identified as outliers when their 95% CI fell outside the 95% CI of the pooled effect. Using the “leave‐one‐out” method, we assessed the influence of extreme case RCTs (e.g., studies with over positive or negative effect sizes were removed).

#### 5.9.2 Depression at 3–9 months post‐intervention follow‐up

Seven RCTs provided effect sizes to calculate the overall ES for depression between 3 and 9 months. The SMD effect sizes ranged from −0.195 (Tiwari et al., 2010) to 0.89 (Glass et al., 2021). Our fixed‐effect model of these seven RCTs (Ford‐Gilboe et al., 2020; Glass et al., 2017, 2021; Hegarty et al., 2019; Koziol‐McLain et al., 2018; Stevens et al., 2015; Tiwari et al., 2010) revealed a small but non‐significant positive effect (as in increase in) depression (SMD = 0.06, 95% CI = −0.01 to 0.14; survivors = 2611; studies = 7; *I* ^2^ = 91%). Significant heterogeneity was noted here.

##### Without outlier(s)

After removing an outlier RCT with a large effect size favoring the control group (Glass et al., 2021), we still found a small but non‐significant negative effect (as in reduction in) depression at 3–9 months (SMD = −0.05, 95% CI = −0.13 to 0.03; survivors = 2261; studies = 6; *I* ^2^ = 0%). No significant heterogeneity was noted. Figure 3 shows the forest plot of depression at 3–9 months.

#### 5.9.3 Depression at 10–12 months post‐intervention follow‐up

Effect sizes for depression in this time frame ranged from −1.29 (Braithwaite & Fincham, 2009) to 0.02 (Hegarty et al., 2019). The overall fixed‐effect model from six RCTs revealed a small but non‐significant negative effect (as in reduction in) depression (SMD = −0.07, 95% CI = −0.15 to 0.02; survivors = 2244; studies = 6; *I* ^2^ = 80%). Figure 3 shows the forest plot of depression at 10–12 months.

##### Without outlier(s)

After removing an outlier RCT with a large effect size favoring digital interventions (Braithwaite & Fincham, 2009), the remaining five RCTs showed digital interventions had a small, negative (as in reduction in) effect on depression among IPV survivors that was non‐significant at 10–12 months (SMD = −0.03, 95% CI = −0.11 to 0.05; survivors = 2167; studies = 5; *I* ^2^ = 0%). No significant heterogeneity was noted.

Overall, there were variances in how digital interventions impacted depression symptoms at the study level. Some trials found no difference (Glass et al., 2017, 2021; Hegarty et al., 2019; Stevens et al., 2015). However, others found significant reductions in depression in the intervention more than in the control group (Constantino et al., 2015; Doss et al., 2016; Ford‐Gilboe et al., 2020; Littleton et al., 2016). Decker et al. (2020) found depression reduced in both intervention and control groups. In Koziol‐McLain et al. (2018) there was also a significant intervention effect for reducing depression symptoms for Māori women at three months but not at 6 or 12 months. In the study by Glass et al. (2017); at 12 months, there were no significant between‐group differences in IPV, depression, or PTSD. Still, intervention women had a greater increase in safety behaviors at 12 months and were more likely to have left the abusive partner. However, in Ford‐Gilboe et al. (2020); women in both intervention and control groups improved over time on depression (*p* < 0.001) and on all secondary outcomes.

### 5.10 Meta‐analysis of anxiety at 0–3 months

Key finding: Digital interventions reduced anxiety among survivors. This effect size was small and significant up to 3 months post‐intervention.

**Figure 4:**
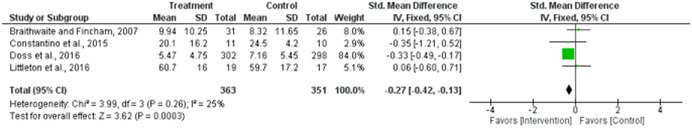
(Analysis 1.2) Forest plot of comparison: Anxiety Outcome (0–3 months)

From four RCTs (Braithwaite & Fincham, 2007; Constantino et al., 2015; Doss et al., 2016; Littleton et al., 2016), we found digital interventions had a small significant reduction in anxiety between 0 and 3 months (SMD = −0.27, 95% CI = −0.42 to −0.13; survivors = 714; studies = 4; *I* ^2^ = 25%) with the outlier removed. No significant heterogeneity was noted. Only two RCTs provided follow‐up information for anxiety at >6 months, so we did not meta‐analyze any data for anxiety beyond three months. Figure 4 shows a forest plot for anxiety.

### 5.11 Meta‐analysis of PTSD at 3–6 months

Key finding: Digital interventions reduced PTSD among IPV survivors. The effect was small and non‐significant between 3 and 6 months post‐intervention compared to controls.

**Figure 5:**
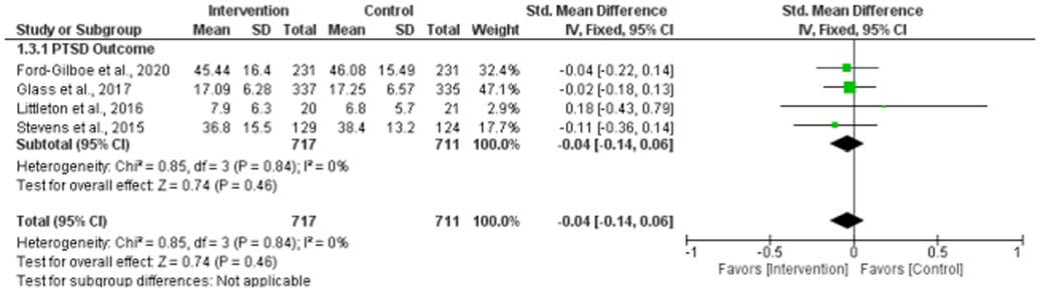
(Analysis 1.3) Forest plot of comparison: PTSD Outcome (3–6 months)

Four RCTs provided data to calculate a pooled effect for PTSD for 3–6 months post‐intervention (Ford‐Gilboe et al., 2020; Glass et al., 2017; Littleton et al., 2016; Stevens et al., 2015). Only two studies provided data for PTSD at 12 months follow‐up (Ford‐Gilboe et al., 2020; Glass et al., 2017), so we did not meta‐analyze any data beyond 6 months. Meta‐analysis of post‐intervention PTSD outcome demonstrated interventions had a small but non‐significant negative effect (as in reduction in) PTSD (SMD = −0.04, 95% CI = −0.14 to 0.06; survivors = 1428; studies = 4; *I* ^2^ = 0%). No significant heterogeneity was observed (*Q* = 0.87, *p* = 0.83). Figure 5 shows a forest plot for PTSD.

### 5.12 Meta‐analysis of physical violence victimization

Key finding: Digital Interventions reduced survivors' physical violence victimization, although treatment effect sizes were small but significant from 0 to 6 months post‐intervention.

For physical violence victimization, we meta‐analyzed two time points (0–6 months and >6 months):

#### 5.12.1 Physical violence victimization at 0–6 months follow‐up

At 0–6 months, six RCTs provided data to calculate a pooled effect size (Braithwaite & Fincham, 2007; Braithwaite & Fincham, 2014; Gilbert et al., 2016; Koziol‐McLain et al., 2018; Tiwari et al., 2010; Zlotnick et al., 2019). We found a moderate, but statistically significant negative effect (as in reduction in) physical violence victimization (SMD = −0.45, 95% CI = −0.59 to −0.32, *p* < 0.01, survivors = 883). No significant heterogeneity was noted (*Q* = 5.45, *p* = 0.25).

##### Without outlier(s)

**Figure 6:**
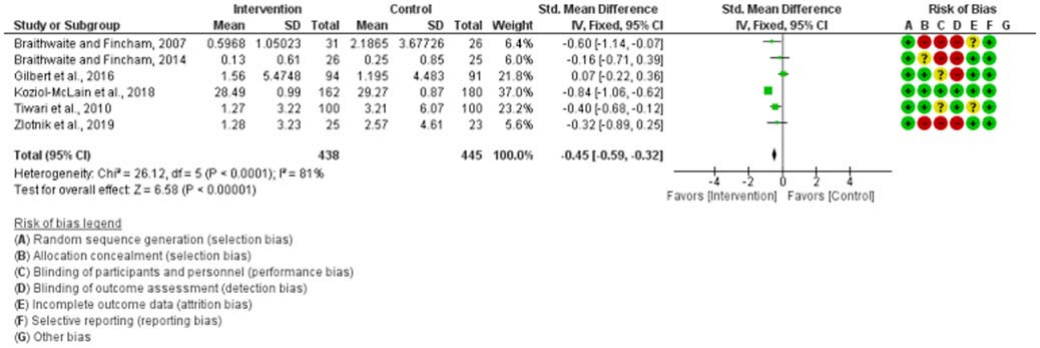
(Analysis 1.4) Forest plot of comparison: Meta‐analysis of Physical Victimization, 0–6 months

**Figure 7:**
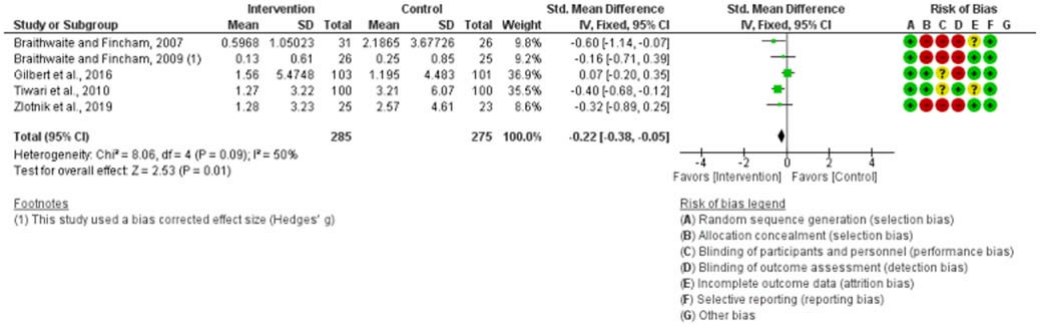
(Analysis 1.5) Forest plot of comparison: Meta‐analysis of Physical Victimization, 0–6 months (Outlier Removed)

Koziol‐McLain et al. (2018) was a significant outlier, and when removed (*k* = 5, *n* = 560 survivors), we had a small but statistically significant negative effect (as in reduction in) physical violence victimization (SMD = −0.22, 95% CI = −0.38 to −0.05, *p* < 0.01). Koziol‐McLain et al. (2018) state some of their estimates though significant, may have been “imprecise, with wide confidence intervals” (p. 12). As a result, the Koziol‐McLain et al. (2018) RCT was a significant outlier across effect size comparisons for IPV victimization but not for mental health outcomes. Figures 6 and 7 show a forest plot for physical violence victimization at 0–6 months (with and without outliers).

#### 5.12.2 Physical violence victimization at >6 months follow‐up

At more than 6 months, six RCTs showed that digital interventions had a small, non‐significant negative effect (as in reduction in) physical violence victimization (SMD = −0.05, 95% CI = −0.15 to 0.05, *p* < 0.01, survivors = 1540). Significant heterogeneity was observed (*Q* = 186.67, *p* < 0.01, *I* ^2^ = 97.32%).

##### Without outlier(s)

When two outlier studies were removed (Braithwaite & Fincham, 2009; and Koziol‐McLain et al., 2018) with extremely large effect sizes favoring digital interventions and controls, respectively, the remaining four RCTs showed a small, non‐significant, and positive (as in increase in) physical violence victimization (SMD = 0.04, 95% CI = −0.08 to 0.15; survivors = 1108). However, no significant heterogeneity was observed (*Q* = 6.93, *p* = 0.07).

### 5.13 Meta‐analysis of psychological violence victimization

Key findings: There appeared to be an unanticipated increase in post‐intervention psychological violence victimization between 0 and 6 months. However, there was a small reduction in psychological violence victimization after >6 months, indicating a delayed intervention effect that could be due to several critical determinants in the **survivor's** environment, such as increased abuse and harassment at the onset of participation in the RCT, followed by the survivor trying out and mastering new coping and safety strategies, and becoming aware of psychological abuse over time. It is also likely survivors became better at concealing their participation in the intervention.

For psychological violence victimization, we meta‐analyzed two time points (0–6 months and >6 months).

#### 5.13.1 Psychological violence victimization at 0–6 months follow‐up

Six studies reported outcomes for psychological violence victimization at 0–6 months (Braithwaite & Fincham, 2007; Braithwaite & Fincham, 2014; Koziol‐McLain et al., 2018; Stevens et al., 2015; Tiwari et al., 2010; Zlotnick et al., 2019), and demonstrated a small, statistically significant negative effect (as in decrease in) psychological aggression at 0–6 months (SMD = −0.45, 95% CI = −0.59 to −0.32; survivors = 883; studies = 6; *I* ^2^ = 81%). However, the number of comparisons was small and significant heterogeneity of effects was noted (*Q* = 77.44, *p* < 0.01). Although this analysis looked promising for reducing psychological aggression, the Koziol‐McLain et al. (2018) study was a significant outlier, pulling the summary effect size estimate to the left (favoring the intervention group).

##### Without outlier(s)

After removing this outlier (Koziol‐McLain et al., 2018) with a large effect size favoring digital interventions, we found digital interventions had a small, positive effect (as in increase in), non‐significant effect on psychological aggression at 0–6 months (SMD = −0.34, 95% CI = −0.47 to −0.20; studies = 6; *I* ^2^ = 94%), with significant heterogeneity observed.

#### 5.13.2 Psychological violence victimization at >6 months follow‐up

Five RCTs reported outcomes for psychological violence victimization at >6 months (Braithwaite & Fincham, 2007, 2014; Glass et al., 2017; Koziol‐McLain et al., 2018; Tiwari et al., 2010). We found a small, statistically significant negative effect (as in decrease in) psychological aggression at >6 months (SMD = −0.29, 95% CI = −0.39 to −0.18; survivors = 1357; studies = 5; *I* ^2^ = 96%). However, the number of comparisons were small and significant heterogeneity of effects was noted (*Q* = 107.10, *p* < 0.01).

##### Without outlier(s)

**Figure 8:**
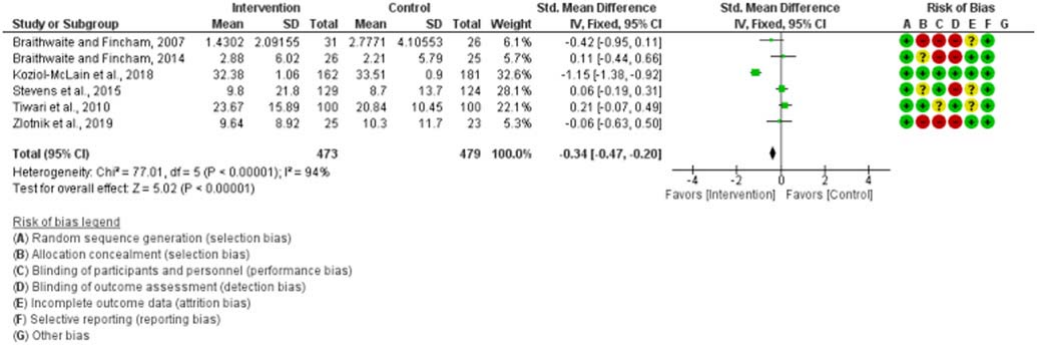
(Analysis 1.8) Forest plot of comparison: Meta‐analysis Psychological Victimization, 0–6 months

**Figure 9:**
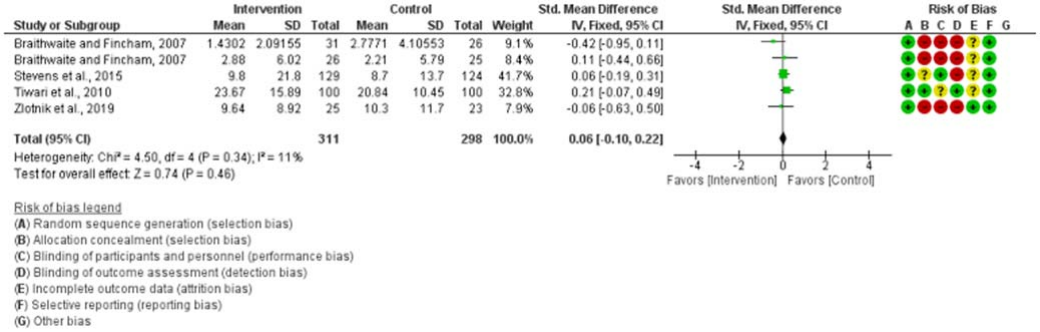
(Analysis 1.9) Forest plot of comparison: Meta‐analysis of Psychological Victimization, 0–6 months (Outlier Removed)

**Figure 10:**
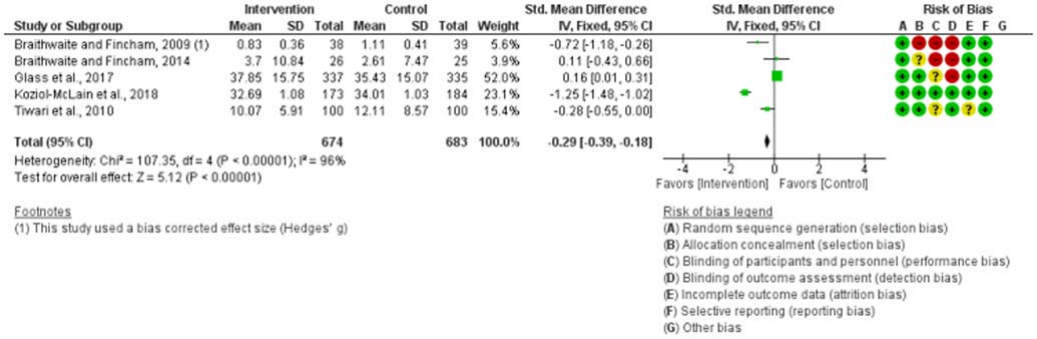
(Analysis 1.10) Forest plot of comparison: Meta‐analysis of Psychological Victimization, >6 months

**Figure 11:**
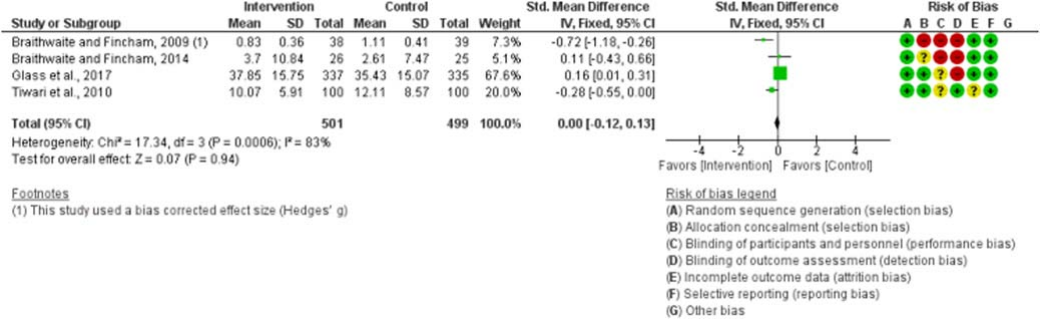
(Analysis 1.11) Forest plot of comparison: Meta‐analysis of Psychological Victimization, >6 months (Outlier Removed)

After removing an outlier study (Koziol‐McLain et al., 2018), we found an equivalent effect (as in no difference between groups) on psychological aggression at >6 months was (SMD = 0.00, 95% CI = −0.12 to 0.13; survivors = 1000; studies = 4; *I* ^2^ = 83%); significant heterogeneity of effects was noted (*Q* = 15.14, *p* = 0.002). Figures 8, 9, 10, 11 shows forests plots for psychological violence victimization at 0–6 and >6 months, with and without outliers.

### 5.14 Meta‐analysis of sexual violence victimization at 6–9 months

Key findings: Between 6 and 9 months, there seemed to be a decrease in post‐intervention sexual violence victimization. However, this effect was not statistically significant. We did not have enough studies to run an analysis for an earlier time point.

Four RCTs reported outcomes for sexual violence victimization (i.e., Gilbert et al., 2016; Glass et al., 2017; Koziol‐McLain et al., 2018; Tiwari et al., 2010). We meta‐analyzed one time point (6–9 months) due to the limited number of studies providing enough information to calculate an overall effect size. Meta‐analysis of post‐intervention sexual violence outcome demonstrated interventions had a small, significant positive effect (as in increase in) sexual violence victimization (SMD = 0.32, 95% CI = 0.21 to 0.43; participants = 1402; studies = 4; *I* ^2^ = 98%). Significant heterogeneity was observed (*Q* = 153.21, *p* < .01).

#### Without outlier(s)

**Figure 12:**
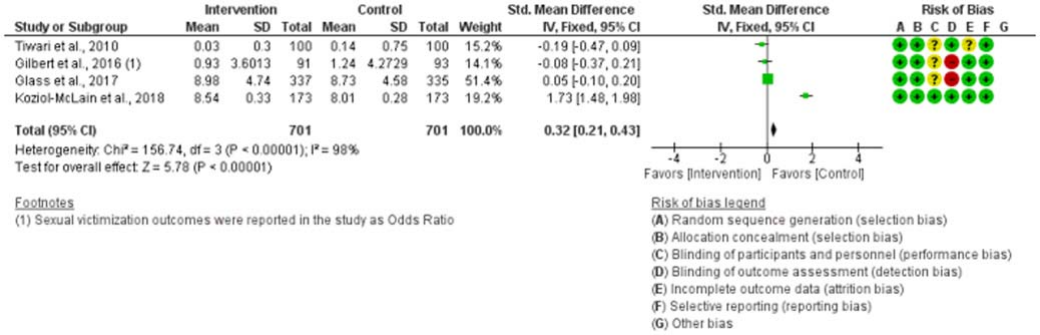
(Analysis 1.12) Forest plot of comparison: Sexual Victimization, 6–9 months

**Figure 13:**
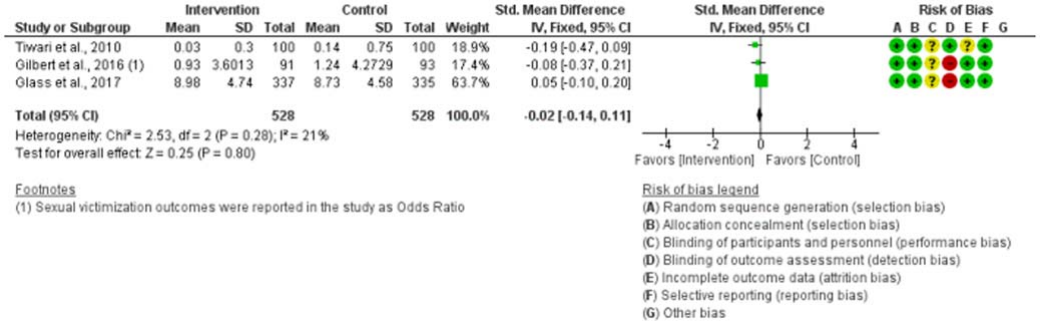
(Analysis 1.13) Forest plot of comparison: Meta‐analysis of Sexual Victimization, 6–9 months (Outlier Removed)

After removing an outlier study (i.e., Koziol‐McLain et al., 2018) with an extremely large effect size favoring controls, meta‐analysis of post‐intervention sexual violence outcome (*k* = *3, n* = 1057 survivors) demonstrated a small non‐significant negative effect (as in decrease in) sexual violence victimization (SMD = −0.02, 95% CI = −0.14 to 0.11; participants = 1056; studies = 3; *I* ^2^ = 21%). Significant heterogeneity was observed (*Q* = 3.1, *p* = 0.21). Figures 12 and 13 show a forest plot for sexual violence victimization (with and without outliers).

## 6 DISCUSSION

This is the first meta‐analysis examining the omnibus effects of technology‐based interventions on mental health and victimization outcomes among IPV survivors. From 17 RCTs, we found that among female survivors of IPV, digital and technology‐based interventions significantly reduced depression (at 3 months), anxiety (at 3 months), and physical victimization (at 6 months). However, during the follow‐up, these effects appeared to fade with time. We found significant reductions in psychological violence victimization at 0–6 months and at >6 months; however, at both time points, there were outlier studies. There was no significant change in experiences of PTSD or sexual victimization at any time.

Overall, this meta‐analysis demonstrated the practical benefits of using digital interventions to support mental health and reduce IPV victimization among survivors, if only in the short term (0–3 months), particularly at the height of emotional, mental, and relationship distress, such as when survivors are seeking care in health care clinics, crises centers, meeting with an advocate or searching for resources online. However, these small effect sizes potentially reflect methodological issues with randomizing survivors to RCTs, participant diversity, limitations at the study level, and the challenge of responding to IPV using a digital intervention.

It is also worth noting that technology‐based interventions support other outcomes not reported here. For instance, Glass et al. (2017) reported that the myPlan app reduced decisional conflict and improved engagement with safety strategies. They also find these interventions may help high‐risk women safely navigate ending abusive relationships and reduce future IPV exposure (p. 613).

Additionally, we found little evidence that digital interventions are being adapted for ethnically, culturally and linguistically diverse survivors. Although, Koziol‐McLain et al. (2018) found efficacy in using this intervention with indigenous Māori women, Eden et al. (2015); Glass et al. (2017) also found these interventions efficacious with Spanish‐speaking women using adapted versions of already existing proprietary interventions. However, considering the burden of IPV among women in the Global South, more evidence‐based, age‐appropriate, language and culturally congruent interventions are needed. Of note, accessing technology‐based interventions might pose a challenge in low‐tech or no‐tech settings, innovative approaches are warranted in these contexts.

Several strengths of our analysis should be emphasized. First, the large number of participants involved in our meta‐analysis (*N* = 4590) strengthened the reliability of our pooled effect sizes. Second, we reported effect sizes using a more stringent fixed‐effects model (FEM); therefore, our finds are the most conservative they can be. Otherwise, we would have had slightly inflated effects sizes if we had used a random‐effects model (REM). Finally, our analysis provides provisional but promising findings that can support research proposals, funding goals, research writing, and policy considerations as we continue to find evidence‐based, trauma‐informed, but pragmatic ways to support survivors. Importantly, our findings add to the body of evidence regarding the limitations of digitally delivered interventions for IPV survivors and contribute to a more contextualized and nuanced understanding of these interventions.

Our findings add to existing knowledge of *what works* to support survivors' mental health, safety, and well‐being. However, additional moderator analyses are needed to identify active intervention ingredients that worked, map out intervention mechanisms of action (e.g., how is mental health ameliorated?), best modes of delivery (e.g., smartphone apps vs. online formats), adequate dosage levels (i.e., therapeutic dose) using the treatment intensity matching process, and guidelines to increase feasibility and acceptability. As the field advances, some of these issues will lend themselves to rigorous assessments; however, this field remains emergent at this time.

Survivors deserve all the tools in our arsenal. Digital interventions remain essential to our current response to reach survivors where they want to be contacted. In this way, we can bridge the gap between service providers and survivors, particularly those in isolated situations and contexts, as was the case during the COVID‐19 lockdowns. However, we still do not know how survivor characteristics that worsened domestic violence risk during the pandemic (such as changing risk levels due to stay‐at‐home, social distancing mandates, technology illiteracy, low socioeconomic status, and polyvictimization) influenced the uptake and use of digital interventions.

Importantly, we need to consider how these digital interventions may become valuable tools for working with other populations, such as men and boys who are abusive in relational contexts. Work with violence perpetration has not been met with the same technological advancement as we see in our work with survivors. In principle, digital interventions can be used to deliver interventions focused on general psychoeducation, emotional regulation, conflict resolution, anger management, or other specialized therapeutic interventions (e.g., cognitive‐behavioral therapies) to boys and young men to promote behavior change before violence occurs. These interventions can also be used as rehabilitative tools for partner‐abusive men who are thinking about ways to stop using violence (e.g., the treatment curious) or those already mandated by legal systems to receive therapy as an add‐on to treatment as usual (i.e., treatment veterans).

### 6.1 Limitations

Some RCT‐specific limitations may have hampered findings in this meta‐analysis. For example, participant characteristics varied across RCTs, suggesting that the observed summary effects are not generalizable to all survivor populations.

Another limitation to this study was the ethical and safety concerns with *truly* randomizing IPV survivors into control conditions, making trials unpopular with IPV survivors. However, irrespective of the treatment arm, survivors were offered relevant local and national resources outside the primary intervention.

Intervention content may have been incompletely coded due to the lack of information in study reports. Likewise, due to the small number of comparisons, certain variables of interest could not be included in this meta‐analysis. In addition, some trials had three intervention arms, which we included. We created a pair‐wise comparison by splitting the control group into smaller groups to integrate all data from these trials. This strategy, however, may result in more errors in the meta‐analysis. We did not examine publication bias and cannot determine if critical but unpublished studies may have been missing from this meta‐analysis.

Furthermore, even though the participants reported mental health issues like depression, anxiety, and PTSD, it was hard to tell if these conditions were clinically diagnosed, self‐reported, or somatized because of IPV exposure. It is highly likely an IPV survivor with major depressive disorder (MDD) may respond differently to a digital intervention from one with persistent depressive disorder (PDD), and so on. In many RCTs reviewed here, mental health and victimization outcomes were self‐reported. Koziol‐McLain et al., 2018 indicate that *self‐reporting* may introduce common method biases—leading to “inflated relationships between variables measured” that can upwardly bias relationships (Conway & Lance, 2010).

Similarly, we must assess whether digital interventions are clinically beneficial for survivors who meet clinical diagnostic criteria for complete or sub‐threshold PTSD, depression, and anxiety. This is a complex undertaking that is pertinent to designing and evaluating technology‐based interventions. To address this, Littleton et al., 2016 used the reliable change index (RCI) to convert their raw findings to clinically meaningful scores in the form of standardized Z‐scores so that values more than 1.96 (or one standard deviation) were considered clinically significant and vice versa (Littleton et al., 2016; citing Jacobson & Truax, 1991).

In the same way, self‐report data may have skewed reports of mental health and functioning. The stigmatizing nature of help‐seeking for some survivors makes it likely that self‐report data captured some social desirability bias. In addition, we cannot ignore that abusers may have influenced survivors' responses to IPV surveys—particularly in studies using couple dyads. The diverse IPV measures we found across RCTs also bear mentioning. These diverse instruments may have captured different dimensions of IPV, and with little to no convergent validity, our measure of IPV pooled effects is likely biased. Although using SMDs controls for this divergence in outcomes measures, so we can compare survivors across studies. In addition, existing IPV instruments have predominantly been developed and validated with western populations in high‐income countries, leaving out survivors in non‐Western and low‐income contexts (Goessmann et al., 2021).

Furthermore, Koziol‐McLain et al. (2018) explain, “Engaging with these measurement items may have raised women's awareness of the violence in their relationships and muted an intervention effect” (p. 12). Some participants may have been therapized, given these validated measures ask about specific behaviors of the abusive partner, forced sex, attempted strangulation, and strategies for safety—these likely impact the control group and may increase their awareness of resources to mitigate IPV. This effect is known as “regression to the mean” and is prevalent in psychological therapies. Therefore, researchers urge more prudent study designs (such as the Solomon four‐group design) to retain the internal validity of the trial by taking into consideration the reactivity of baseline and follow‐up measurements. Conducting sensitivity analyses or controlling for baseline characteristics are further strategies to lessen this interference.

Since the quality of included RCTs varied, study‐level biases may have introduced biases in this meta‐analysis, limiting our findings' generalizability. Furthermore, we could not further explore the heterogeneities we saw in our data using moderator analyses, given the small number of studies, limiting how much we can know from this available data. Although, effect sizes are not dependent on sample size. Lastly, we performed a comprehensive literature search. However, we did not include unpublished research. Thus our overall effect sizes are likely to be upwardly biased.

#### 6.1.1 Limitations of using digital interventions for IPV

Concerns have been raised concerning the safety, privacy, transparency, and equitable access to these digital interventions (Emezue, 2020). Digital divides, for example, hinder survivors' access to digital interventions, particularly in under‐resourced areas. Survivors with intellectual or cognitive disabilities (e.g., older age groups and neurodiverse survivors, deaf and hard‐of‐hearing survivors) may also be resistant to new technologies—at least in the way they are currently designed. Because smartphones and internet services are so costly, device disparities frequently impede access to digital support, making it impossible for low‐income and no/low‐tech survivors to find use in this type of intervention.

Additionally, some abusers covertly track the survivor's online presence, presenting potential safety risks based on their documented use of spyware to monitor and infiltrate support spaces online under false pretexts and using social engineering to surveil their partners. Abusive partners (primarily males) pose a sophisticated obstacle to survivors' use of digital interventions (Emezue, 2020).

### 6.2 Summary of main results

This study is the first to conduct a comprehensive review and meta‐analysis of technology‐based interventions to improve mental health and IPV outcomes in female survivors of IPV. We observed some effectiveness in employing technology‐based interventions to support survivors, namely in lowering IPV‐related depression (at 3 months), anxiety (at 3 months), and physical victimization (at 6 months), with marginal reductions in psychological victimization. Despite the effect sizes being modest and inconsistent with earlier meta‐analyses on this topic (see Agreements and disagreements with other studies or reviews). The modest effect sizes in this report demonstrate the difficulty in stopping violence by involving survivors alone.

Beyond mental health, violence interventionists must be aware of the difficulties in improving IPV outcomes. The fact that digital interventions were more successful for mental health suggests that better IPV outcomes are unlikely to be attained only through technology alone. Instead, violence interventionists who use technology‐based interventions as an additional layer of support for survivors will be more successful.

### 6.3 Overall completeness and applicability of evidence

We started this meta‐analysis by doing a thorough and systematic search of the literature, which provided a large number of citations to sort through and ensured that all relevant research was found and retained. Researchers whose data we needed for our meta‐analysis were also contacted. We did two rounds of searches to ensure that no studies were missed, but it is possible that some were. Between March and April 2019 (14 studies were found) and March 2021, the searches were conducted (two studies were added). During the review of this manuscript for publication, an unknown peer‐reviewer suggested a final study, bringing the total number of studies to 17 RCTs.

### 6.4 Quality of the evidence

Two RCTs were considered to have a *low risk* of bias (Ford‐Gilboe et al., 2020; Koziol‐McLain et al., 2018). Four RCTs had at least three “*high risk*” violations and were considered *high risk* (Braithwaite & Fincham, 2007, 2009, 2011; Zlotnick et al., 2019 ‐ all for selection, performance, and detection bias) (see Figure 2). The remaining 11 RCTs were considered *moderate risk*. Where bias was “*unclear,”* it was due to vague descriptions of allocation concealment (selection bias) and an ambiguous description of participant and personnel blinding (performance bias). The least “*low risk”* bias violations were for random sequence creation (selection bias), and the most “*high risk”* bias violations were for outcome assessment blinding (detection bias).

### 6.5 Potential biases in the review process

We want to highlight several shortcomings that may have caused biases.

1. First, we searched for our RCTs published in English, missing non‐English‐language studies in non‐English‐speaking nations, especially LMICs.
2. Second, We only discovered reports from mostly middle‐ to high‐income Canada, Australia, New Zealand, and China (People's Republic of); with only one study from Kenya. This limitation does not hinder our interpretation. We recommend more research in LMICs with high IPV prevalence and low/no‐data environments where digital therapies are less popular.
3. Lastly, some studies employed couple dyads with women and men as survivors and abusers. Despite being divided by gender and outcome, this data may have been dependent. To address this, we solely collected data from female survivors and excluded outlier studies, in addition to the more stringent fixed‐effect analysis performed here.

### 6.6 Agreements and disagreements with other studies or reviews

Two prior meta‐analyses evaluating the impact of these technology‐based therapies on IPV mental health and victimization outcomes have been published, although diverging from the meta‐analysis in their coverage, methodology, study's research objectives, and research questions.

Anderson et al. (2019) conducted a systematic review of 31 studies (between 1998 and 2019) and found that digital interventions did not outperform non‐digital interventions, despite high acceptability and feasibility across studies. Anderson et al. (2019) did not conduct a meta‐analysis and combined various study types, including RCTs, non‐randomized studies, descriptive studies, qualitative and mixed‐methods studies. However, our meta‐analysis focused on RCTs only to quantitatively calculate a pooled effect size to support our findings.

More closely linked to our study, Linde et al. (2020) conducted a meta‐analysis of digital interventions in 14 trials on IPV‐related outcomes based on studies in databases from inception until April 2019. Contrary to our findings, they discovered that eHealth interventions did not provide any superior effects to non‐digital modalities. Although they found reductions, these reduction were not significant, with overall IPV (SMD = –0.01; 95% CI = –0.11 to 0.08); physical violence (SMD = 0.01; 95% CI = –0.22 to 0.24; *I* ^2^ = 58%); psychological violence (SMD = 0.07; 95% CI = –0.12 to 0.25; *I* ^2^ = 40%); sexual violence (MD = 0.36; 95% CI = –0.18 to 0.91); depression (SMD = –0.13; 95% CI = –0.37 to 0.11; *I* ^2^ = 78%); and PTSD (MD = –0.11; 95% CI = –1.04 to 0.82). Linde et al. (2020) concluded there was no evidence of a beneficial effect of eHealth interventions on IPV. However, our findings suggest otherwise. This may be because we included almost twice more studies in our meta‐analysis than in Linde et al. (2020) (n=7 vs. 17), we considered a wider time frame (2007–2021), and also included *anxiety* as an outcome measure, given this is an important mental health issue faced by survivors.

Methodologically, our meta‐analysis also differed from Linde et al. (2020); who used mean difference (MD) and SMD. We only used SMD in this meta‐analysis—as the RCTs in this meta‐analysis measured the same outcome using various validated instruments. Another important difference was that Linde et al. (2020) used a *random‐effects model*, whereas we used a more parsimonious *fixed‐effects model*, given the small number of studies. Like Linde et al. (2020); we found heterogeneities across studies. In sum, where Linde et al. (2020) found no effect, we found small but significant time‐based reductions in depression, anxiety, and physical violence victimization.

## AUTHORS' CONCLUSIONS

### 1 Implications for practice

This review highlights several benefits of using digital interventions to support survivors' mental health, as a practical strategy. We caution, however, that in some RCTs, technology was used purely as a delivery mechanism of an existing in‐person intervention. There are several advantages to deploying interventions in this way—including increasing confidentiality, around‐the‐clock reliability, tailoring intervention to survivors, and expanding access to our most underserved survivors. These advantages mitigate several barriers that survivors face in receiving care, making a compelling case for technology‐based intervention.

Moreover, we were not surprised by the small but significant decreases in depression and anxiety, particularly in the short term. Some survivors are motivated to enroll in research studies when things are particularly distressful in the relationship. Mental health symptoms are likely to be so high at this point that baseline measures may be higher in both groups and thus highly responsive to any intervention (Koziol‐McLain et al., 2018). Furthermore, depression and anxiety, while chronic, tend to regress to the mean over time (meaning overtime, if measured repeatedly, the variable tends to even out or return to normal). This can happen due to the multiple follow‐up time points in RCTs so that even in the absence of intervention or (medical treatment), the value of outcomes starts to return to average (see Golding, 1999).

Furthermore, abusers, for example, have been known to use technology to exert control over their relationships. Tactics such as online monitoring, GPS tracking of their partner's whereabouts, and even sabotaging online communities for survivors continue to expose survivors to worsening violence and an increased risk of lethality (Emezue, 2020).

A digital divide prevents rural, vulnerable, and low‐income survivors from accessing digital interventions in the first place. Not only do some people lack access to phones due to socioeconomic and gender role constraints, but many do not have *smartphones*. Moreover, these interventions may require technological literacy and infrastructure on which to build them (e.g., reliable internet connection). Given these shortcomings, service providers must consider digital interventions as an *add‐on* to usual care (i.e., universal prevention efforts) and as part of a coordinated, collaborative response strategy, so we can continue to scale up interventions that empower survivors in a discretionary and reliable manner.

Importantly, unlike with improving mental health, the idea of “reducing” IPV victimization using a digital intervention places the onus of rehabilitation squarely on the survivors. We know, however, that survivors rarely have any control over the predatory contexts of power and control perpetrated by their abusers. While some interventions we meta‐analyzed here focused on improving relationship health and safety in couple dyads, most recommended survivor‐enforced safety planning strategies or recommendations on how to exit abusive relationships—if the survivor so chooses. Conversely, few interventions jointly involved perpetrators in their intervention efforts, given that this may conceivably escalate violence and lethality in the relationship. Actual reductions in victimization may be accomplished by raising survivors' awareness of the risk of continuous abuse, victimization, and even fatality. Given this, we must map out the theory of change and mechanism of action through which technology can prevent or reduce violence as part of a coordinated community response.

### 2 Implications for research

Given the small number of RCTs on this topic, we join other researchers in calling for high‐quality and rigorous controlled studies. Although, we acknowledge the challenge of randomizing survivors in need of immediate and life‐saving support to controlled interventions. Future studies may devise modalities to minimize these risks while maintaining methodologically and ethically sound randomization procedures. It is not enough to *waitlist* survivors in the control group, as waitlisting may later overestimate intervention effects (as situational mental health issues may resolve on their own without active intervention) (Cunningham et al., 2013)—not to mention waitlisting can be perilous for survivors in need of rapid aid.

Furthermore, it may be helpful for future research to focus on specific intervention designs, device types, digital modalities, and deployment protocols that contributed to the effectiveness of these digital interventions. Future studies will add to the science by identifying active intervention ingredients, mapping out mechanisms of action, best modes of delivery, and guidelines to increase feasibility and acceptability.

Similarly, when tailored to serve historically underrepresented communities, culturally congruent digital IPV interventions were effective. This was proved in the Koziol‐McLain et al. (2018) study with Māori women, suggesting the practicality of culturally congruent adaptations to digital IPV interventions rather than a one‐size‐fits‐all approach. Finally, it will be necessary for future research to consider the restrictions caused by using individual items from specific constructs to gauge an entire outcome, which may limit the measurability of specific outcomes (Doss et al., 2016).

## CONTRIBUTIONS OF AUTHORS

Chuka Emezue (CE), Tina L. Bloom (TLB) drafted and edited the protocol. CE developed a search strategy and searched relevant databases with the help of an expert biostatistician at the University of Missouri‐Columbia. CE and Jo‐Ana Chase (JC, with methodological expertise in meta‐analysis) resolved disagreements on study selection. CE extracted data from trials, collecting articles, coding, synthesizing the articles. JC and CE conducted data entry and coding into review software or other forms. CE and JC worked on practical methodological aspects, main synthesis and analysis. CE, JC, TLB, and Tipparat Udmuangpia (TU) contributed to data interpretation and data analysis. All authors contributed to portions of drafting the final report, and CE had final responsibility for the decision to submit for publication. CE will be responsible for updating this meta‐analysis as the field develops, evidence accumulates, and funding becomes available.

## DECLARATIONS OF INTEREST

Dr. Chuka Emezue is an assistant professor at Rush University College of Nursing, Department of Women, Children and Family Nursing, and was on a multi‐site study involving the myPlan app at the inception of this paper. Likewise, Dr. Tina Bloom is the coauthor of some of the RCTs reviewed here. Other authors declare no competing or conflict of interest.

## PLANS FOR UPDATING THIS REVIEW

Dr. Chuka Emezue assumes primary responsibility for maintaining this review based on emerging interventions, studies, comments and critiques, and other such developments leading to updating the review at least once every 4 years. If Dr. Chuka Emezue can no longer do as stipulated, an appropriate transfer of responsibility will be made as agreed upon with the Campbell Library and Cochrane Social Welfare Group.

## DIFFERENCES BETWEEN PROTOCOL AND REVIEW

There are several variations between the published protocol and this version of the meta‐analysis. However, these differences were not substantial enough to skew our findings:

1. Given that we were interested in randomized controlled trials, the risk of bias of included studies was accessed using the Cochrane risk‐of‐bias tool for randomized trials (RoB 2), as specified in the protocol. As a result, we did not include any non‐randomized studies, nor did we utilize Cochrane's ROBINS‐I (Risk of Bias in Non‐Randomized Studies‐of Interventions) to assess the risk of bias in non‐randomized studies as suggested in the protocol (Sterne et al., 2016).
2. Furthermore, we originally proposed searching for studies from 2009 to 2020. However, during the peer‐review of the protocol paper, we conducted a final search. Thus, we expanded our search to include studies from 2007 to 2021 to capture any new studies in the COVID‐19 era. By doing this, we captured three additional studies that are pertinent to this meta‐analysis. However, these studies did not report on IPV in the era of COVID‐19.
3. We did not perform any statistical testing for funnel plot asymmetry, as none of the pooled outcomes had more than 10 studies as recommended in the Cochrane Handbook for Systematic Reviews of Interventions (Chapter 10.4.3.1.).
4. Changes in authorship: We added Dr. Tipparat Udmuangpia (topic expertise) and Dr. Jo‐Ana Chase (meta‐analysis expertise) to the author list. No authors were removed.

## SOURCES OF SUPPORT

NA

## PUBLISHED NOTES

**Characteristics of included studies**

Braithwaite and Fincham (2007)

**Table:** 

Methods	Three‐arm randomized controlled trial.
Participants	Ninety–one young adult in dating relationships. Women made up 59% of the sample. Caucasian, 60.9%; Asian, 18.7%; African American, 5.5%; and “Other”, 14.3%.
Interventions	Computer–based relationship‐focused preventive intervention (ePREP) relative to a computerized depression and anxiety intervention. Interventions were individually administered computer–based presentations (comprising written text and pictures, no audio or video material was used), the pace of which was controlled by the participant.
Outcomes	Depression, anxiety, IPV (CTS–Negotiation, CTS–Psychological, CTS–Physical), quality of their relationship, Constructive Communication, Trust, Global Relationship Quality
Notes	

Risk of bias table

**Table:** 

Bias	Authors' judgment	Support for judgment
Random sequence generation (selection bias)	Low risk	“Participants were randomly assigned to take part in one of the three interventions.”
Allocation concealment (selection bias)	High risk	There is reason to suspect that the enrolling investigator or the participant had knowledge of the forthcoming allocation. “The interventions were individually administered computer–based presentations (comprising written text and pictures, no audio or video material was used), the pace of which was controlled by the participant…The majority of participants completed the intervention in approximately one hour and all interventions took place in the authors' research laboratory.”
Blinding of participants and personnel (performance bias)	High risk	No blinding reported.
Blinding of outcome assessment (detection bias)	High risk	No blinding reported. “Eight weeks after their initial visit to the lab participants returned to complete the same battery of questionnaires.”
Incomplete outcome data (attrition bias)	Unclear risk	Unclear. Attrition bias suspected.
Selective reporting (reporting bias)	Low risk	“The outcomes from the two treatment conditions did not significantly differ from one another.”
Other bias	Unclear risk	

Braithwaite and Fincham (2009)

**Table:** 

Methods	Randomized controlled trial
Participants	77 college students in romantic relationships of 4 months or longer
Interventions	One hour‐long ePREP. Based on the Prevention and Relationship Enhancement program (PREP, Markman, Stanley, & Blumberg, 2001)
Outcomes	Symptoms of depression and anxiety, IPV, communication patterns, and relationship satisfaction.
Notes	

Risk of bias table

**Table:** 

Bias	Authors' judgment	Support for judgment
Random sequence generation (selection bias)	Low risk	“A computer generated randomization list was used to assign participants to either the ePREP (*n* = 38) or the placebo/control intervention (*n* = 39).”
Allocation concealment (selection bias)	High risk	No allocation concealment reported. There is reason to suspect that the enrolling investigator or the participant had knowledge of the forthcoming allocation.
Blinding of participants and personnel (performance bias)	High risk	No blinding reported.
Blinding of outcome assessment (detection bias)	High risk	No blinding reported.
Incomplete outcome data (attrition bias)	Low risk	Per protocol, completers‐only. The same number randomized were analyzed. “However, maximum likelihood estimation allowed a growth curve to be fit for all respondents even if they did not have data for each time point. Analyses of those who did not complete assessments at the two follow up points did not reveal any main effects of dropping out on the slopes or intercepts of any of the dependent variables”
Selective reporting (reporting bias)	Low risk	No selective reporting suspected. Unfavorable findings were reported.
Other bias	Unclear risk	

Braithwaite and Fincham (2011)

**Table:** 

Methods	Randomized controlled trial
Participants	77 couples (152 individuals). Average age was 19.92 years. Average relationship length (between 1 and 2 years). 20% reported current cohabitation. Overall, 77% White (non‐Hispanic), 10% Latino, 8% Black, 3% “Mixed Race” and 2% Asian. Only one of a dyadic partnership in intervention.
Interventions	Computer‐based preventive intervention (ePREP) versus active placebo control group. ePREP condition taught empirically based methods for improving romantic relationships.
Outcomes	Commitment attitudes, patterns of communication, prior 6 weeks psychological aggression and physical assault, relationship satisfaction and latent variables, depression latent variables, anxiety latent variable.
Notes	

Risk of bias table

**Table:** 

Bias	Authors' judgment	Support for judgment
Random sequence generation (selection bias)	Low risk	“Before coming to the lab, couples were randomly assigned to condition using a computer generated randomization list.”
Allocation concealment (selection bias)	High risk	No allocation concealment reported. There is reason to suspect that the enrolling investigator or the participant had knowledge of the forthcoming allocation.
Blinding of participants and personnel (performance bias)	High risk	No blinding of participants and personnel reported.
Blinding of outcome assessment (detection bias)	High risk	No blinding to outcome assessment noted.
Incomplete outcome data (attrition bias)	Low risk	“Intent to treat analysis all participants were included in the analyses regardless of whether or not they completed the 6 weeks of the intervention and full information maximum likelihood (FIML) estimation was used to impute missing values”
Selective reporting (reporting bias)	Low risk	No selective reporting suspected. Unfavorable findings were reported.
Other bias	Unclear risk	

Braithwaite and Fincham (2014)

**Table:** 

Methods	Randomized controlled trial design comparing ePREP to an active placebo control group
Participants	A community sample of 52 married couples (21% Black, 3% Asian, 65% White, 7% Latino, 4% Mixed/biracial) who had been married, on average, 4.3 years
Interventions	Computer‐based (text and video) preventive intervention (ePREP) versus active placebo control group. ePREP adapted and computerized version of Prevention and Relationship Enhancement program (PREP, Markman et al., 2010). Intervention and control presentation lasted approximately 1 h, followed by weekly homework assignments for the next 6 weeks (1 h per session, completed as a couple).
Outcomes	Self and partner minor and severe forms of psychological aggression and physical‐aggression.
Notes	

Risk of bias table

**Table:** 

Bias	Authors' judgment	Support for judgment
Random sequence generation (selection bias)	Low risk	“Couples were randomly assigned to condition using a computer‐generated randomization list”
Allocation concealment (selection bias)	Unclear risk	“Participants were blind to condition, but the experimenter was not.” There is unclear reason to suspect that the enrolling investigator or the participant had knowledge of the forthcoming allocation.
Blinding of participants and personnel (performance bias)	High risk	“Participants were blind to condition, but the experimenter was not.”
Blinding of outcome assessment (detection bias)	High risk	No blinding of outcome assessment reported.
Incomplete outcome data (attrition bias)	Low risk	“Actor Partner Interdependence Model with treatment effects to analyze the obtained dyadic data. Intention‐to‐treat analysis, and full information maximum likelihood (FIML) estimation was used to accommodate missing data.”
Selective reporting (reporting bias)	Low risk	The authors did not selectively report their findings. For example, they state “The observed percentage change in expected counts for the aggression models likely overestimate the positive effect of ePREP given our sample size and that physical aggression was a rare occurrence; we attempted to mitigate this limitation by using appropriate analyses for modeling rare events.”
Other bias	Unclear risk	

Constantino et al. (2015)

**Table:** 

Methods	Three arm Randomized controlled trial: Online (ONL), Face‐to‐Face (FTF), and Waitlist Control (WC). A sequential, transformative mixed‐methods design was used.
Participants	32 adult female participants who were 45.2% Asian, 32.3% White, and 22.5% Black
Interventions	HELPP (Health, Education on Safety, and Legal Support and Resources in IPV Participant Preferred) intervention among IPV survivors. Online (ONL) HELPP. The ONL intervention consisted of six modules delivered by e‐mail once a week for 6 weeks. Face‐to‐Face (FTF) HELPP. The FTF intervention consisted of six modules and was given to each participant once a week for 6 weeks. Six HELPP modules, 1 week at a time to each participant (i.e., via e‐mail for ONL and in‐person for FTF).
Outcomes	Anxiety, depression, anger, personal, and social support
Notes	

Risk of bias table

**Table:** 

Bias	Authors' judgment	Support for judgment
Random sequence generation (selection bias)	Low risk	“Participants were randomly assigned to one of three study groups by permuted block randomization.”
Allocation concealment (selection bias)	Low risk	There is no reason to suspect that the enrolling investigator or the participant had knowledge of the forthcoming allocation. “The designation of numbers for intervention conditions (i.e., 1 = ONL, 2 = FTF, and 3 = WLC) were concealed from data collectors and the statistician. The sociodemographic characteristics of the participants did not significantly differ by group assignment.”
Blinding of participants and personnel (performance bias)	Unclear risk	No blinding of participants and personnel were reported. “[Participants were] informed that [they] would be notified by e‐mail and/or telephone call regarding the intervention group in which she was randomly placed.”
Blinding of outcome assessment (detection bias)	Low risk	“The designation of numbers for intervention conditions (i.e., 1 = ONL, 2 = FTF, and 3 = WLC) were concealed from data collectors and the statistician.”
Incomplete outcome data (attrition bias)	Low risk	“Almost 100% retention after randomization.”
Selective reporting (reporting bias)	Unclear risk	Small sample size.
Other bias	Unclear risk	

Decker et al. (2020)

**Table:** 

Methods	Randomized, controlled, participant‐blinded superiority trial
Participants	352 Kenyan women (*n* = 177 intervention, *n* = 175 control) enrolled and randomly assigned. Participants were predominantly married (84.94%) and cohabitating with their partner (85.90%), with a mean age of 26.52 years old (SD = 4.70) and 2.06 children (SD = 1.14). Slightly more than half (51.28%) had completed primary school or less and the vast majority (94.23%) were unemployed.
Interventions	The myPlan Kenya app intervention—an adaptation of the myPlan app—a decision aid and safety planning intervention accessible via a mobile app and website (myPlanApp.org)
Outcomes	Primary: safety preparedness, decisional conflict, use and helpfulness of safety behaviors, and IPV exposure. Secondary: Resilience, depression, use of IPV support services, self‐blame, recognition of IPV, self‐efficacy, relationship quality and risk for severe/lethal violence.
Notes	

Risk of bias table

**Table:** 

Bias	Authors' judgment	Support for judgment
Random sequence generation (selection bias)	Low risk	“Participants were randomly assigned (1:1) to receive either myPlan Kenya intervention or a standard of care control condition, stratified by study site. Computerised blocked randomisation provided site‐level stratification within the myPlan Kenya app”
Allocation concealment (selection bias)	Unclear risk	“The randomisation sequence (concealed from study staff) was programmed into a secure tracking database, separate from the study website, by the study programmer, who had no participant contact.”
Blinding of participants and personnel (performance bias)	Low risk	“All participants and staff were blinded to intervention status with the exception of team members administering the intervention.”
Blinding of outcome assessment (detection bias)	High risk	No blinding to outcome assessment was reported. “Measures were vetted locally and piloted with CHVs before use, resulting in minor refinements to enhance readability.”
Incomplete outcome data (attrition bias)	Low risk	“The intent‐to‐treat, differences‐in‐differences approach used random effects logistic and linear regression models.”
Selective reporting (reporting bias)	Low risk	Negative findings were reported.
Other bias	Unclear risk	

Doss et al. (2016)

**Table:** 

Methods	Randomized controlled trial
Participants	300 heterosexual couples (*N* = 600 participants) participated; couples were generally representative of the US in terms of race, ethnicity, and education.
Interventions	OurRelationship program—a low‐cost, Web‐based format; In the program, couples' complete online activities and have four, 15‐min calls with project staff
Outcomes	Nine primary outcome measures (assessing four relationship and five individual functioning constructs). **Relationship outcomes**: relationship satisfaction, Positive Relationship and Negative Relationship Quality, Relationship Confidence. **Individual outcomes**: Depression, Anxiety, Perceived Health, Work Functioning, Quality of Life
Notes	

Risk of bias table

**Table:** 

Bias	Authors' judgment	Support for judgment
Random sequence generation (selection bias)	Low risk	“Using a random number generator, 151 couples were randomized into the web‐based intervention condition and 149 couples were randomized into the waitlist control condition. There were no significant between‐group differences at the pre‐treatment assessment on any of the 12 demographic variables or on any the eight demographic variables.”
Allocation concealment (selection bias)	High risk	Although, randomly assigned, no allocation concealment reported. There is reason to suspect that the enrolling investigator or the participant had knowledge of the forthcoming allocation.
Blinding of participants and personnel (performance bias)	Unclear risk	No blinding of participants and personnel reported.
Blinding of outcome assessment (detection bias)	Low risk	No blinding of outcome assessment reported.
Incomplete outcome data (attrition bias)	Unclear risk	“Missing data was unrelated to condition (*p* = 0.882) as well as to change in the eight dependent variables (all *p* > 0.15). All available data was included in all analyses.”
Selective reporting (reporting bias)	Low risk	The authors state “The effects of the program on positive relationship quality did not significantly differ by gender…Examinations of clinically significant change in relationship satisfaction revealed that 32% of participants were recovered by the end of the intervention, 25% were improved, 36% experienced no change, and 7% deteriorated.”
Other bias	Unclear risk	

Ford‐Gilboe et al. (2020)

**Table:** 

Methods	Double blind randomized controlled trial RCT
Participants	462 Canadian adult women
Interventions	Tailored, interactive online safety and health intervention (iCAN Plan 4 Safety).
Outcomes	Primary (depressive symptoms, PTSD symptoms) and secondary (helpfulness of safety actions, confidence in safety planning, mastery, social support, experiences of coercive control, and decisional conflict)
Notes	

Risk of bias table

**Table:** 

Bias	Authors' judgment	Support for judgment
Random sequence generation (selection bias)	Low risk	“Using 1:1 allocation, women were randomly assigned to receive iCAN, an interactive, tailored online safety and health intervention or a brief, static version that was not tailored (i.e., not personalized). To achieve balance in the sample across the study sites, a stratified block randomization scheme was used based on both the province of residence and whether the woman had children under 18 years living at home.”
Allocation concealment (selection bias)	Low risk	“The randomization algorithm was pre‐programmed into the study tracking database by the study programmer who had no contact with participants.” There is no reason to suspect that the enrolling investigator or the participant had knowledge of the forthcoming allocation.
Blinding of participants and personnel (performance bias)	Low risk	“Participants were not informed of their group assignment. The research team members other than the programmer (JC) and statistician (NP), were blind to group assignment until the final 12‐month surveys had been completed.”
Blinding of outcome assessment (detection bias)	Low risk	“The research team members other than the programmer (JC) and statistician (NP), were blind to group assignment until the final 12‐month surveys had been completed.”
Incomplete outcome data (attrition bias)	Low risk	“Retention was 89.6, 87.0, and 87.0% at 3‐, 6‐, and 12‐months, respectively for the tailored group. In the non‐tailored group, retention was 91.8, 91.3, and 90.5% at 3‐, 6‐, and 12‐months, respectively. Attrition across all time points was small and largely due to losing contact with women.”
Selective reporting (reporting bias)	Low risk	Clinical trial prospectively registered. Also non‐significant findings reported, for instance the authors say “Both groups improved significantly over time on the primary outcomes of depression (*p* < 0.001) and PTSD symptoms (*p* < 0.001). However, the change over time did not differ between the tailored and non‐tailored groups for either depression (*p* = 0.598) or PTSD (*p* = 0.269). A similar pattern was found for the secondary outcomes.”
Other bias	Unclear risk	

Gilbert et al. (2016)

**Table:** 

Methods	Three‐arm randomized controlled trial
Participants	191 substance‐using women in probation and community court sites in New York City. A total of 103 participants were assigned to Computerized WORTH, 101 to Traditional WORTH, and 102 to Wellness Promotion.
Interventions	Three‐arm study of a computerized HIV and IPV prevention trial: (1) 4 group sessions intervention with computerized self‐paced IPV prevention modules (Computerized Women on the Road to Health [WORTH]), (2) traditional HIV and IPV prevention intervention group covering the same HIV and IPV content as Computerized WORTH without computers (Traditional WORTH), and (3) a Wellness Promotion control group.
Outcomes	Physical, injurious, and sexual IPV victimization in the previous 6 months at 12‐month follow‐up
Notes	

Risk of bias table

**Table:** 

Bias	Authors' judgment	Support for judgment
Random sequence generation (selection bias)	Low risk	“A study investigator randomly assigned groups of 4 to 9 women to 1 of 3 study conditions; a computer‐generated randomization algorithm was designed to balance the number of women per study arm via an adaptive, biased‐coin procedure.”
Allocation concealment (selection bias)	Low risk	There is no reason to suspect that the enrolling investigator or the participant had knowledge of the forthcoming allocation. “Investigators were masked to treatment assignment until the final 12‐month follow‐up assessment was completed in April 2013.”
Blinding of participants and personnel (performance bias)	Unclear risk	“Investigators were masked to treatment assignment until the final 12‐month follow‐up assessment was completed in April 2013. Data were locked in September 2013, after which study arms were unmasked.”
Blinding of outcome assessment (detection bias)	High risk	No blinding of outcome assessment reported.
Incomplete outcome data (attrition bias)	Low risk	“Consistent with the intent‐to‐treat approach, we estimated intervention effects by analyzing participant responses based on their experimental assignment. Because some missing data were the result of loss to follow‐up at postintervention assessments, we used all available data at any follow‐up visit in the statistical models. Attrition analyses, which compared sociodemographic characteristics of those who completed all follow‐up assessments (completers) with those who missed 1 or more follow‐up assessments (noncompleters), identified that completers on average were older (42 vs. 39 years) and less likely to report homelessness (8% vs. 18%).”
Selective reporting (reporting bias)	Low risk	No selective reporting suspected.
Other bias	Unclear risk	

Glass et al. (2017)

**Table:** 

Methods	Multistate, community‐based longitudinal Randomized controlled trial
Participants	Currently abused Spanish‐ or English‐speaking women (*N* = 720).
Interventions	Tailored, Internet‐based safety decision aid (included priority‐setting activities, risk assessment, and tailored feedback and safety plans).
Outcomes	Decisional conflict, safety behaviors, and repeat IPV; secondary: depression and PTSD.
Notes	

Risk of bias table

**Table:** 

Bias	Authors' judgment	Support for judgment
Random sequence generation (selection bias)	Low risk	Study was a Multistate, community‐based longitudinal RCT with one‐to‐one allocation ratio and blocked randomization.
Allocation concealment (selection bias)	Low risk	“The randomization sequence (concealed from research assistants [RAs]) was programmed into a secure tracking database separate from the study website by the study programmer, who had no participant contact.” There is no reason to suspect that the enrolling investigator or the participant had knowledge of the forthcoming allocation.
Blinding of participants and personnel (performance bias)	Unclear risk	Participants were blinded to group assignment.
Blinding of outcome assessment (detection bias)	High risk	No mention of blinding of data statistician.
Incomplete outcome data (attrition bias)	Low risk	Analyses were conducted using an intent‐to‐treat approach including generalized estimating equations to test for differences in change over time between groups.
Selective reporting (reporting bias)	Low risk	At 12 months, there were no significant group differences in IPV, depression, or post‐traumatic stress disorder.
Other bias	Unclear risk	

Glass et al. (2021)

**Table:** 

Methods	Longitudinal Randomized Control Trial
Participants	Three hundred forty‐six women (175 intervention, 171 control) from 41 colleges/universities in Oregon and Maryland
Interventions	The myPlan app is an interactive decision aid and safety planning intervention that is free and accessible via a mobile app and website (myPlanApp.org)
Outcomes	Four measures of intimate partner violence (IPV; Composite Abuse Scale [CAS], TBI‐related IPV, digital abuse, reproductive coercion [RC]), depression, and suicide risk.
Notes	

Risk of bias table

**Table:** 

Bias	Authors' judgment	Support for judgment
Random sequence generation (selection bias)	Low risk	“An automated algorithm randomly assigned enrolled participants to the intervention or control group. The randomization was stratified on state of residence, having children (child/no child in the home), and type of college/university.”
Allocation concealment (selection bias)	Unclear risk	Although no allocation concealment was reported, the authors state “Following enrollment and randomization, an automated email was sent to the participant's safe email address containing instructions and links for safely accessing myPlan or usual safety planning.” There is some reason to suspect that the enrolling investigator or the participant had knowledge of the forthcoming allocation.
Blinding of participants and personnel (performance bias)	High risk	It is likely personnel were privy to group assignments, given the authors state in their published protocol: “Participants access the study website or App to complete data collection. Participants can contact the RA if they have technical problems with the website or App, questions or concerns about the study, or need help finding someone safe to talk to. “
Blinding of outcome assessment (detection bias)	High risk	No blinding to outcome assessment was reported.
Incomplete outcome data (attrition bias)	Low risk	“Analyses were conducted using an intention‐to‐treat approach. All women were included in the main analyses as these models do not require complete data at each time point.”
Selective reporting (reporting bias)	Low risk	Negative findings were reported. A prior protocol was published in 2015. “Women in both groups reported increases in the percent of safety behaviors used and helpful, as well as decreases in decisional conflict, misuse of alcohol, symptoms of depression, and IPV, however the changes over time were not significantly different by group. While these results do not support the effectiveness of myPlan over usual safety planning on all outcomes, they support the use of technology‐based safety planning for young women that have recently (past 6 months) experienced IPV by a partner or ex‐partner.”
Other bias	Unclear risk	

Hegarty et al. (2019)

**Table:** 

Methods	Two‐group pragmatic randomized controlled trial, randomly assigned
Participants	Women aged 16–50 years currently residing in Australia. 422 eligible participants were randomly allocated to the intervention group (227 patients) or control group (195 patients)
Interventions	An online healthy relationship tool/website and safety decision aid for women experiencing intimate partner violence (I‐DECIDE)
Outcomes	Self‐efficacy, depression, fear of partner, and number of helpful behaviors for safety and wellbeing, and cost‐effectiveness
Notes	

Risk of bias table

**Table:** 

Bias	Authors' judgment	Support for judgment
Random sequence generation (selection bias)	Low risk	“Participants were randomly assigned (1:1) by computer to receive either the intervention or control website. Once women were enrolled in the study, they were randomly assigned by computer to the intervention or control group. An automated, computerised algorithm for simple 1:1 randomisation was used, with no stratification”
Allocation concealment (selection bias)	Unclear risk	“There was no way for women to tell which group they had been allocated to. Women were masked to treatment allocation, although it is possible that some may have guessed which website they were receiving.” There is some reason to suspect that the enrolling investigator or the participant had knowledge of the forthcoming allocation.
Blinding of participants and personnel (performance bias)	Low risk	“Women were masked to treatment allocation, although it is possible that some may have guessed which website they were receiving. All the research team were masked to participant allocation until after analysis of the 12‐month data.”
Blinding of outcome assessment (detection bias)	High risk	No blinding to outcome assessment was reported.
Incomplete outcome data (attrition bias)	Low risk	“Both the main and imputed data analyses were done according to intention‐to‐treat principles.”
Selective reporting (reporting bias)	Low risk	“Baseline characteristics of participants were similar between the intervention and control groups.” Clinical trial prospectively registered
Other bias	Unclear risk	

Koziol‐McLain et al. (2018)

**Table:** 

Methods	Fully automated Web‐based two‐arm parallel randomized controlled trial (RCT) in a general population of New Zealand women who had experienced IPV in the past 6 months
Participants	412 general population of New Zealand women
Interventions	Individualized Web‐based *isafe* decision aid—password‐protected intervention website (safety priority setting, danger assessment, and tailored action plan components) or control website (standard, non‐individualized information).
Outcomes	Self‐reported mental health (Center for Epidemiologic Studies Depression Scale‐Revised, CESD‐R) and IPV exposure (Severity of Violence Against Women Scale, SVAWS) at 12‐month follow‐up.
Notes	

Risk of bias table

**Table:** 

Bias	Authors' judgment	Support for judgment
Random sequence generation (selection bias)	Low risk	“Computer‐generated randomization was based on a minimization scheme with stratification by severity of violence and children. Two stratification factors (severity of violence and children) and two random factors each with two equiprobable levels were used to achieve the minimization.”
Allocation concealment (selection bias)	Low risk	“The participant's allocation was kept secret from herself and the study team in New Zealand.” There is no reason to suspect that the enrolling investigator or the participant had knowledge of the forthcoming allocation.
Blinding of participants and personnel (performance bias)	Low risk	“The participant's allocation was kept secret from herself and the study team in New Zealand.”
Blinding of outcome assessment (detection bias)	Low risk	“A blind review, absent any information regarding allocation, was undertaken for each outcome for an assessment of missingness, a visual assessment of residual normality, an assessment of the covariance structure of the repeated measures, and an assessment of candidate covariates (ethnicity, children, paid employment, and age group) to include in the final analysis.”
Incomplete outcome data (attrition bias)	Low risk	“Intention‐to‐treat (ITT) analysis. Missing data at baseline was imputed using the mode of the variable in the observed ITT values. Missingness in both primary outcomes was found to be significantly related to the last observed value of the outcome in question, to the assessment time being at 12 months in the case of CESD‐R but to no other baseline variable, indicating that adjusting for baseline and fitting the available repeated measures with a suitable (non‐diagonal) covariance structure appropriately removed the risk of bias from missingness under a missing at random assumption.”
Selective reporting (reporting bias)	Low risk	Clinical trial prospectively registered
Other bias	Unclear risk	

Littleton et al. (2016)

**Table:** 

Methods	Randomized controlled trial (RCT)
Participants	Eighty‐seven college women with rape‐related PTSD were randomized to complete the interactive program (*n* = 46) or a psycho‐educational self‐help website (*n* = 41)
Interventions	From Survivor to Thriver program, an interactive, online therapist‐facilitated cognitive‐behavioral program for rape‐related PTSD. The From Survivor to Thriver Program consisted of nine program modules. The program was designed to be completed sequentially, with participants completing one module at a time.
Outcomes	Rape‐related PTSD symptoms, working alliance with their therapist
Notes	

Risk of bias table

**Table:** 

Bias	Authors' judgment	Support for judgment
Random sequence generation (selection bias)	Low risk	“Participants were randomized to the interactive program or psychoeducational website based on a computerized coin flip.”
Allocation concealment (selection bias)	Unclear risk	No allocation concealment was reported. There is some reason to suspect that the enrolling investigator or the participant had knowledge of the forthcoming allocation.
Blinding of participants and personnel (performance bias)	High risk	“Additionally, given the nature of the trial, individuals completing pre‐, post‐ and follow‐up assessments were not blind to participants' intervention condition.”
Blinding of outcome assessment (detection bias)	High risk	No blinding of outcome assessment reported.
Incomplete outcome data (attrition bias)	Low risk	“Analyses were conducted on individuals who initiated the assigned programs and provided data at post‐test assessments. The use of “initiators” in our analysis allowed us to investigate the effect of the program for both groups of participants. In addition, estimates of change from pre‐test to post‐test and follow‐up were estimated for all participants assigned to a program, an Intent‐to‐Treat (ITT) model.”
Selective reporting (reporting bias)	Low risk	Some negative findings were reported. The authors state “Two women who completed the interactive program reported clinically significant increases in depression at post‐treatment, one of whom also reported a clinically significant increase in anxiety. Notably, both of these women experienced the death of an immediate family member (mother, grandfather) while completing the intervention, so it is possible that these losses led to their increased symptomology. Three additional women also reported clinically significant increases in anxiety, although two of these women reported clinically significant decreases in PTSD symptomatology.”
Other bias	Unclear risk	

Stevens et al. (2015)

**Table:** 

Methods	Randomized‐controlled trial of telephone support services (TSS) versus enhanced usual care (EUC) for women who had reported intimate partner violence (IPV) within the past year during a visit to a pediatric emergency department.
Participants	Three hundred women, ages 18 years and above were recruited
Interventions	Telephone support services (TSS)—assessment phase, implementation phase, monitoring phase, secondary implementation phase, and termination phase.
Outcomes	IPV victimization, feelings of chronic vulnerability to perpetrator, depressive symptoms, and PTSD
Notes	

Risk of bias table

**Table:** 

Bias	Authors' judgment	Support for judgment
Random sequence generation (selection bias)	Low risk	“Assignment to condition was based on a computer‐generated random number table. One hundred twenty‐nine participants were randomly assigned to the intervention condition (TSS), and 124 participants were randomly assigned to the control condition (EUC).”
Allocation concealment (selection bias)	Unclear risk	Unclear if allocation was blinded or followed any concealment protocol. There is some reason to suspect that the enrolling investigator or the participant had knowledge of the forthcoming allocation.
Blinding of participants and personnel (performance bias)	Low risk	“Numerous steps were taken to keep the research assistants unaware of study condition. First, the interventionists made calls in separate offices from the research assistants. Second, the research assistants were not given access to files containing condition assignments until the end of data collection from participants. Third, the interventionists called participants in both study conditions so that a participant's infrequent mention to a research assistant of a conversation with a study nurse would not automatically reveal study condition.”
Blinding of outcome assessment (detection bias)	High risk	No blinding to outcome assessment reported.
Incomplete outcome data (attrition bias)	Unclear risk	“There was a 76% retention rate for the intervention group from baseline to 3 months (*n* = 98), and a 77% retention rate for the control group from baseline to 3 months (*n* = 95). There was a 70% retention rate for the intervention group at 6 months (*n* = 90), and a 72% retention rate for the control group at 6 months (*n* = 93). There was no significant difference in retention between the two conditions (*χ* ^2^ = 0.155, *p* = 0.92).” Although, no report of measures taken to mitigate attrition bias.
Selective reporting (reporting bias)	Low risk	“Specifically, the TSS and EUC groups did not differ on any outcome variable, including IPV victimization, feelings of chronic vulnerability to a perpetrator, depressive symptoms, and PTSD symptoms. This lack of intervention effect was generalizable across both time (mid‐intervention vs. post‐intervention) and across both nurse interventionists. The lack of dose effect in the present study must be interpreted with caution, given the low intensity of assistance received by all TSS recipients.”
Other bias	Unclear risk	

Tiwari et al. (2010)

**Table:** 

Methods	Assessor‐blinded randomized controlled trial (RCT).
Participants	200 community‐dwelling, abused Chinese women were randomly assigned to the intervention (*n* = 100) or control (*n* = 100) group. The former group received a 12‐week advocacy intervention, whereas the latter received usual community services
Interventions	Telephone intervention to improve the mental health of community‐dwelling women abused by their intimate partners: a randomized controlled trial 18 years or older with a history of IPV.
Outcomes	Depression was the primary outcome measure. The secondary outcome measures were perceived social support, health‐related quality of life, IPV, safety‐promoting behaviors, and utilization of health services
Notes	

Risk of bias table

**Table:** 

Bias	Authors' judgment	Support for judgment
Random sequence generation (selection bias)	Low risk	“Participants were randomized (1:1) to the intervention or control group according to a list of random permutations prepared by computer‐generated blocked randomization performed by a research staff member who had not been involved in participant recruitment.”
Allocation concealment (selection bias)	Low risk	“The block size was kept secure by the randomizer, and the order of allocation was centrally controlled to avoid any bias in selection. The allocation sequence was concealed in opaque envelopes.” There is no reason to suspect that the enrolling investigator or the participant had knowledge of the forthcoming allocation.
Blinding of participants and personnel (performance bias)	Unclear risk	The allocation sequence was concealed in opaque envelopes. At the time of randomization, the research assistant who had successfully recruited a participant called the site investigator, who then opened the envelope containing the group assignment. To ensure random assignment, no detail was provided to the site investigator about the identity of the participant.
Assessors were not involved in the design of the study, did not know the study hypotheses, and were blinded to group assignment.		
Blinding of outcome assessment (detection bias)	Low risk	“Assessors were not involved in the design of the study, did not know the study hypotheses, and were blinded to group assignment.”
Incomplete outcome data (attrition bias)	Unclear risk	No subjects were lost to follow up.
Selective reporting (reporting bias)	Low risk	“In this randomized clinical trial of an advocacy intervention for community‐dwelling abused Chinese women, the intervention did not result in a clinically meaningful improvement in depressive symptoms.”
Other bias	Unclear risk	

Zlotnick et al. (2019)

**Table:** 

Methods	Two‐group, randomized controlled trial
Participants	About 53 currently pregnant or within 6 months perinatal women (currently pregnant or within 6 months postpartum) seeking mental health treatment at an urban hospital‐based behavioral health clinic, who were 18 years of age or older, English‐speaking, and reported experiencing IPV in the past 12 months were eligible and recruited into the study.
Interventions	Brief, motivational computer‐based intervention, SURE (Strength for U in Relationship Empowerment), for perinatal women with IPV seeking mental health treatment
Outcomes	IPV outcomes (Severe combined abuse, physical, emotion abuse, harassment), as well as feasibility and acceptability of intervention
Notes	

Risk of bias table

**Table:** 

Bias	Authors' judgment	Support for judgment
Random sequence generation (selection bias)	Low risk	“After completion of the baseline assessment, the (computer) narrator flipped a coin, and participants were randomized into the control or SURE intervention.”
Allocation concealment (selection bias)	High risk	Allocation done, but no allocation concealment reported. There is reason to suspect that the enrolling investigator or the participant had knowledge of the forthcoming allocation.
Blinding of participants and personnel (performance bias)	High risk	No blinding of participants and personnel reported.
Blinding of outcome assessment (detection bias)	High risk	No blinding of outcome assessment reported.
Incomplete outcome data (attrition bias)	Low risk	“Our completion and retention rates were very high—92% of the women completed the 4‐month follow‐up assessment—demonstrating our ability to recruit and retain a high‐risk, and racially and ethnically diverse sample.” The same number allocated were included in the data analysis, although some two participants in each group were lost to follow‐up.
Selective reporting (reporting bias)	Low risk	Some negative findings reported. The authors state: “There were no significant differences between the two conditions on the IPV at risk scores (i.e., WAST scores) at screening with SURE participants obtaining a mean of 6.18 (SD = 1.79) and the control a mean score of 6.04 (SD = 2.05) (*t*‐test *p* = .794).” Additionally, a protocol was registered in a clinical trial registry.
Other bias	Unclear risk	

*Footnotes*

**Characteristics of excluded studies**

**Table:** 

Alhusen et al., 2015	
**Reason for exclusion**	Qualitative study
Bacchus et al., 2016	
**Reason for exclusion**	Qualitative Study
Bloom et al., 2014	
**Reason for exclusion**	Qualitative study
Bloom et al., 2016	
**Reason for exclusion**	Wrong outcomes
Bowen et al., 2014	
**Reason for exclusion**	Wrong outcomes
Choo et al., 2016	
**Reason for exclusion**	Wrong outcomes
Constantino et al., 2007	
**Reason for exclusion**	Feasibility study
Debnam & Kumodzi, 2019	
**Reason for exclusion**	Qualitative study
Draucker et al., 2019	
**Reason for exclusion**	Pre‐post study
Eden et al., 2015	
**Reason for exclusion**	Wrong outcomes
Fiorillo et al., 2017	
**Reason for exclusion**	Pre‐post study
Ford‐Gilboe et al., 2017	
**Reason for exclusion**	Protocol
Gilbert et al., 2015	
**Reason for exclusion**	Wrong outcome
Gilbert et al., 2017	
**Reason for exclusion**	Feasibility study, wrong outcomes
Glass et al., 2010	
**Reason for exclusion**	Wrong outcome
Gray et al., 2015	
**Reason for exclusion**	Pre‐post study
Christina Hassija & Gray, 2011	
**Reason for exclusion**	Pre‐post study
Humphreys et al., 2011	
**Reason for exclusion**	Wrong outcome
Klevens et al., 2012	
**Reason for exclusion**	Wrong outcome
Koziol‐McLain et al., 2016	
**Reason for exclusion**	Recruitment and Engagement study
Linde et al., 2020	
**Reason for exclusion**	Systematic Review and Meta‐Analysis
Lindsay et al., 2013	
**Reason for exclusion**	Wrong outcome
MacMillan et al., 2006	
**Reason for exclusion**	Wrong study duration
McFarlane et al., 2002	
**Reason for exclusion**	Wrong study duration, wrong outcomes
Morr and Layal, 2020	
**Reason for exclusion**	Systematic review
Ranney et al., 2013	
**Reason for exclusion**	Wrong outcomes
Sabri et al., 2019	
**Reason for exclusion**	Protocol
Sargent et al., 2016	
**Reason for exclusion**	Wrong outcome
Tarzia et al., 2017	
**Reason for exclusion**	Qualitative study
Tarzia et al., 2018	
**Reason for exclusion**	Qualitative study
Thomas et al., 2005	
**Reason for exclusion**	Wrong study duration
Young‐Hauser et al., 2014	
**Reason for exclusion**	Qualitative study

## SUMMARY OF FINDINGS TABLES

 

## DATA AND ANALYSES

1 Meta‐analysis of included studies

**Table:** 

Outcome or subgroup	Studies	Participants	Statistical method	Effect estimate
1.1 Depression Outcome	14	13,948	Std. Mean Difference (IV, Fixed, 95% CI)	−0.04 [−0.08, −0.01]
1.1.1 Depression, 10+ months	6	2244	Std. Mean Difference (IV, Fixed, 95% CI)	−0.07 [−0.15, 0.02]
1.1.2 Depression, 10+ months (Outlier removed)	5	2167	Std. Mean Difference (IV, Fixed, 95% CI)	−0.03 [−0.11, 0.05]
1.1.3 Depression, 3–9 months	7	2611	Std. Mean Difference (IV, Fixed, 95% CI)	0.06 [−0.01, 0.14]
1.1.4 Depression, 3–9 months (Outlier removed)	6	2261	Std. Mean Difference (IV, Fixed, 95% CI)	−0.05 [−0.13, 0.03]
1.1.5 Depression, 0–3 months	10	2343	Std. Mean Difference (IV, Fixed, 95% CI)	−0.10 [−0.18, −0.01]
1.1.6 Depression, 0–3 months (Outlier Removed)	9	2322	Std. Mean Difference (IV, Fixed, 95% CI)	−0.08 [−0.17, −0.00]
1.2 Anxiety Outcome	4	714	Std. Mean Difference (IV, Fixed, 95% CI)	−0.27 [−0.42, −0.13]
1.3 PTSD	4	1428	Std. Mean Difference (IV, Fixed, 95% CI)	−0.04 [−0.14, 0.06]
1.3.1 PTSD Outcome	4	1428	Std. Mean Difference (IV, Fixed, 95% CI)	−0.04 [−0.14, 0.06]
1.4 Physical Victimization, 0–6 months	6	883	Std. Mean Difference (IV, Fixed, 95% CI)	−0.45 [−0.59, −0.32]
1.5 Physical Victimization, 0–6 months (Outlier Removed)	5	560	Std. Mean Difference (IV, Fixed, 95% CI)	−0.22 [−0.38, −0.05]
1.6 Physical Victimization, >6 months	6	1540	Std. Mean Difference (IV, Fixed, 95% CI)	−0.05 [−0.15, 0.05]
1.7 Physical Victimization, >6 months (Outlier Removed)	4	1107	Std. Mean Difference (IV, Fixed, 95% CI)	0.04 [−0.08, 0.15]
1.8 Psychological Victimization, 0–6 months	6	952	Std. Mean Difference (IV, Fixed, 95% CI)	−0.34 [−0.47, −0.20]
1.9 Psychological Victimization, 0–6 months (Outlier Removed)	4	609	Std. Mean Difference (IV, Fixed, 95% CI)	0.06 [−0.10, 0.22]
1.10 Psychological Victimization, >6 months	5	1357	Std. Mean Difference (IV, Fixed, 95% CI)	−0.29 [−0.39, −0.18]
1.11 Psychological Victimization, >6 months (Outlier Removed)	4	1000	Std. Mean Difference (IV, Fixed, 95% CI)	0.00 [−0.12, 0.13]
1.12 Sexual Victimization, 6–9 months	4	1402	Std. Mean Difference (IV, Fixed, 95% CI)	0.32 [0.21, 0.43]
1.13 Sexual Victimization, 6–9 months (Outlier Removed)	3	1056	Std. Mean Difference (IV, Fixed, 95% CI)	−0.02 [−0.14, 0.11]

## Supporting information

Supporting information.
