# Supplementary material for: Technology‐based and digital interventions for intimate partner violence: A systematic review and meta‐analysis
Source: Campbell Syst Rev. 2022 Aug 27;18(3):e1271. doi: 10.1002/cl2.1271 (PMC9419475; doi:10.1002/cl2.1271)
Supplement: Supplementary file 1 — Supporting information. [file CL2-18-e1271-s001.docx]

# Appendices

## 1 Search Strategy

**An example of an aggregated search using boolean configuration for "technology" AND "intimate partner violence" is included in Appendix D**

| **In traditional and grey databases** | | | |
| --- | --- | --- | --- |
|  | **Technology** |  | **Intimate partner violence** |
| **Search terms** | (mHealth OR mobile apps OR eHealth OR mobile applications OR interactive mobile application OR apps OR "mobile app" OR electronic interventions OR safety app OR decision aid OR mobile intervention OR smartphone OR smartphone apps OR smartphone-based app OR smartphone-delivered OR mobile-delivered OR technology-mediated OR internet-based OR web-based OR computer-based OR computerized OR  electronic OR technology-based OR "use of technology" OR m-Health OR e-Health OR social network OR social media OR mobile phone OR mobile device OR Virtual communit* OR virtual reality OR Twitter OR Facebook OR WhatsApp OR WeChat “mobile health” OR “mobile care” OR “m Health” OR “mobile phone” OR “mobile device” OR “mobile technology” OR “mobile communication” OR “mobile telecommunication” OR “mobile app” OR “mobile application” OR “mobile tool” OR “mobile messaging” OR “mobile electronic device” OR “mobile telephone” OR “mobile phones” OR “mobile devices” OR “mobile technologies” OR “mobile communications” OR “mobile telecommunications” OR “mobile apps” OR “mobile applications” OR “mobile tools” OR “mobile messages” OR “mobile electronic devices” OR “mobile telephones” OR “mobile intervention” OR “mobile interventions” OR “mobile delivered” OR “mobile delivery OR  information, communication technology OR  ICT OR email) | AND | ("Intimate Partner Violence"[Mesh] OR "partner violence" OR "partner abuse" OR "dating violence" OR "dating abuse" OR dating violence OR partner abuse OR adolescent dating violence OR OR stalking OR assault OR coercion OR "digital abuse" OR rape OR battered women OR "domestic abuse” OR “wife abuse" OR "Spouse Abuse”[Mesh] OR “Domestic Violence”[Mesh:noexp] OR intimate partner violence[tiab] OR domestic violence[tiab] OR dating violence[tiab] OR partner violence[tiab] OR domestic abuse[tiab] OR partner abuse[tiab] OR (Abuse[tiab] OR abusive[tiab] OR abused[tiab] OR battered[tiab] OR battering[tiab] OR violent[tiab] OR violence[tiab] OR assaultive[tiab]) |

### Search strategies in databases

**PubMed (two search streams)**

| Time |  | Query | Results | Yield |
| --- | --- | --- | --- | --- |
| T1 | IPV, before March 21, 2019 | (mHealth OR mobile apps OR eHealth OR mobile applications OR Interactive Mobile Application OR apps OR Mobile app or electronic interventions OR safety app OR decision aid OR mobile intervention OR smartphone OR smartphone apps OR smartphone-based app OR smartphone-delivered OR mobile-delivered OR technology-mediated OR) AND (adolescent OR teenagers OR teens OR youth OR Young people OR juvenile OR pubescent OR young OR juvenile) AND (teen dating violence OR youth violence OR dating violence OR adolescent dating violence OR abuse OR Relationship Violence or Abuse OR Stalking OR Sexting  OR assault OR coercion OR digital abuse OR rape)  Sort by: Best match; From 2009/01/01 to 2019/12/31 | **2194** | **23** |
| T2 | General IPV, after March 21, 2019 | (mHealth OR mobile apps OR eHealth OR mobile applications OR Interactive Mobile Application OR apps OR Mobile app or electronic interventions OR safety app OR decision aid OR mobile intervention OR "smartphone" OR "smartphone apps" OR "smartphone-based" app OR smartphone-delivered OR mobile-delivered OR technology-mediated OR) AND ("Intimate Partner Violence"[Mesh] OR "partner violence" OR "partner abuse" OR "dating violence" OR "date rape" OR "dating abuse" OR dating violence OR partner abuse OR youth violence OR adolescent dating violence OR abuse OR Relationship Violence or Abuse OR Stalking OR Sexting OR assault OR coercion OR digital abuse OR rape) | **100** | NA |
| T3 | March 26, 2019 | (((mHealth OR mobile apps OR eHealth OR mobile applications OR Interactive Mobile Application OR apps OR Mobile app or electronic interventions OR safety app OR decision aid OR mobile intervention OR "smartphone" OR "smartphone apps" OR "smartphone-based" app OR smartphone-delivered OR mobile-delivered OR technology-mediated OR) AND ("Intimate Partner Violence"[Mesh] OR "partner violence" OR "partner abuse" OR "dating violence" OR "date rape" OR "dating abuse" OR dating violence OR partner abuse OR youth violence OR adolescent dating violence OR abuse OR Relationship Violence or Abuse OR Stalking OR Sexting OR assault OR coercion OR digital abuse OR rape) | **195** | **7** |
| T4 | March 26, 2019 | ("mobile applications" [Mesh]) AND ("Intimate Partner Violence"[Mesh] OR "partner violence" OR "partner abuse" OR "dating violence" OR "dating abuse" OR partner abuse OR "teen dating violence" OR abuse OR Relationship Violence or Abuse OR Stalking OR "sexual coercion" OR digital abuse OR rape)    Filters activated: Publication date from 2009/01/01 to 2019/12/31, MEDLINE, Nursing journals. Clear all to show 79 items | **78**    . | **3** |

**Google Scholar**

| Query | Results |
| --- | --- |
| (mHealth OR mobile apps OR eHealth OR mobile applications OR apps) AND ("Intimate Partner Violence"[Mesh] OR "partner violence" OR "partner abuse" OR "dating violence" OR "date rape" OR "dating abuse" OR dating violence OR partner abuse OR youth violence OR adolescent dating violence OR abuse OR Relationship Violence or Abuse OR Stalking OR Sexting OR assault OR coercion OR digital abuse OR rape) | 182 |

**Journal of Medical Internet Research Publications**

| No. |  | Query | Results |
| --- | --- | --- | --- |
| #1 | Journal of Medical Internet Research | mobile apps OR smartphone OR mHealth AND "intimate partner violence" OR dating violence | 132 |
| #2 | JMIR mHealth and uHealth | mobile apps OR smartphone OR mHealth AND "intimate partner violence" OR dating violence | 52 |
| #3 | JMIR Public Health and Surveillance | mobile apps OR smartphone OR mHealth AND "intimate partner violence" OR dating violence | 33 |

**Journal of Medical Internet Research Publications (part 2, May 6, 2019)**

| No. |  | Query | Results |
| --- | --- | --- | --- |
| #1 | Field 1 | intimate partner violence OR dating violence OR Abuse | 858 |
| #2 | Field 2 | mobile apps OR smartphone OR mHealth |  |

**CINAHL – April 12, 2019**

| **#** | **Query** | **Limiters/Expanders** | **Results** |
| --- | --- | --- | --- |
| S1 | (MH "Intimate Partner Violence") OR (MH "Domestic Violence") OR (MH "Gender-Based Violence") OR (MH "Dating Violence") | Search modes - Boolean/Phrase | 16,144 |
| S2 | (MH "Telehealth+") OR (MH "Mobile Applications") OR (MH "World Wide Web+") OR (MH "Early Intervention") OR (MM "Intervention Trials") | Search modes - Boolean/Phrase | 113,613 |
| S3 | s1 and s2 | Limiters - Research Article; Journal Subset: Peer Reviewed; Peer Reviewed; English Language; Human; Published Date: 20090101-20191231 | 58 |

**PsycINFO + PsycARTICLES**

| **Search ID#** | **Query** | **Limiters/Expanders** |
| --- | --- | --- |
| S1 | "intimate partner violence" or domestic violence or partner abuse or intimate partner aggression or ipv | Boolean/Phrase |
| S2 | (mhealth or mobile or ehealth or internet or smartphone or online or web-based or web based or mobile application or app or cellphone or cell phone or telehealth or tablet or technology or network ) AND ( mhealth or mobile health or m-health or mobile app or mobile application or smartphone application or app or apps or telephone ) AND (digital interventions or online interventions or ehealth interventions | Boolean/Phrase |
| S3 | digital interventions or online interventions or ehealth interventions | Boolean/Phrase |
| S4 | randomised controlled trial or randomized controlled trial or rct | Boolean/Phrase |
| S5 | feasibility and acceptability | Boolean/Phrase |
| S6 | ((((mhealth or mobile or ehealth or internet or smartphone or online or web-based or web based or mobile application or app or cellphone or cell phone or telehealth or tablet or technology or network) AND (mhealth or mobile health or m-health or mobile app or mobile application or smartphone application or app or apps)) AND (S1 AND S2 AND S3 AND S4)) | **Limiters** - Full Text; Year of Publication: 2009-2019; Population Group: Human  **Expanders** - Apply related words  **Search modes** - SmartText Searching |

**PsycINFO– April 12, 2019**

| **Search ID#** | **Query** | **Limiters/Expanders** | **Results** |
| --- | --- | --- | --- |
| S1 | DE "Intimate Partner Violence" OR DE "Domestic Violence" OR DE "Dating Violence" OR DE "Battered Females" | Limiters - Publication Year: 2009-2019  Search modes - Boolean/Phrase | **10,560** |
| S2 | DE "Digital Interventions" OR DE "Mobile Phones" OR DE "Randomized Controlled Trials" OR DE "Crisis Intervention" OR DE "Mobile Applications" OR DE "Mobile Health" OR DE "Text Messaging" OR DE "Wireless Technologies" OR DE "Computer Applications" OR DE "Digital Technology" OR DE "Smartphones" OR DE "Intervention" OR DE "Computer Mediated Communication" OR DE "Innovation" OR DE "Information and Communication Technology" | Limiters - Publication Year: 2009-2019  Expanders - Apply related words  Search modes - Boolean/Phrase |  |
| S3 | S1 and S2 | Limiters - Open Access; Publication Year: 2009-2019; Publication Type: Peer Reviewed Journal  Expanders - Apply related words  Narrow by Language: - english  Search modes - Boolean/Phrase |  |
| S4 | DE "Intimate Partner Violence" OR DE "Domestic Violence" OR DE "Dating Violence" | Limiters - Open Access; Publication Year: 2009-2019; Publication Type: Peer Reviewed Journal  Expanders - Apply related words. Search modes - Boolean/Phrase |  |
|  | S2 AND S4 | Limiters - Open Access; Publication Year: 2009-2019; Publication Type: Peer Reviewed Journal    Expanders - Apply related words    Search modes - Boolean/Phrase |  |

**Web of Science – April 4, 2019**

| **#** | **Query** | **Limiters/Expanders** | **Results** |
| --- | --- | --- | --- |
| S1 | ((mHealth OR mobile apps OR eHealth OR mobile applications OR apps) AND (adolescent OR teenagers OR teens OR youth) AND (teen dating violence OR youth violence OR dating violence OR adolescent dating violence)) | All years. Indexes: SCI-EXPANDED, SSCI, A&HCI, CPCI-S, CPCI-SSH, BKCI-S, BKCI-SSH, ESCI, CCR-EXPANDED, IC. | 19 |
